# Effects of recombination on multi-drug resistance evolution in *Plasmodium falciparum* malaria

**DOI:** 10.1371/journal.pcbi.1013401

**Published:** 2025-08-25

**Authors:** Kien Trung Tran, Tran Dang Nguyen, Daniel B. Weissman, Eric Zhewen Li, Sachel Mok, Jennifer L. Small-Saunders, Teun Bousema, Robert J. Zupko, Thu Nguyen-Anh Tran, Maciej F. Boni

**Affiliations:** 1 Institute for Genomics and Evolutionary Medicine, Department of Biology, Temple University, Philadelphia, Pennsylvania, United States of America; 2 Department of Physics, Emory University, Atlanta, Georgia, United States of America; 3 Center for Infectious Disease Dynamics, Department of Biology, Pennsylvania State University, University Park, Pennsylvania, United States of America; 4 Division of Infectious Diseases, Department of Medicine, Columbia University Irving Medical Center, New York, New York, United States of America; 5 Center for Malaria Therapeutics and Antimicrobial Resistance, Columbia University Irving Medical Center, New York, New York, United States of America; 6 Department of Medical Microbiology, Faculty of Medical Sciences, Radboud University Medical Center, Nijmegen, Netherlands; Indian Institute of Science, INDIA

## Abstract

When multiple beneficial alleles at multiple loci are present in a population but not linked together in any one individual, there is no general evolutionary result that determines whether recombination will speed up or slow down the emergence and evolution of genotypes carrying multiple beneficial alleles. Translated to infectious disease control, this evolutionary uncertainty means that when multiple types of drug resistance are present we do not know whether recombination will act more strongly to (1) bring together single-resistant genotypes into multi-drug resistant (MDR) genotypes, or (2) break apart MDR genotypes into single-resistant genotypes. In this paper, we introduce a new version of an established and validated individual-based malaria transmission model where we have added 25 drug-resistance related loci, individual mosquito bites, and mosquitoes feeding on multiple hosts in a single meal (interrupted feeds) allowing for recombination events of different *Plasmodium falciparum* genotypes from different hosts. Recombination among *P. falciparum* genotypes in this model occurs from two sources of variation, multi-clonal infections in single hosts and interrupted feeds on multiple hosts, and we show that 80% to 97% of MDR recombinant falciparum genotypes are projected to occur from single uninterrupted bites on hosts with multi-clonal infections (for malaria prevalence > 5%). Increases in the model’s interrupted feeding rate slowly increase the number of recombination events occurring from interrupted feeds. A comparison of drug-resistance management strategies with this new model shows that, over a 15-year timeframe, triple artemisinin-combination therapies (ACT) strategies show the largest reductions in treatment failures and the longest delays until artemisinin resistance reaches a critical 1% threshold. Multiple first-line therapies (MFT) are second best under these criteria, and ACT cycling approaches are third best. When compared to cycling strategies, MFT strategies generate a greater diversity of recombinant genotypes but fewer recombination events generating MDR and slower emergence of these recombinant MDR genotypes.

## Author summary

Malaria remains one of the world’s leading causes of infectious disease morbidity and mortality despite major improvements in interventions over the past 25 years. One of the biggest challenges in malaria control is drug-resistance management, and management of artemisinin resistance specifically as a large majority of malaria cases today are treated with artemisinin-based combination therapies (ACTs). As drug resistance evolves, we do not know how genetic recombination—which occurs inside mosquitoes—influences this process. In this study, we developed a new individual-based mathematical transmission model of malaria, including 25 loci known to affect drug resistance, to simulate how mosquitoes bite people and how drug-resistant parasites are formed through recombination. Using results from modeling, we found that most parasite recombination occurs as a result of mosquitoes biting hosts infected with multiple genotypes of *Plasmodium falciparum*. A smaller fraction of recombination events result from mosquito interrupted feeds where blood from two human hosts is mixed into a single blood meal. With this new genetically-detailed model, we compared different population-level treatment strategies to investigate which strategies had the largest effects of delaying or slowing down drug resistance. We found that triple ACTs were the most effective at delaying resistance and keeping treatments at high efficacy for longer periods. Multiple first-line therapies (MFT) and cycling policies ranked second and third, respectively. MFT policies also helped, by a small margin, at reducing and delaying the rise of double-resistant and triple-resistant parasites through recombination.

## 1 Introduction

*Plasmodium falciparum* malaria caused an estimated 249 million symptomatic malaria cases and 608,000 deaths worldwide in 2022 [1]. The majority of the world’s malaria infections and deaths occur in Africa, in children under the age of five, despite the fact that highly efficacious artemisinin combination therapies (ACTs) have been available worldwide as first-line therapy for falciparum malaria for nearly twenty years. The emergence of artemisinin-resistant malaria parasites in East Africa in the middle of last decade has the potential to undermine our current approach to malaria control. As of mid-2024, five different genotypes – with mutations in the *pfkelch13* gene that is associated with slower parasite clearance by ACTs – are known to have spread successfully in Rwanda, Uganda, Tanzania, Kenya, and Ethiopia and reached local genotype frequencies above 0.20. Mathematical modeling analyses project that these genotypes frequencies will go up quickly over the next the decade [2,3].

As for all pathogens, the relationship between genotype and drug-resistance phenotype in *P. falciparum* is complex. The magnitude of resistance benefits varies across loci and drugs. Multi-allelic resistance types are known. Pleiotropy occurs at several loci conferring opposing properties of drug resistance and drug sensitivity depending on the drug used. A minimum of twenty-five loci are known to have some effect on conferring drug resistance to artemisinin and the partner drugs used in ACTs, and epistatic effects are known to exist but difficult to measure because genotype information is typically not presented in therapeutic efficacy studies and randomized controlled trials of ACT efficacy. To add to this complexity, two types of transmission events are known to provide opportunities for recombination among falciparum genotypes: mosquito bites on individuals infected with multiple falciparum genotypes, and interrupted mosquito feeds where a blood meal is initiated on one host but completed on another. The influence of recombination will be stronger in high-transmission regions than in low-transmission regions [4,5], but there are still many challenges in understanding how recombination affects the diversity and evolution of drug-resistant falciparum genotypes [6–11] and whether recombination tends to speed up or slow down drug-resistance evolution [11–13].

Understanding and correctly modeling this evolutionary complexity is critical for producing forecasts for *P. falciparum* evolution in Africa over the next decade. We have upgraded an individual-based simulation of falciparum malaria epidemiology and evolution [14] to add explicit chromosome structure, drug-resistance phenotype information for 25 loci, and individual (trackable) recombination events that can occur as a result of bites on multi-clonal hosts or interrupted feeds. We use the model to ask (1) which of the two recombination processes is more common, (2) which drug distribution strategies are best at delaying and slowing resistance in the context of these recombination processes [15–17], and (3) which strategies generate more/fewer double-resistant and triple-resistant genotypes through recombination [12]. The development of a multi-locus model, covering all known loci associated with reduced efficacy of ACTs, is necessary to carry out practical evaluations of drug-resistance response policies in specific African countries that will be implementing them this decade.

## 2 Methods

The model used in this study builds upon the original individual-based model of *P. falciparum* evolution and epidemiology developed by Nguyen et al (2015, 2023) which has been utilized in various studies evaluating drug rotation strategies, deployment of multiple first-line therapies (MFT), and triple ACT strategies [14,15]. The model is a stochastic individual-based discrete event simulation where individuals (humans) can be bitten by mosquitoes and infected with any number of malaria ‘clones’ or ‘genotypes’. Individuals have varying susceptibility to mosquito bites and variable onward transmissibility to mosquitoes that depends on the density of malaria parasites in their blood. Malaria infections or clones are cleared either by a patient’s immune system, over a period of 60–300 days, or more quickly by drug treatment given to symptomatic patients based on a drug coverage parameter (the percentage of symptomatic patients that seek and receive antimalarial treatment). For treated patients, pharmacokinetics follow a one-compartment clearance model with variable absorption levels among patients and pharmacodynamics follow a standard Hill equation. At 28 days post-treatment, patients are classified as having failed treatment if their parasitaemia is higher than ten asexual parasites per microliter of blood. Treatment failure probability is drug-dependent and genotype-dependent [17].

The previous version of the model had a genotype structure limited to 64 or 128 predefined genotypes modeling resistance markers in *P. falciparum* chromosomes 5, 7, 13, and 14. With the recent emergence of complex and diverse resistance patterns – especially in the *pfkelch13* and *pfcrt* genes – a more flexible model was necessary to address these challenges effectively. We introduced new features into this model (called model version 5.0) which include a chromosome-based genotype structure and a ‘representative mosquito cohort’ to keep track of the diversity of sampled genotypes and the effects of recombination on the evolution of drug resistance. The following sections describe new or upgraded components of the model.

### 2.1 Chromosome-based locus structure

In the previous model, a genotype was defined as a string with six characters representing six different loci that could mutate in any *Plasmodium falciparum* genome. These six loci were K76T in *pfcrt*, N86Y and Y184F in *pfmdr1*, C580Y in *pfkelch13*, and copy number variation (CNV) for *pfmdr1* and the *plasmepsin-2,3* genes on chromosome 14. For version 5, we extended the genotype structure to cover all 14 chromosomes of *Plasmodium falciparum* and introduced a new string format to represent genotype in the model. In this format, a wild-type genotype is denoted by:

||||NYD1||KTHFIMG||||||FNCMYRIPRPCRA|1

where the thirteen vertical bars denote breaks between chromosomes, in order, and each character represents an allele or a copy number in a specific gene. This string serves as a key in a key-value pairing (i.e., a ‘map’ or ‘dictionary’ associative array structure) to obviate the memory needs of permanently storing information on millions of genotypes. Entries are added and removed from this map as genotypes appear or disappear in the simulation. Full details of this new genotype structure and the number of alleles integrated into the new model are provided in Section 2 in S1 Text.

A masking structure was built into the simulation to allow for mutation at only some loci. This was done to allow the simulation to keep genetic variation restricted to what is observed in the field. For example, a maximum of one *pfkelch13* mutation is allowed in all simulations here since double mutants have not been observed yet. In the results presented in this paper, only 10 mutation types or CNVs are enabled: two loci and copy number variation in *pfmdr1*, five loci in *pfcrt*, a single amino-acid change in *pfkelch13*, and copy number variation in the plasmepsin genes.

### 2.2 Assumption of non-epistasis among loci

Epistatic interactions among genes – where the effect of a pair or a group of mutations is different than the sum of their individual effects – are both critical and difficult to measure when attempting to model and forecast evolutionary trajectories. In this model version, we begin with an assumption of non-epistasis among loci (meaning that the phenotypic effects of mutations are independent of one another and follow a simple multiplicative fitness model) and we add in epistatic effects for pairs of loci for which there is sufficient supporting evidence. This is done in the pharmacodynamic component of the model, where the shift in the EC50 factor (drug concentration at which parasite killing is 50% of maximum) is assigned in a multiplicative manner when multiple mutations accumulate that increase a parasite’s resistance level to a particular drug (see Section 13 in S1 Text). In doing this, we reduce the time and space complexity of computing drug efficacy on 32 million potential genotypes in the model. For mutational pairs where epistatic effects are known or have been inferred (as in Supplement 2 of [14]) the inferred fitness value (with epistasis) overrides the multiplicative fitness value calculated under an assumption of no epistasis. The essential description of genotype-phenotype mapping is described in Supplement 2 of Nguyen et al (2023) [14], with additional components described in Section 13 in S1 Text.

### 2.3 Post-recombination mosquito cohort (PRMC)

The new model includes a representative mosquito population but does not model all individual mosquitoes in a population. The representative mosquito population is modeled via a cohort of 100–500 individual mosquitoes that carry parasites in the post-recombination phase (ookinetes, oocysts, sporozoites) of parasite development in the mosquito. This mosquito cohort carries representative genotypes from the population of human hosts in the model. Mosquitoes in the cohort are populated with falciparum genotypes every day via biting and parasite sampling events that include interrupted feeds through which the mosquitoes can sample parasites from two different hosts. The interrupted feeding rate (IFR) is defined as the proportion of completed mosquito feeds that occurred on two or more (not necessarily infected) humans. Free recombination among falciparum’s 14 chromosomes occurs after mosquito sampling, and a single genotype is chosen through this random process to be assigned to the sporozoites population that will infect human hosts 11 days later. There is no recombination within a gene on a particular chromosome; all genes are on different chromosomes in this model version. As a result, the mosquito cohort acts as a pool for introducing novel recombinant genotypes into the human population. The mosquito cohort has two configurable factors: cohort size and the interrupted feeding rate. Interrupted feeding rate is set to between 0% and 20%; this is the percentage of fed mosquitoes that have blood from two or more humans, regardless of human infection status. A cohort size of 100 means that 1100 mosquitoes are tracked at any time as 11 days of mosquito history must be tracked. Details of this transmission mechanism and mosquito cohort design are described in Section 8 in S1 Text.

### 2.4 Counting recombinations in interrupted feeds and from multiclonal infections

To determine what percentage of recombination events occur due to interrupted feeds, we counted recombination events that produce double-resistant genotypes from single-resistant genotypes. A base scenario was run where recombination among clones present in a single host was not permitted in the simulation (this is called the “within-host recombination turned off” mode of the model); in this scenario, all recombination occurs as a result of interrupted feeds. Every set of base scenario runs was compared to a set of ‘normal runs’ where the within-host recombination is “turned on” in the model (as it is in real life). This approach was taken to confirm that implementation of the interrupted-feeding mechanism in the code was done correctly. After completing the analysis, it was apparent that counting different types of recombination events could be done either by turning within-host recombination “on” or “off”, or equivalently by comparing results between IFR = 0.0 and IFR = 0.2 scenarios.

### 2.5 Scenario calibration

To calibrate the model, we adjusted the mutation probability so that the 580Y allele frequency in the model would reach 0.01 after 7.0 years in a population of 100,000 individuals, with a *P. falciparum* prevalence of 10% and with 40% of malaria cases receiving dihydroartemisinin-piperaquine (DHA-PPQ) as first-line treatment. It is clear that the time from a *pfkelch13* mutant’s first successful appearance (e.g., first successful onward transmission) to its 0.01 allele-frequency “emergence point” must be less than 15 years in Cambodia and less than 10 years in Rwanda, as artemisinin drugs were not used in pre-1980 Cambodia or pre-2004 Rwanda. This time-to-emergence must also be longer than “several years” as ACT coverage was not high enough in either setting during early ACT deployment to bring rare alleles to high frequency quickly. The seven-year emergence timeline is chosen here as a midpoint of this plausible range, as in previous publications [2,17–19].

Following this calibration, simulations were configured with a population size of 100,000, and *P. falciparum* prevalence rates set at 5% and 25% as representative of hypoendemic and mesoendemic malaria, respectively. Model simulations are run for 10 years as burn-in with 50% of the population treated with a generic 80% efficacy drug and 50% of malaria cases untreated; no private-market drugs are used during this period. The transmission parameter is calibrated so that malaria prevalence in 2–10 years olds (abbreviated PfPR here) reaches either 5% or 25% at the end of the burn-in period. This is intended to stabilize the simulation’s dynamics to an African epidemiological scenario in 2005 just prior to ACT deployment. Initial genotypes in simulation runs were wild-type alleles for *pfkelch13*, *pfmdr1*, and *pfpm2,3*, except for *pfcrt* 76T and a 50/50 ratio of *pfmdr1* N86 and 86Y. Genotypes with the N86 allele are selected for during burn-in due to the model’s built in cost of resistance (*c*_*R*_) [18,20]. Parasites carrying drug-resistance alleles carry a fitness cost *c*_*R*_ (relative to wild-type parasites) in the absence of drugs; see Section 6 in S1 Text. At the end of burn-in, the genotype balance between 76T-N86-Y184 (“TNY”) and 76T-86Y-Y184 (“TYY”) genotypes is typically around 50% - 55% TNY when *c*_*R*_ = 0.0005 and 70% - 90% TNY genotypes when *c*_*R*_ = 0.005.

### 2.6 Treatment strategies

After ten years of burn-in, different drug-resistance mitigation strategies are put into place and the model is run for an additional fifteen years. An assumption of 75% treatment coverage is made. A 15-year range was chosen so that a fair comparison could be carried out between MFT with three ACTs and a 5-year cycling policy that deployed all three ACTs for exactly five years each. During these 15 years, the outcome measures collected are (a) number of treatment failures (TF), (b) the time it took for artemisinin-resistant alleles to reach an 0.01 genotype frequency, (c) the number of recombination events that generated double-resistant genotypes from single-resistant genotypes, and (d) the number of recombination events that generated triple-resistant genotypes from single-resistant and double-resistant genotypes. It was assumed that artemether-lumefantrine (AL), artesunate-amodiaquine (ASAQ), and DHA-PPQ were available as first-line therapy for antimalarial treatment.

Four common and previously analyzed [14,15] treatment strategies were evaluated: (1) multiple first-line therapies (MFT) where all three ACTs are distributed simultaneously in equal amounts with 33.3% of malaria cases treated by each ACT; (2) 5-year cycling where first-line therapy is changed every five years, regardless of the treatment failure rate at the end of every five-year period; (3) adaptive cycling where first-line therapies are switched when the current therapy’s treatment failure rate rises above 10%; and (4) deployment of a triple ACT, either dihydroartemisinin-piperaquine combined with mefloquine (DHA-PPQ-MQ) or artemether-lumefantrine combined with amodiaquine (ALAQ). The current treatment failure rate for each therapy is calculated over a 60-day window of past treatments. In the adaptive cycling approach, a drug switch takes place one year after TF rises above 10% and a new therapy must be used for one year before another switch occurs.

Two new approaches are evaluated in this analysis. First, cycling approaches are split into whether the long half-life partner drugs are used first in the cycling strategy with the short half-life drugs used last – this is an LMS (long-medium-short) strategy – or whether short half-life partner drugs are used first and long half-life drugs last (SML). In an LMS cycling strategy, the order of the therapies is DHA-PPQ first, ASAQ second, AL third. Under SML, the order is AL first, ASAQ second, DHA-PPQ third. Past cycling analyses [12,15] used LMS as this approach reduces malaria prevalence more and earlier than an SML approach. Second, since MFT is non-adaptive, an adaptive MFT approach was created. The adaptive MFT approach begins with 33.3% of cases treated with each ACT, and with readjustments made every two years based on current treatment failures (with a one year delay allowed for implementation). In other words, treatment failure percentages for each ACT are measured at years 2, 5, 8, and 11, and adjustments to ACT distribution are made in years 3, 6, 9, and 12. The purpose of this adaptive MFT approach is to increase the usage of high efficacy therapies and decrease the usage of low efficacy therapies as the resistance landscape changes. Implementation of this scheme would be challenging in a real-world setting but this analysis can tell us how much room for improvement there is for traditional fixed MFT approaches. Details on how the distributions are updated are provided in Section 11 in S1 Text.

## 3 Results

Our new individual-based 25-locus epidemiological model for *P. falciparum* malaria can faithfully replicate classic epidemiological relationships for malaria, including (1) the relationship among prevalence, incidence, and the entomological inoculation rate (EIR) (Figs N and O in S1 Text), (2) the relationship between age-specific incidence and EIR (Fig L in S1 Text), (3) the relationship between EIR and the multi-clonality of infections (Fig P in S1 Text), (4) the time-to-emergence period of artemisinin resistance based on approximate timelines in southeast Asia (Fig Q in S1 Text), and (5) the efficacies of ACTs on a large range of genotypes known to be partially resistant to artemisinin or the partner drugs co-formulated in ACTs [21–24]. This model was created to have an accurate representation of the *P. falciparum* genome’s loci associated with drug resistance and to have an accurate representation of the recombination process that combines drug-resistance mutations and breaks them apart. With an extensive set of simulations we verified that the presence of this new recombination mechanism in the model takes linkage disequilibrium to zero (example in Fig D in S1 Text) when there is no treatment and when fitness costs are set to zero.

**Fig 1:**
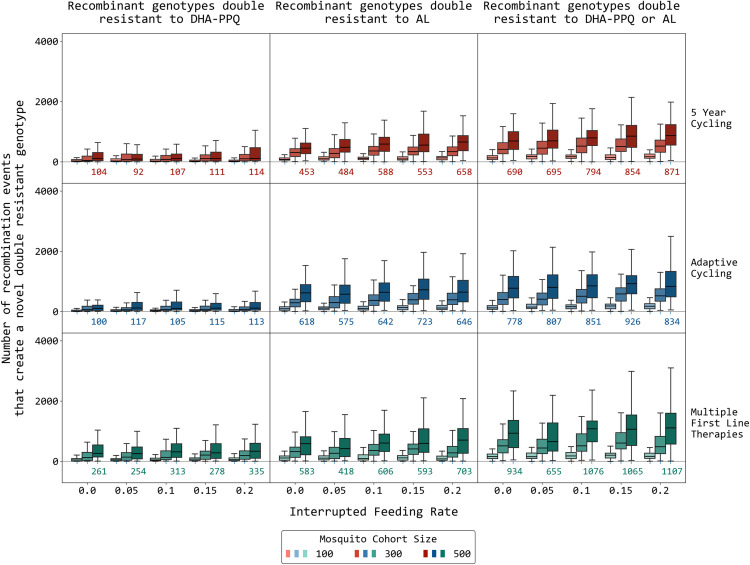
The number of recombination events through year 15 that produce novel recombinant double-resistant genotypes. A recombination event is counted when (1) the recombinant offspring has both a pfkelch13 mutation and at least one partner-drug associated resistance mutation, and (2) the parental genotypes that recombined carried resistance alleles to only one drug (i.e., one parent carried a pfkelch13 mutant and was fully susceptible to the partner drug, while the other parent was wild-type at the pfkelch13 locus and carried at least one mutation to a partner drug). The three rows of panels show recombination counts under three different treatment strategies; cycling is LMS. The x-axis in each panel shows the mosquito interrupted feeding rate. The three boxplots in each group show the median/IQR for number of recombination events when the model uses a mosquito cohort size of 100, 300, or 500 (from left to right). Numbers beneath the boxplots on the right indicate the median number of recombination events for a mosquito cohort size of 500. Note that the absolute numbers of recombination events shown in this figure are only meaningful for a mosquito cohort of a particular size. Malaria prevalence is 5% and the daily per-allele fitness cost of resistance is 0.0005. Even though MFT has more recombination occurrences for DHA-PPQ double-resistants, these double-resistants are not necessarily successful and do not spread more quickly as can be seen in Fig F in S2 Text.

In a range of model scenarios, recombination events generating double-resistant genotypes to AL or DHA-PPQ increase – irregularly and slower than linearly – with an increase in the model’s interrupted feeding rate (IFR); see Fig 1. Because the initial genotypes chosen in our simulation were amodiaquine-resistant 76T Y184 genotypes, recombination events generating double-resistants to ASAQ were not seen in the simulations due to the lack of single-resistant genotypes to artemisinin that carried no amodiaquine resistance. Initial recombination counts in a range of generic scenarios made clear that the IFR is unlikely to be the major driver of recombination, as increasing the IFR from 0.0 to 0.2, which is close to the maximum observed in field studies [25–34], showed a 17% increase (under MFT) and a 26% increase (under the two cycling policies) in the total number of double-resistance-generating recombination events recorded in the simulation. Note that the recording of a recombination event is specific to the partner drug, and that only one resistance mutation (or CNV) is required for a genotype to be counted as partner-drug resistant. Additionally, note that in the simulations done for Fig 1, lumefantrine has three mutations associated with resistance while piperaquine has five. When compared to cycling policies, MFT appears to generate more DHA-PPQ double-resistants through recombination but similar numbers or fewer AL double-resistant through recombination (Figs 1 and A–D in S2 Text). The reasons appear to be that (1) the number of loci that confer resistance to PPQ is higher than the number of loci conferring resistance to lumefantrine, generating more opportunities for recombination under MFT, and (2) DHA-PPQ is used first in the two cycling strategies ensuring that it is used at a time when *pfkelch13* mutants are at low frequencies, reducing the chance of generating a double-resistant through recombination. Even though MFT generates a larger number of DHA-PPQ double-resistant genotypes through recombination, the time for this double-resistant genotype to reach 0.01 genotype frequency is longer than under cycling policies (Fig H in S2 Text).

**Fig 2:**
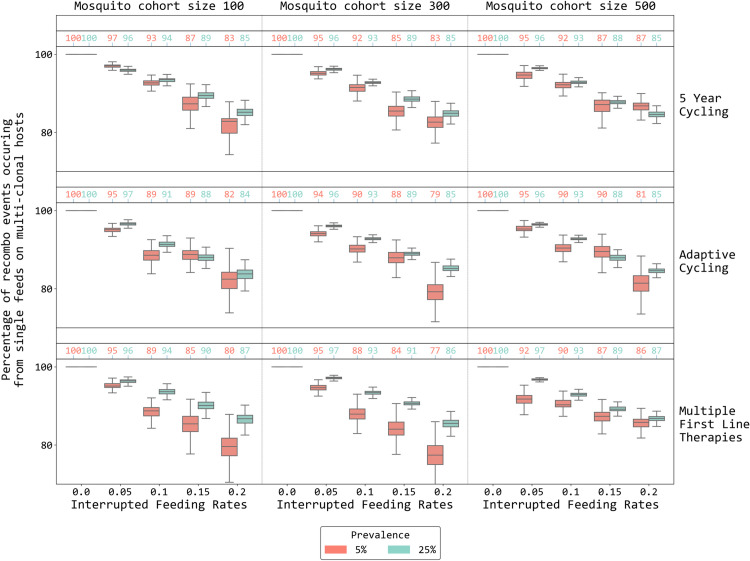
The percentage of recombinations producing double-resistant genotypes that come from single feeds on multi-clonal hosts. The percentage is computed by dividing the number of recombination events (within-host recombination turned off) by the number of total recombination events (within-host recombination turned on) and subtracting from 100%; this is done via sampling with replacement from the empirical recombination counts produced by the simulation. Panel columns indicate different mosquito cohort sizes while panel rows show different strategies applied; cycling is LMS. The x-axis indicates interrupted feeding rates of the mosquito while the y-axis shows the percentage of recombination events that can be attributed to single feeds on multi-clonal hosts. Red and green depict the percentage computed at 5% and 25% prevalence respectively. Numbers above the boxes are the medians for those box plots. In this figure the daily cost of resistance is 0.0005.

When comparing model runs where recombination via single uninterrupted mosquito feeds has been “turned off” to normal runs where recombination can occur in either single feeds or interrupted feeds (as in real life) a count of recombination events by type shows that a median of 80% to 97% of recombination events generating new genotypes (here double-resistants) occur from single uninterrupted feeds (Fig 2). There did not appear to be large differences when comparing across treatment strategies or across mosquito cohort sizes of 100, 300, or 500, suggesting that a mosquito cohort size of 100 may be adequate for capturing malaria genetic diversity and recombination behavior in a population of 100,000 individuals. The higher or lower frequency of recombination through one process (single feeds) compared to another (interrupted feeds) depends on the fraction of the host population that is carrying multi-clonal infections, the overall genetic diversity of malaria, and the proportion of mosquito feeds that are interrupted. In the 5% and 25% prevalence scenarios evaluated here, 12.1% (IQR: 10.4 - 14.6) and 22.5% (IQR: 20.1 – 25.0) of hosts, respectively, have multi-clonal infections. A maximum of 20% of mosquito bites are interrupted, and only 5% or 25% of these will be feeds where *both* hosts are infected, showing why mutliclonality likely plays a larger role in generating recombinant genotypes of malaria. Note that the level of mutliclonality declines with lower prevalence, but so does the probability of a mosquito feeding on two infected hosts.

### 3.1 Strategy comparisons

The evolutionary effects of treatment strategies on drug-resistance evolution have been studied with a range of different models from classic population-genetic approaches [10,35] to modern spatially-structured individual-based models [2,16]. The general conclusions drawn from these analyses are that maximizing environmental variability slows down drug-resistance evolution. In the language of malaria treatment, parasite populations that encounter more drugs in short time periods will show the slowest or most delayed drug-resistance evolution [36,37]. However, it is not known whether recombination events affecting these evolutionary trajectories are expected to be more common or less common under one treatment strategy compared to another.

**Fig 3:**
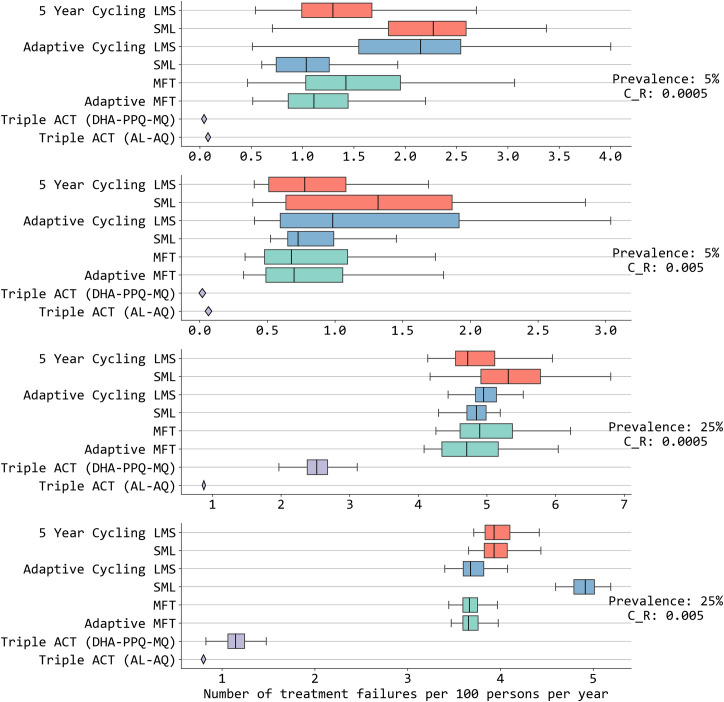
Comparison of annual numbers of treatment failures (NTF) among different strategies. Results shown for 15 years of simulation after applying different treatment strategies in four different prevalence and cost-of-resistance settings. The x-axis shows treatment failures per 100 person per year. Different colors are for different treatment strategies: 5-year cycling (red), adaptive cycling (blue), multiple first-line therapies (green) and triple ACT (violet). LMS indicates the order of drugs used in cycling strategies based on half-life of partner drugs (**L**ong: DHA-PPQ, **M**edium: ASAQ, **S**hort: AL). A single diamond corresponds to a boxplot where the IQR range is smaller than 0.05. Among these strategies, triple ACT use stands out with the lowest number of treatment failures for both low and high prevalence. MFT and adaptive MFT generally (but not always) have lower NTF values than cycling approaches. The mosquito cohort size is 100 and the interrupted feeding rate is 20%.

Across two prevalence scenarios (5% and 25% PfPR) and two scenarios with differing costs of resistance (17% and 84% annual fitness cost), MFT policies had marginally better outcomes than cycling policies in terms of the number of treatment failures (NTF) they were projected to generate over a 15-year period (Figs 3 and E in S2 Text). In general, cycling policies did not have predictable outcomes when comparing adaptive cycling to 5-year cycling and LMS ordering to SML ordering of therapy deployment. MFT policies tended to have equal or slightly lower NTF ranges than cycling policies – median NTF for MFT ranged from 49% lower to 4% higher when compared to cycling policies for three of the four prevalence/fitness-cost combinations. At 5% prevalence and *c*_*R*_ = 0.0005, MFT-cycling comparisons were inconclusive with two cycling policies projected to have higher NTF counts and two cycling policies projected to have lower NTF counts than MFT.

Cycling policies in some instances showed improvements over MFT policies. As noted in previous studies [16,17], MFT policies are predicted to delay and slow down drug-resistance evolution by a larger margin than cycling policies when all therapies (1) have equal efficacy currently and (2) are projected to have equal efficacy into the near future when drug resistance is expected to evolve. In our simulation set-up, certain amodiaquine-resistant genotypes are fixed at the beginning of the simulation (as they would have been in 2005 when ACTs were introduced) and AL efficacy is high (>92%) on these genotypes; thus, an adaptive cycling policy that uses AL first is likely to have high cure rates over a long period and may thus be favored over an MFT policy where all three ACTs are used equally. In the LMS 5-year cycling approach in Fig 3, DHA-PPQ is high-efficacy, used first, and replaced after five years before *pfkelch13* variants are able to emerge and erode its efficacy; under SML 5-year cycling however, DHA-PPQ is used in years 10–15 when *pfkelch13* are likely to be circulating at higher levels, resulting in the highest levels of treatment failure seen among the three ACTs. This explains why LMS has more favorable drug-resistance mitigation outcomes than SML in this particular scenario, but there does not appear to be a general rule on which ordering of ACTs is optimal in a cycling approach (see *Discussion*). Note that adaptive cycling policies may only use one or two therapies in a pre-defined period if resistance evolution is slow (Fig P in S2 Text) further complicating the comparison of which therapy to use first. Additionally, adaptive cycling policies sometimes have equal resistance and TF outcomes to MFT policies, but adaptive cycling approaches are sensitive to the delay period between identifying resistance and switching therapies, leading to substantial increases in treatment failure counts when delays are long (Fig I in S2 Text). Adaptive MFT policies showed small and inconsistent improvements over fixed MFT policies, largely due to these adaptive policies’ small adjustments in drug distribution making their overall therapy use similar to a fixed MFT policy.

By far the most conclusive result, via a direct comparison of treatment failure counts, was that deployment of triple ACT was projected to have substantially lower NTF over a 15-year period than any cycling or MFT approach. Deployment of either triple ACT showed treatment failure counts that were 48% to 97% lower than the other strategies, confirming the findings of Nguyen et al (2023) and Zupko et al (2023) [2,17].

**Fig 4:**
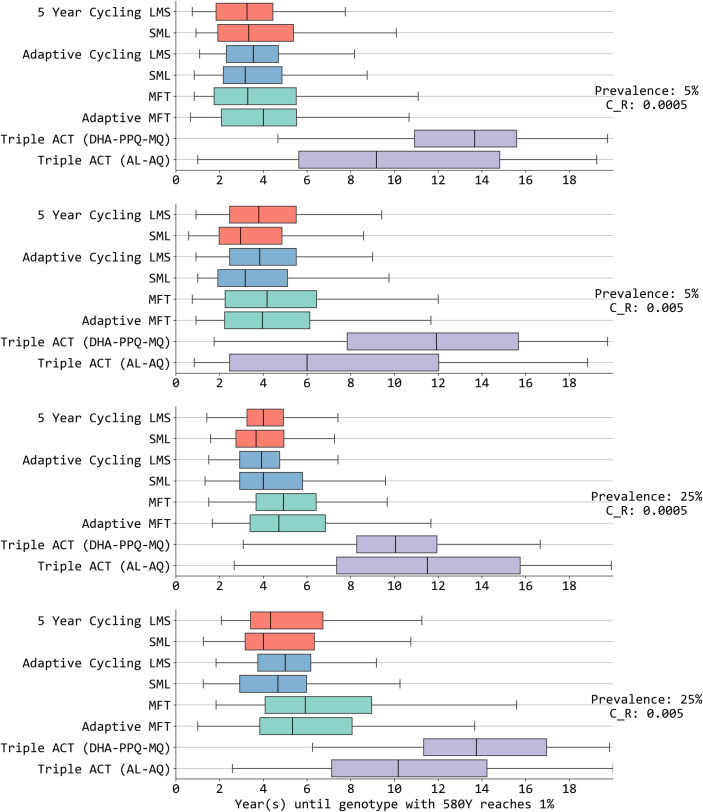
Number of years (x-axis) until a single-mutation pfkelch13 variant reaches 0.01 allele frequency. Results are shown for four different prevalence and cost-of-resistance settings. Different colors are for different treatment strategies: 5-year cycling (red), adaptive cycling (blue), multiple first-line therapies (green) and triple ACT (violet). LMS indicates the order of drugs used in cycling strategies based on half-life of partner drugs (**L**ong: DHA-PPQ, **M**edium: ASAQ, **S**hort: AL). Triple ACT use stands out with the longest delay until artemisinin-resistance emergence. MFT and adaptive MFT are generally (but not always) associated with longer emergence times than cycling approaches. The mosquito cohort size is 100 and the interrupted feeding rate is 20%.

When looking at a drug-resistance prevention or delay metric – the time until an artemisinin-resistant genotype reaches 0.01 frequency in the host population (T_.01_) – MFT policies generally outperformed cycling policies, and LMS appeared to have small advantages over SML cycling policies. In six of the eight LMS-to-SML comparisons in Fig 4, LMS rotations had times to emergence that were 7.1% to 28.2% longer than SML rotations; in the remaining two comparisons T_.01_ was 2.1% and 2.5% shorter for LMS. If this behavior can be shown to be robust, it may be the result of piperaquine resistance (emerging first under LMS) requiring more mutations than lumefantrine resistance (Fig O in S2 Text). For this same reason – with the three therapies not being identical in their (a) current efficacy, (b) their future resistance profiles, and (c) the number of known mutations conferring resistance – cycling policies are able to sometimes outperform MFT policies if they use their best therapies first. As an example, at 5% prevalence and *c*_*R*_ = 0.0005, SML 5-year cycling had an emergence time T_.01_ that was 1.3% longer than under MFT, and LMS adaptive cycling had a T_.01_ that was 7.6% longer than under MFT. For the remaining 14 of 16 comparisons between cycling and non-adaptive MFT in Fig 4, MFT had times to emergence that were 1.3% to 47.9% longer.

In the scenarios parameterized here, the recombination rate does not appear to have a large effect on when various resistant types appear. This can be seen by comparing IFR = 0.0 to IFR = 0.2 in Fig 1. Fig Q in S2 Text shows the emergence time (T_.01_) comparisons with within-host recombination turned on and off, and the median emergence times do not appear to differ; long-term evolutionary dynamics out to 30 years do not seem to differ between high and low recombination scenarios (Figs R and S in S2 Text). Strategy comparisons do not differ if prevalence measurements (traditional or sub-microscopic) are used instead of treatment failure counts (Figs T and U in S2 Text).

The longest delay in artemisinin resistance’s time to emergence occurred, by a large margin, under triple ACT strategies. When using triple ACTs, the model projections showed that the median time until 0.01 genotype frequency of artemisinin resistance was 1.7 times to 3.4 times longer than under the best cycling or MFT policies.

### 3.2 Risk of multi-drug resistance

**Fig 5:**
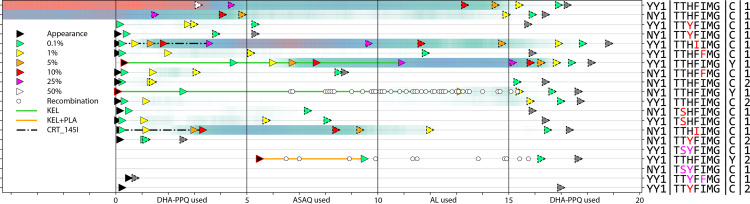
Evolution from a single 20-year model simulation of a 5-year cycling strategy with DHA-PPQ used in the first five years, ASAQ in the following five years, AL during years 10-15, and DHA-PPQ again in years 15-20. Prevalence is 5% and cost of resistance is set to c_R_ = 0.0005. Graph above shows evolution of 20 chosen genotypes (rows) with colored triangles showing genotype frequency milestones (see legend at left). Triangles with dashed borders indicate that a genotype frequency is decreasing, and the dark gray triangles indicate that a genotype has disappeared. The darker sea green and lighter sea green shadings correspond to genotype frequencies in the 0.05 to 0.25 range. Circles show when a genotype is generated through a recombination event, and the three circles on the orange KEL + PLA lineage are early recombination events. Genotype codes at right show genes on chromosomes 5, 7, 13, 14, separated by vertical lines. The first group of alleles (chromosome 5) shows the alleles at position N86Y, Y184F, and the copy number of the pfmdr1 gene. The second group of alleles (chromosome 7) shows seven pfcrt loci with mutations at positions 76, 93, 97, 145, 218, 343, and 353 highlighted in red (single mutants) and pink (double mutants); these are the key mutations (except position 76) known to reduce PPQ efficacy. Colored lines show certain key lineages like the KEL-lineage where a single pfkelch13 mutation is present but plasmepsin copy number (the “1” on the RHS) has stayed at single copy. The “145I” lineage (seventh from bottom) is associated with substantial reduction in PPQ efficacy and increases quickly from years 0 to 5.

To evaluate the risk of early emergence of multi-drug resistant (MDR) genotypes, we counted *early recombination events* that generated double-resistant or triple-resistant genotypes. An *early recombination event* that generates a double-resistant genotype (from two single-resistants) is defined as one that occurs before the double-resistant reaches 0.01 genotype frequency. In other words, this count captures the occurrence of recombination when it is likely to have its largest impact – during a time period when double-resistance is still rare. Similarly, an *early recombination event* generating a triple resistant (from a single- and a double-resistant) is one that occurs prior to the triple resistant reaching 0.01 genotype frequency. MDR genotypes can be produced by either recombination or mutation, and they can increase in frequency due to selection pressure; all of these processes can be individually tracked during a simulation run as shown in Fig 5. The three recombination events (open circles) shown on the orange line of years 5–10 of this figure are early recombination events.

**Fig 6:**
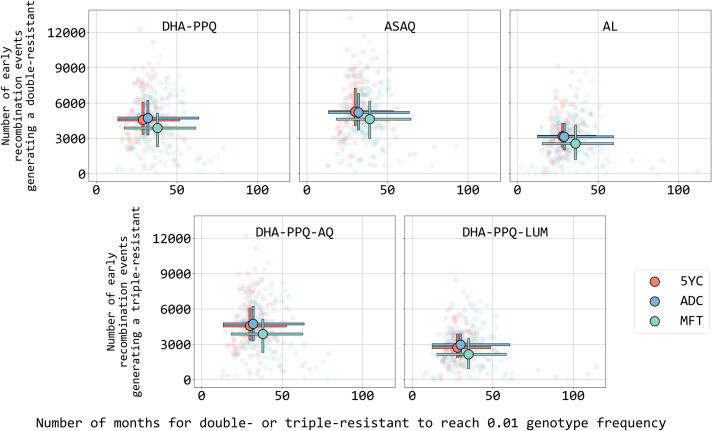
Number of recombination events (y-axis) creating a double-resistant or triple-resistant genotype to a particular ACT or triple ACT (see text inside each panel) that occur prior to the time that the frequency of this MDR genotype reaches 0.01. The x-axis shows the number of months, starting from first appearance, for each MDR genotype to reach 0.01 genotype frequency. Circles show medians and bars show interquartile ranges from 100 simulations from three different treatment strategies: MFT (green), 5-year LMS cycling (red), and LMS adaptive cycling (blue). Prevalence is 5%, c_R_ = 0.0005, mosquito cohort size is 100, and the interrupted feeding rate is 20%. All 300 points are shown in light color in the background.

The total count of early recombination events generating double resistance (Figs 6 and J–L, top panels in S2 Text) is 11% to 21% lower under MFT than under cycling policies, despite the emergence times being longer under MFT. As in Li et al [12], simulations run under an MFT strategy do not appear to generate any additional MDR emergence risk even with all therapies deployed simultaneously (Fig N in S2 Text). The likely reason for this effect is that MFT will tend to keep novel alleles rare for longer periods when compared to cycling policies, making recombination events ‘doubly rare’ when they have to recombine two rare types to create a double- or triple-resistant. Similarly, the total count of early recombination events generating triple resistance is 15% to 27% lower under MFT than under cycling policies (Figs 6 and J–L, bottom panels in S2 Text). Note that MFT policies generate a larger number of unique genotypes through recombination (Fig M in S2 Text) during the course of the simulation but a lower absolute number of drug-resistants generated through recombination.

Times to reach certain triple resistance milestones can only be counted for artemisinin/piperaquine/amodiaquine triple resistance and artemisinin/piperaquine/lumefantrine triple resistance. As noted previously [17], the triple ACT ALAQ does not naturally drive any known triple resistant genotype as all currently known resistance markers for these drugs indicate that lumefantrine-resistant parasites will be amodiaquine-sensitive and that amodiaquine-resistant genotypes will be lumefantrine-sensitive. For this reason, only two triple resistant genotypes are tracked in Fig G in S2 Text which shows that time to triple resistance is 56% to 95% longer under MFT than under cycling polices. Using DHA-PPQ-MQ as first-line treatment, time to emergence of the triple-mutant is 1.3 to 2.3 times longer than under MFT or cycling policies. Note that in all these scenarios the triple mutant starts at zero when the simulation starts; pre-existence of a triple-mutant is a risk factor for deploying triple ACTs [17].

## 4 Discussion

With a novel and more genetically accurate model of drug-resistance evolution in *Plasmodium falciparum* malaria, we are able to identify which recombination process in malaria has a larger effect on generating multi-drug resistant genotypes through recombination – specifically, we find that recombination in falciparum parasites is primarily generated by multi-clonal infections and less often by interrupted mosquito feeds. Additionally, a comparison of drug-resistance management approaches with this new model shows that triple therapy outperforms MFT, and that MFT outperforms cycling approaches, using a range of different outcome measures; this is consistent with our previous analyses comparing these three approaches [2,19]. Finally, MFT strategies do not appear to accelerate MDR evolution when compared to cycling approaches, reaffirming a recent result on this specific question [12].

There continues to be a need for the development of general theory on the question of whether recombination acts more strongly to bring drug-resistance mutations together into MDR genotypes or to break apart MDR genotypes into less resistant offspring. On the one hand, one could argue that when drug resistance is rare recombination should have a stronger effect on breaking apart multi-genic drug resistant genotypes than it does at putting them together. The rationale behind this is that a multi-genic resistant genotype faces the possibility of recombination (and thus breaking apart of multiple resistant alleles in the same genome) in at least 10% of transmission events (this is the multi-clonal rate at 5% prevalence). Recombination bringing two rare resistant alleles together requires two rare types to be sampled by the same mosquito – something that is *O*(*x*^2^) rare when single-resistants are circulating at genotype frequency *x*. The counter-argument is that higher recombination rates should lead to *earlier* emergence of the first recombinant double-resistant genotypes [38]. Even if recombination breaking up these MDR types slows down their subsequent spread, this may be a relatively minor effect if positive selection is strong enough, meaning that the net effect would be to hasten the evolution of resistance [11,13,39]. To the best of our knowledge, it is not known how these forces balance each other out in the specific case of malaria, although in general models adaptation is usually fastest at recombination rates intermediate between asexuality and free recombination [11,13,39], i.e., intermediate rates of recombination may well accelerate MDR evolution but for malaria specifically we do not know where this intermediate range lies.

A second open question in drug-resistance evolution that does not appear to have a general treatment in the literature is the effect that the order of drugs in a cycling approach has on drug-resistance outcomes. Traditional analyses in this field [7,40,41] have assumed that the considered drugs or antibiotics are equal. In reality however, different drugs can have different (1) efficacies, (2) efficacies on future resistant genotypes, (3) mutation rates to resistant types, (4) numbers of mutations conferring resistance, and (5) pharmacokinetic clearance properties. When all five of these properties are equal among the considered therapies, past analyses have shown that MFT outperforms cycling policies; however when these quantities vary among drugs we may see situations when using the ‘best drugs first’ in a rotation strategy is preferable to MFT [16]. When variation exists across all of these properties (as for currently used ACTs) it is not clear which ACT has some optimal and undefined set of characteristics of high treatment success and avoidance of high-grade resistance evolution. A general study investigating an approach to ranking these properties according to their propensity to speed up or slow down resistance evolution would go a long way, in malaria policy, in helping countries decide which ACTs to prioritize for deployment and therapy switches. Finally, this analysis would have to consider whether – under the most favorable surveillance and deployment conditions – adaptive cycling policies (i.e., ones that trigger switching only when treatment failures meet a certain threshold) are superior to pre-planned fixed rotation strategies. It may be the case that early switches in adaptive policies have detrimental long term effects by moving a health system to use the wrong therapy at the wrong time.

### 4.1 Practical implications

The applied purpose of these research questions is to lay the foundation for scenario-specific national-level mitigation strategies for the artemisinin-resistant genotypes that began circulating in Africa last decade. Our previous efforts at designing such strategies for Rwanda [2] and Uganda [19] used information at just six key loci and were not able to accurately model the full range of pathways to piperaquine resistance. This is critical as DHA-PPQ and ASAQ are the next two ACTs that will be considered in most national contexts as AL use is reduced or phased out. Clinical trials from SE Asia suggest that PPQ deployment may come with high risks of treatment failure [24]. The efficacies shown in these trials, some close to 25%, may represent a worst-case scenario for DHA-PPQ use or may be dependent on certain immunological or parasitological features specific to SE Asian malaria settings. Deployment of DHA-PPQ in Africa is not guaranteed to follow the same pattern seen in SE Asia, but it should nevertheless be accompanied by near real-time molecular surveillance so that PPQ-resistant genotypes can be identified shortly after they emerge. Artesunate-pyronaridine (ASPY) is the fourth ACT that will be considered for deployment. ASPY enjoys high efficacy [42] and a lack of any known pyronaridine-resistant phenotypes, but suffers from high prices and low supply. If ASPY is deployed in an African context and resistant genotypes are found and characterized, the present model will allow for response planning to this emergence event.

A feasible and practical option that is currently being planned by three African National Malaria Control Programs is fast rotation of ACTs [43] – both as preparation for the arrival of resistant genotypes and to slow geographic spread of recently introduced kelch13 variants. The pre-planned rotation periods that are being considered are one, two, and three years, and the shortest of these is likely to see resistance mitigation benefits similar to those of MFT. An early field study indicated that a particular type of stock management will make this rotation feasible in many health-system settings [44] and general social and behavioral studies have indicated that patients, providers, and health systems are ready for a changeover to a rotation or MFT-like approach [44–47]. Evaluating the early emergence and disappearance of resistance alleles in this fast rotation scheme will be critical to understanding which facets of these strategies work better and worse when the cycling period is shortened.

The medium-to-long term option that appears most desirable in Africa is deployment of the triple ACT ALAQ. A Phase 3 clinical trial has completed recruitment, and Phase 2 trials showed efficacy near 100% [48,49] in most regions of SE Asia. Depending on how quickly the approvals, pricing, and manufacturing processes move forward ALAQ may be available for use in some African contexts in 2027. Requiring a fixed dose versus co-blistered combination will add time to this process but improve adherence once triple ACTs are deployed. Cost of triple ACT compared standard AL will be a crucial factor for large-scale continent-wide adoption.

### 4.2 Limitations

This is the first model, to our knowledge, to compare the relative frequencies of recombination through multi-clonal host sampling and interrupted feeding. Further corroboration, through independent modeling efforts or field studies looking at parasite diversity within mosquitoes, will be necessary to build an evidence base showing that one type of recombination event is more common than the other. Genotypic variation in sporozoite populations inside mosquitoes will also need to be modeled as it is known that mosquitoes can inject many sporozoites into a single host during an infectious bite, that these can be of different genotypes, and that this process is important for the overall influence of recombination on parasite evolution [50,51]. As in all large complex individual-based pathogen transmission models, model behavior needs to be continuously validated against field data. As an example, the model’s cost of resistance *c*_*R*_ has been kept deliberately low in comparisons of MFT and cycling approaches because high *c*_*R*_ values in models generally result in MFT having large advantages over cycling policies. However, for practical application and development of national-level strategies, it is more important that *c*_*R*_ estimates are accurate than conservative. Future development in this area will likely combine field data [52] with in vitro data [23] to obtain realistic estimates of fitness cost for drug-resistance mutations in *P. falciparum*.

The major challenge in the evolutionary epidemiology of *P. falciparum* for the near to medium future is building and expanding a genotype-phenotype map for drug-resistant genotypes. Our original drug-by-genotype map, developed for six loci, required substantial approximation as direct treatment-failure data are rarely available for specific genotypes. In the current model version – with 25 relevant loci or CNVs – there are now millions of genotypes and dozens of phenotypes to define (assuming that all of these can emerge and provide some resistance benefit). The sparsity of data for this problem means that substantial uncertainty will remain for the near future on the behaviors, pharmacodynamics, and treatment efficacies on these genotypes.

Finally, additional corroboration will be needed on resistance emergence times for the major ACTs used in Africa and SE Asia. Our model’s mutation rate is currently calibrated against approximate historical data for SE Asia, but not yet adequately to the conditions in the 2010s that led to the emergence of kelch13 variants in Africa under persistent use of artemether-lumefantrine. The decision in SE Asia in the early 2000s to rely on DHA-PPQ and the decision in Africa to rely primarily on AL over the past twenty years could have been an additional major factor (higher drug coverage and lower immunity being two others) influencing earlier emergence of artemisinin in SE Asia and later emergence in Africa.

The global malaria community, together with endemic-country national malaria programs and supporting international institutions, has a major challenge ahead in its efforts to slow the spread of and mitigate the effects of currently circulating artemisinin resistance [53,54]. Novel non-artemisinin therapies and triple combination therapies may be available later this decade. In the meantime, optimal use of current therapies appears to be the only major national-level option available as a specific remedy to spreading drug-resistance. Accurate, robust, and feature-rich mathematical models of malaria transmission will be a key tool in this optimization and treatment strategy design process. A commitment to model evaluation, strategy design, feasibility discussions, and constant iteration of this process will be the key to ensuring that artemisinin-resistant genotypes do not establish themselves continent-wide in Africa by the 2030s.

## Supporting information

S1 Text Full model description and model diagnostics for version 5 of individual-based stochastic simulation of *P. falciparum* transmission and evolution. (PDF)

S2 Text Twenty-one supplementary figures showing sensitivity analyses and robustness checks. (PDF)
