# Supplementary material for: Effects of recombination on multi-drug resistance evolution in Plasmodium falciparum malaria
Source: PLoS Comput Biol. 2025 Aug 25;21(8):e1013401. doi: 10.1371/journal.pcbi.1013401 (PMC12377563; doi:10.1371/journal.pcbi.1013401)
Supplement: S1 Text — (PDF) [file pcbi.1013401.s001.pdf]

Supplementary Materials 1 for

Effects of recombination on multi-drug resistance evolution in *Plasmodium falciparum* malaria
by Tran, Nguyen, Weissman et al (2025)

Table of Contents

1. Basic Model Description for Malaria Simulation Version 5 2
2. Genotype Model 2
3. Multi-clonal infections 5
4. Parasitaemia Model 5
5. Duration of infection 6
6. Host Attractiveness to Mosquitoes 7
6.1. Roulette sampling 7
7. Host Infectiousness to Mosquitoes 9
8. Mosquito Biting Model 10
8.1. Post-recombination mosquito cohort (PRMC). 10
8.2. Total force of infection. 11
8.3. Validation of recombination mechanism using basic measures of linkage disequilibrium. 11
9. Treatment Seeking Model 12
10. Mass Drug Administration Model 12
11. Adaptive Multiple First-line Therapies 13
12. Pharmacokinetics 14
13. Pharmacodynamics and Treatment Failure Model 14
13.1. Example calibration of piperaquine-resistant alleles in pfcrt gene 17
13.2. Calibrate EC50 of Kelch13 alleles 18
14. Host Age Structure 18
15. All-cause mortality and Malaria Mortality by Age 18
16. Probability that an Infectious Bite Causes an infection in a Human 18
17. Model of Immune Acquisition and Immune Waning 19
18. Immune-mediated Symptoms Model 19

1 Basic Model Description for Malaria Simulation Version 5

The simulation model described here is called version 5 and was developed on top of version 3.3 whose most recent publications and descriptions are Li et al [1] and Nguyen et al [2]. Version 4 is the spatial version [3–6] which was built on top of version 3.3. The spatial structure from version 4 is not included in version 5.

First, the genotype structure was upgraded to include all 14 chromosomes explicitly to be able to include *Plasmodium falciparum* resistance markers and their clinical phenotypes (or approximations of these clinical phenotypes by in vitro phenotypes). The reason for this change was the increased number of recently identified drug-resistance markers in *P. falciparum*, especially to piperaquine [7–10]. A major technical upgrade to the codebase was necessary here as version 3.3 used a traditional population-genetic recombination table (size $2^m \times 2^m \times 2^m$) where m is the number of loci — this becomes computationally challenging once $m \geq 10$. Version 5 of the model includes 23 loci with known single amino-acid changes associated with drug-resistance and two copy-number variations for the *pfmdr1* gene on chromosome 5 and the *plasmepsin-2,3* genes on chromosome 14.

The second new feature, added to facilitate handling a large number of loci, was a mosquito population (or cohort) that explicitly bites individuals both to sample from infected individuals and to inoculate new individuals (infected and uninfected) with malaria. Recombination now takes place as an individual event in a mosquito that has just sampled parasites from humans during a blood meal. Mosquitoes may bite two different hosts during a feed – this is called interrupted feeding. The mosquito cohort, its history, and its infecting parasites are all tracked for 11 days so that infectious bites occurring today are from mosquitoes who sampled parasites 11 days ago. The mosquito cohort size is 500 mosquitoes, which was chosen to allow for rare genotypes circulating at frequencies between .001 to .01 to be sampled by mosquitoes in the simulation. This cohort is not intended to represent all mosquitoes with all parasites they would be carrying. Rather, it is meant to be a cohort that holds a representative sample of the genotypes circulating in the human population. Tests were run with cohort sizes of 100, 300, and 500 and no substantial differences in simulation speed or evolutionary outcomes were seen for these different cohort sizes.

The remaining core components of the model are largely unchanged. The model can be viewed as a population of individual agents (humans) that are updated stochastically using a daily time-step via an asynchronous event management system. Each person in the population has basic attributes and exhibits certain behaviors that impact malaria infection such as (*i*) age and mosquito attractiveness – assigned once at birth or at the beginning of the simulation, or (*ii*) a decision to seek treatment and take a particular antimalarial – randomly drawn each time independently after a malaria fever event for each individual, or (*iii*) the number and genotype of different malaria clones that the person carries – which is dependent on recent biting history and recent treatment history.

2 Genotype Model

The new model stores genotype information for each within-host clonal parasite population via a string structured like so

```
||||NYD1||KTHFIMG|||||FNMYRIPRPCA|1
```

with the 13 vertical bars representing chromosome separators and the individual string characters corresponding to amino-acids or other genetic features such as gene copy number. The string structure maps to an integer key in a genotype database and the string itself is used for filtering and parsing output. In the notation above, as an

example, the `NYD1` characters appearing after the four vertical bars correspond to a particular genotype of the *pfmdr1* gene which sits on *P. falciparum* chromosome 5. This genotype is `N86Y, Y184F, D1246Y` which is the wild-type genotype of *pfmdr1*, with the "1" indicating that a single copy of *pfmdr1* is present on chromosome 5. The mutations `86Y`, `184F`, and `1246Y` correspond to alleles whose drug resistance properties have been previously described; details in section 13.

In addition, the simulation has built in functionality to handle multiple genes on the same chromosome, through a data structure based on this string configuration

```
||||NYD1,JXZ||KTHFIMG|||||FNMYRIPRPC|1
```

where chromosome 5 now has a second gene, with three relevant loci, coded into the simulation's genotype database. This is done to allow for intergenic recombination rates on the same chromosome to be specified but is also not used in the current version as no two major resistance-associated genes sit on the same *P. falciparum* chromosome. Table A below described all loci used in version 5 of the simulation.

Chromosome	Name	Locus	Wild-type Amino Acid	Mutant Amino Acid	Mutant phenotype
5	*pfmdr1*	*86*	*N*	*Y*	*amodiaquine resistance; increased lumefantrine sensitivity*
		184	*Y*	*F*	*lumefantrine resistance; increased amodiaquine Sensitivity*
		1246	*D*	*Y*	*reduced susceptibility to lumefantrine (NYD/NFD) decreased sensitivity to chloroquine and amodiaquine (YYY)*
		copy number of this gene	single copy	two or more copies	*mefloquine resistance*
7	*pfcrt*	*76*	*K*	*T*	*chloroquine and amodiaquine resistance; increased lumefantrine sensitivity*
		93	*T*	*S*	*piperaquine resistance*
		97	*H*	*Y*	*piperaquine resistance*
		145	*F*	*I*	*piperaquine resistance*
		218	*I*	*F*	*piperaquine resistance*
		343	*M*	*L*	*piperaquine resistance*
		353	*G*	*V*	*piperaquine resistance*
13	*pfke1ch13*	*446*	*F*	*I*	*artemisinin resistance*
		458	*N*	*Y*	*artemisinin resistance*
		469	*C*	*Y*	*artemisinin resistance*
		476	*M*	*I*	*artemisinin resistance*
		493	*Y*	*H*	*artemisinin resistance*
		539	*R*	*T*	*artemisinin resistance*
		543	*I*	*T*	*artemisinin resistance*
		553	*P*	*L*	*artemisinin resistance*
		561	*R*	*H*	*artemisinin resistance*
		574	*P*	*L*	*artemisinin resistance*
		580	*C*	*Y*	*artemisinin resistance*
		622	*R*	*I*	*artemisinin resistance*
		675	*A*	*V*	*artemisinin resistance*
14	*pfpm-2,3*	copy number of this gene	single copy	two or more copies	*piperaquine resistance*

Table A: All loci and copy number variants used in version 5 of the simulation.

A second string is used as a mask to allow or disable mutation at particular loci, with 0 and 1 corresponding to disable and allow, respectively, like so

```
||||1111||1111111|||||000000000000|1
```

This feature is used for simulation burn-in (if we know for example that *pfkelch13* mutations have not yet appeared in a region) or for specific sub-analyses comparing genotypes or calibrating genotype behavior. For the simulation analysis in the main paper, these masks were used to allow mutation or copy-number variation at 10 positions; see section 2.1 of the main paper.

With this new genotype structure, the model has capability to encode new resistance markers in the future as they are discovered and confirmed. The challenge is that the model also requires the phenotype information for all parasites (across all genotypes) carrying any new allele introduced into the system.

As in all population-level malaria simulations [11] mutation occurs within-host and assumes that the new mutant genotype replaces the resident genotype in a very short period after mutation occurs (i.e. during the course of treatment).

3 Multi-clonal infections

Each host can be infected with multiple parasite populations of different genotypes (or identical genotypes); these parasite populations are sometimes described as 'clones' or 'clonal populations' and they exist (in our simulation) only at the erythrocytic stage of the parasite life cycle. Each parasite clone has its own parasite density. To describe the density of each clone, let c_i be the number of parasite clonal populations in host *i*, and let *j* be the index (from 1 to c_i) of each clone in host *i*. Let D_i denote the total density of transmissible parasites (i.e. stage 5 gametocytes, but these are not modelled explicitly) in host *i*. Each parasite clonal population *j* can be any genotype described in section 2. The mathematical notation for parasite density D_i in host *i* is:

$$D_i = \sum_{j=1}^{c_i} \delta_{j,i}\gamma_{j,i} \tag{1}$$

In equation (1) above, we use $\delta_{j,i}$ to describe the asexual parasite density (stored as a per µl quantity in the simulation) of clone j in host i, and we use $\gamma_{j,i}$ as a binary variable to denote the presence or absence of gametocytes for clone *j* in host *i*.

The binary γ-value is set to zero for the first four (children) or six (adults) days of an infection and then fixed at one for the remainder of the infection. Gametocytaemia is not modelled explicitly. Transmissibility of an infection to a mosquito is based on the relationship between asexual parasite density (total, across all clones) and the probability that at least one functional gametocyte is taken up during a mosquito bite. This probability is shown in section 7 and is derived from Ross et al [8], with an alternate description provided in Nguyen et al [12] (see supplementary materials, pp.6-7).

4 Parasitaemia Model

Parasitaemia is tracked independently for each clone. In general, the parasite density of a person can be classified into 9 different levels, or thresholds, or types which are described below:

Variable name	Parasites per µl	Usage in simulation
log_parasite_density_cured	0.00002 per µl<br><br>(value = -4.699)	When a person is consider cured from a particular parasite infection. This corresponds to 100 total parasites for this particular clone, and when the parasite population falls below this level, it is simply set to zero.
log_parasite_density_from_liver	0.01 per µl<br>(value = -2.0)	This is the number of parasites in the blood immediately after the first group of merozoites bursts from the liver into the blood stream. This corresponds to about 55,000 total parasites.
log_parasite_density_asymptomatic	1,000 per µl<br>(value = 3.0)	Approximate number of parasites per µl for an asymptomatic infection. For an individual who is bitten but does *not* progress to symptoms, a log-parasitaemia y is drawn from a normal distribution with mean 3.0 and standard deviation 0.5, and this person is assigned a starting point of 10^y total parasites in the blood. Afterwards, this parasitaemia declines slowly due to immune clearance.
log_parasite_density_clinical_from	2,000 per µl<br>(value = 3.301)	Bottom boundary for number of parasites for a person experiencing their first day of fever after an infectious mosquito bite.
log_parasite_density_clinical_to	200,000 per µl<br>(value = 5.301)	Upper boundary for number of parasites for a person experiencing their first day of fever after an infectious mosquito bite. For a febrile patient, the parasitaemia at fever is drawn uniformly on [3.3, 5.3] with a resulting parasitaemia between $10^{3.3}$ and $10^{5.3}$.
log_parasite_density_detectable	10 per µl<br>(value = 1.0)	Threshold to determine if a person still has parasites after treatment on day 28. This is a lower threshold than below because microscopy detection limit is lower in a research setting.
log_parasite_density_detectable_pfpr	50 per µl<br>(value = 1.699)	Threshold to determine if a person has parasites and is counted as 'parasite positive' in a PfPR survey. This is based on a typical microscopy threshold that is achievable in a field setting.

Table B: Parasite log-density thresholds and levels used in the model. In the middle columns, the per µl value is also shown on a $\log_{10}$ scale.

5 Duration of infection

For an asymptomatic and untreated infection, the duration of infection varies among individuals depending on that person's immune state. For an infectious bite that leads to an asymptomatic infection or a treatment failure that results in persistent parasitaemia, hosts are transferred into an 'asymptomatic state' and their parasitaemia is set to a randomly drawn value with a mean of 1000 parasites per µl of blood (normal distribution with mean 3 and standard deviation = 0.5).

As in our previous publication, we parameterize the duration of infection to malaria therapy data. According to Maire et al [13] and Eyles and Young [14], mean durations of infection were 169, 121 and 222 days for groups of patients untreated or occasionally treated with quinine during clinical episodes. In addition, the World Health Organization's Garki Project Report [15] indicates an asymptomatic duration between 55 and 500 days and this report shows that older individuals have shorter infection durations due to the development higher immunity.

Using these data as guides for the minimum and maximum length of an asymptomatic infection, the duration of infection in the model is set to have a minimum of 60 days and a maximum of 300 days, for individuals with full immunity and individuals with no immunity, respectively. Note that in our model individuals with naïve immunity will acquire immunity during infection so those individuals will not have 300-day long infections. The expected duration of an asymptomatic infection for an individual starting with no immunity is 281 days for a 1-year old child and 197 days for an 20-year old adult.

Immune system clearance of parasites, for each clone, during the asymptomatic phase occurs according to the following discrete growth model that is evaluated every $K = 7$ days

$$D_{R,t+K} = (1 - c_R)(0.9426 \cdot (1 - M_t) + 0.7442 \cdot M_t)^K \cdot D_{R,t}$$

where D_R is the parasitaemia of clone R and M_t is the immune level (between 0 and 1) at time t. For genotypes with a single mutation the fitness cost above is set to $c_R = 0.0005$ which will result in an approximate 17% annual fitness cost for genotypes carrying one mutation. For a genotype with m mutations, the fitness cost is set to $(1 - c_R)^m$. For some mutations where *in vitro* fitness costs have been measured extensively, we make exceptions, here based on Small-Saunders et al [10], and assign them specific c_R values. For *pfcrt* T93S, we have $c_{R,93S} = 0.0000314$. For *pfcrt* F145I, we have $c_{R,145I} = 0.00102$. For *pfcrt* I218F, we have $c_{R,218F} = 0.000453$. These three values average to $c_R = 0.0005$ and have relative differences according *in vitro* cost-of-resistance measurements.

6 Host Attractiveness to Mosquitoes

Mosquitoes find and select humans for blood meals based on a range of factors including body heat, presence/detection of carbon dioxide, or odor and as a result each person in the simulation is assigned their own 'attractiveness' value to mosquitoes. This attractiveness is implemented as relative biting rate, and each host is randomly assigned a biting rate from a truncated Gamma distribution with mean 5.0 and standard deviation 10.0 (truncated at 0.35 on the left and 35.0 on the right) allowing for a maximum 100-fold difference between biting attractiveness between any two individuals. To improve the biting algorithm in the model, a 'roulette sampling' system was developed which can rapidly and proportionally sample from one million individuals according to their biting attractiveness. This is described below.

6.1 Roulette sampling

In the update from model version 3.3 to model version 5.0, we switch from multinomial sampling to roulette sampling because every individual now has a relative biting rate that is drawn from a Gamma distribution instead of being grouped into 100 discrete groups as in the previous version. Below is the pseudocode of the roulette sampling implementation.

```
function roulette_sampling(n_sample,distribution_list,object_list):
    samples = list(n_sample)
    random_list = list(n_sample)
    sum_distribution = sum(distribution_list)
    for index in random_list:
        random_list[index] = uniform_random(0,sum_distribution)

    random_index = 0
    sum_weight = 0
    for index in distribution_list:
        sum_weight += distribution_list[index]
        while random_index < n_sample and random_list[random_index] < sum_weight:
            samples[random_index] = object_list[index]
            random_index++
        if random_index == n_sample:
            break
    return samples
```

The following example describes how the roulette sampling works in the model. Assume we have a list of six people (this is everyone in the population in this example) and each person has a relative biting rate. We want to select four people from this list based on their biting rate (i.e. attractiveness to mosquitoes). In this selection scheme, selection is done with replacement which means that one individual can be selected and bitten multiple times. A person who has a higher relative biting rate has a higher chance of being selected. The list below contains everyone in the population and it does not need to be shuffled or sorted before beginning the selection algorithm.

Person index i	1	2	3	4	5	6
Biting rate	0.1	0.4	0.9	0.2	0.2	0.5

First, we compute the sum of all the relative biting rates of six persons, which is 2.3. We will use this to perform random uniform draws on the interval [0, 2.3]. We then create a random list with length four (number of bites, or number of people sampled to be bitten) and each list member is a uniform random draw from 0.0 to 2.3, in ascending order. This list must be sorted.

Uniformly Drawn Random Numbers (“boundary markers” on [0, 2.3])	Bitten Index j	0	1	2	3
	Value	0.23	0.575	0.828	1.495

Next, we loop through all individuals. In this loop, we keep track of all “biting rate sums”. In other words, the relevant value in each step of the loop is the sum of all biting rates from person 1 to person i. If the extension of one “biting rate sum” at loop step i to the next “biting rate sum” at loop step $i + 1$ crosses a boundary marker from the sorted list of boundary markers above, person i is selected to be bitten. If k boundary markers are crossed at this step, person i is bitten k times. Once the desired number of bites has been reached, the loop stops.

This process has a complexity of $O(n + m)$ where n is number of individuals and m is number of bites. The running time to sample 2000 individuals out of one million individuals, once per day for 10,000 days, takes around 30-40 seconds.

Random Value (uniformly drawn "boundary marker")	Sum weight	Steps	Selected person
`0.23`	`0.1`	`0.1 < 0.23`:<br>- move to person `2`<br>- bitten index *j* is unchanged (`0`)<br>- add weight of person `2` to sum weight (`0.1 + 0.4 = 0.5`)	
`0.23`	`0.5`	`0.5 > 0.23:`<br>- add person `2` to the "bitten" list<br>- increase bitten index *j* to 1<br>`0.5 < 0.575:`<br>- move to person `3`<br>- bitten index *j* is unchanged (`1`)<br>- add weight of person `3` to sum weight (`0.5 + 0.9 = 1.4`)	`2`
`0.575`	`1.4`	`1.4 > 0.575:`<br>- add person `3` to "bitten" list<br>- increase bitten index *j* to 2<br>`1.4 > 0.828:`<br>- add person 3 to "bitten" list<br>- increase bitten index *j* to `3`<br>`1.4 < 1.495:`<br>- move to person `4`<br>- bitten index *j* is unchanged (`3`)<br>- add weight of person `4` to sum weight (`1.4 + 0.2 = 1.6`)	`2,3,3`
`1.495`	`1.6`	`1.6 > 0.828:`<br>- add person `4` to "bitten" list<br>- increase bitten index *j* to `4`	`2,3,3,4`
		Loop finishes and exits due to random index equaling `4`	

Table C: Roulette sampling explained step-by-step.

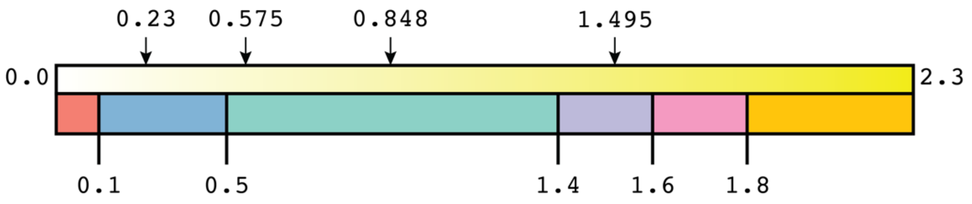

Figure A: Illustration of roulette sampling. The gradient bar on top shows the full range of uniform draws with the four "boundary markers" shown as the four random variates that were drawn. Color blocks at bottom correspond to individuals that have different biting rates (length of block is proportional to the biting rate). Every arrow pointing inside a particular 'person block' corresponds to one bit on that person.

The final list of selected people (i.e. people to be bitten) will be:

Selected individuals to be bitten	2	3	3	4
Individual's Biting rate	0.4	0.9	0.9	0.2

7 Host Infectiousness to Mosquitoes

When a mosquito bites an infected person, there is a chance that the mosquito will take up gametocytes and become infected. A function from asexual parasite density to probability of successful infection was developed by Ross et al [8] and re-described in our previous model (version 3.3 – see Nguyen et al [12] pp.6-7 of supplement). This same function, shown below, is used in version 5 of our model.

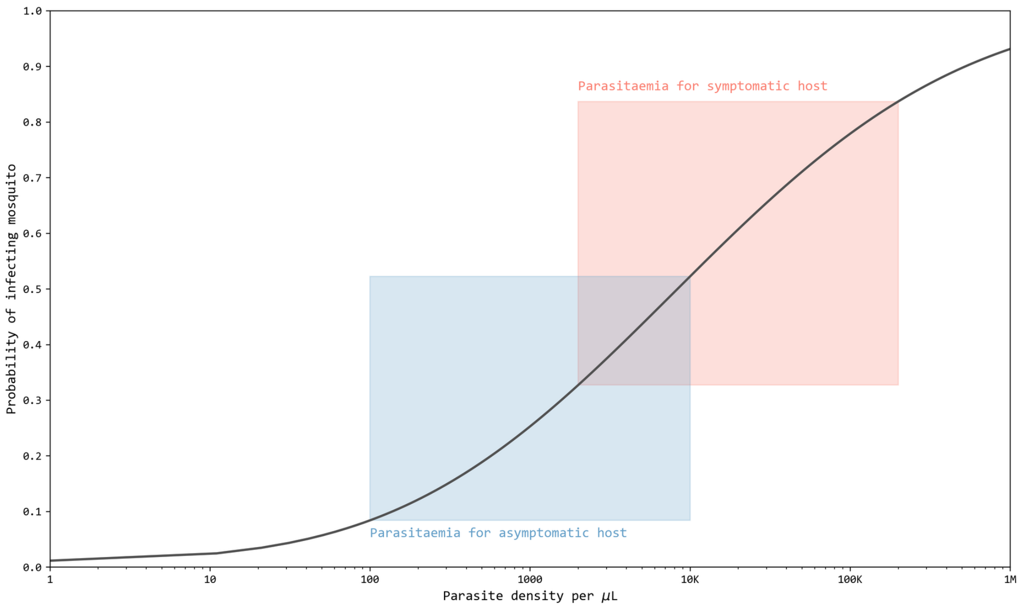

Figure B: Relationship showing probability of infecting a mosquito during a single feed as a function of asexual parasite density (which is assumed to be proportional to gametocyte density during most stages of an infection).

Ranges for parasitaemia and infection probability for asymptomatic and symptomatic hosts are shown in blue and red (respectively).

8 Mosquito Biting Model

In sections 2 through 5, we defined the *P. falciparum* genotypes used in the simulation, the multi-clonal structure of infection, and the parasitaemia level for each genotype including how it is set, increased, and decreased. In sections 6 and 7 the level of biting on individuals and whether this biting leads to infected mosquitoes is described. Here in section 8, we describe a major new model component describing how mosquitoes take parasites (sometimes multiple parasites with different genotypes) up from infected hosts and how these parasites recombine within the mosquito.

8.1 Post-recombination mosquito cohort (PRMC)

First, each day, we create a new 500-mosquito cohort of infected mosquitoes that is meant to hold a representative sample of currently circulating falciparum genotypes. Cohort history is tracked for 11 days so that human hosts infected today are bitten by mosquitoes that fed 11 days ago. A daily cohort is created by simulating 500 bites on human hosts – sampled via roulette sampling according to the product of biting attractiveness and host infectiousness to mosquitoes. These 500 bites on human hosts may be interrupted feeds on pairs of hosts or single feeds on single hosts. These bites may take up multiple gametocytes with different genotypes. Random union of gametes followed by random segregation of chromosomes (i.e. traditional recombination for sexually reproducing organisms) occurs in the model for the gametocytes the mosquito has taken up. No recombination occurs within chromosomes in this version. Then, one parasite haplotype post-recombination is assigned to the subsequent population of ookinete, oocysts, and sporozoites that will be able to infect new human hosts 11 days later.

When a mosquito takes a blood meal, it will take either a full blood meal on one host or be interrupted by the human reaction to the bite and it may then complete its meal on a second host. This interrupted feeding probability (i.e. the probability that a fed mosquito has human blood from two distinct humans) has been measured to be as high as 20% in some contexts [16–25], and we vary this probability in our current analysis. The probability of mosquitoes taking interrupted feeds is used in the biting model whose details are described in below in the diagram below.

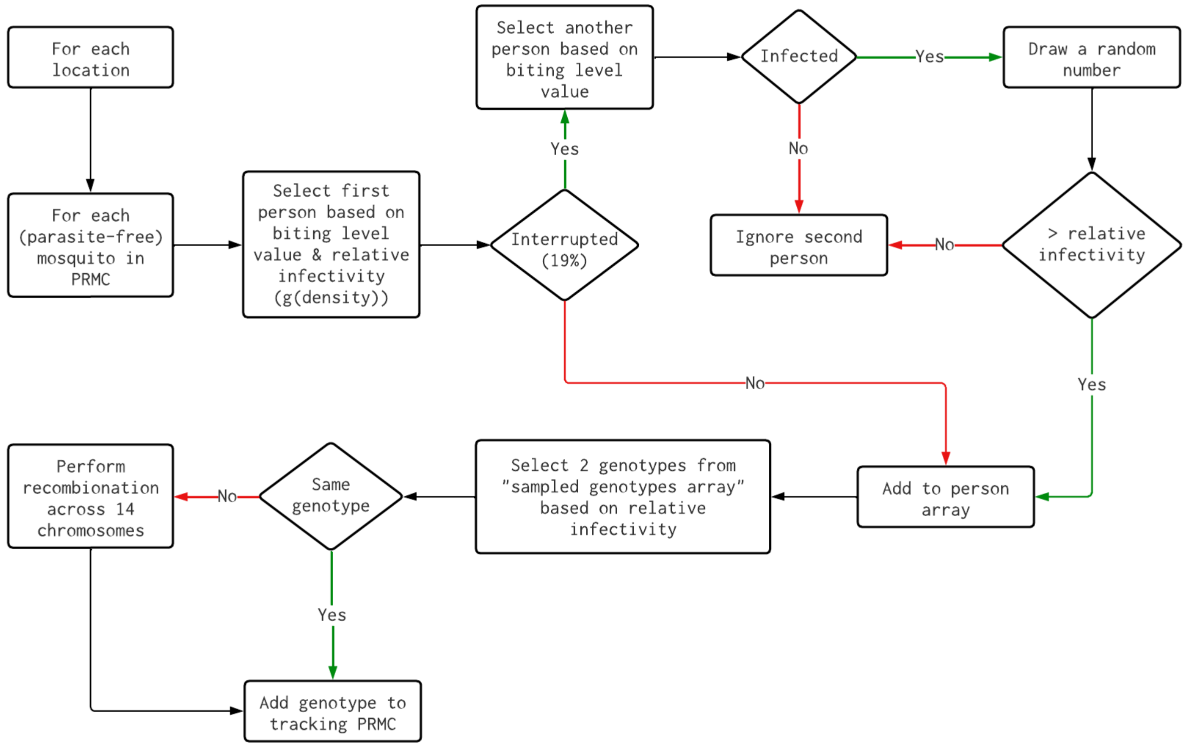

Figure C: The flow diagram of how each mosquito in the cohort (the PRMC) is assigned a particular genotype after recombination has taken place. Selecting genotypes from the 'sampled genotypes array' is done with replacement.

8.2 Total force of infection

In the previous model version 3.3, the force of infection (FOI) of each genotype (accounting for recombination with a mating-table approach) was used to compute the number of bites of that genotype. In the current version 5, the FOI is generated based on the total number of parasites in the population across all genotypes. The equation below defines the total force of infection Λ_t at time *t* in the model.

$$\Lambda_t = \beta \sum_{all\ hosts\ i} g(D_i) \cdot b_i \qquad (2)$$

where (*i*) the overall transmission parameter β is calibrated in all model runs to achieve a particular malaria prevalence level, (*ii*) the infectivity-to-mosquitoes function *g*, which depends on parasite density D_i, is described in section 7, and (*iii*) the biting levels or human biting attractiveness parameters b_i are described in section 6. The number of new infectious bites on a particular day *t* in a particular location is sampled from a Poisson distribution with mean set to Λ_{t-11}.

When an individual is bitten and infected, a new *P. falciparum* genotype is chosen randomly (uniformly) from the 500 mosquitoes in the PRMC. An 11-day history of the PRMC is tracked, so today's infections will have their genotypes sampled from the PRMC created 11 days ago.

8.3 Validation of recombination mechanism using basic measures of linkage disequilibrium

Linkage disequilibrium (LD) is the non-random association of alleles at different loci in a population. For alleles *A* and *a* at one locus and alleles *B* and *b* at a second locus, we use the following simplified equation to describe the LD between *A* and *B:*

$$LD = f_{AB} - f_A f_B$$

without any normalization typically used in LD equations. The parameter *f* simply denotes the allele frequency of A or B, or the genotype frequency of AB. We do this simply to verify that LD approaches zero in situations with no selection, no mutation, and no population structure. We simulate a population with two parasite genotypes (K76 C580 and 76T 580Y) at equal 0.50 genotype frequencies as the starting point with no drug treatment, no cost of resistance for mutant alleles, and no mutation allowed in these runs. In all situations tested (*N* = 1600 simulations in total) the LD between these loci approached zero and the four genotype frequencies reach equilibrium frequencies of 0.25.

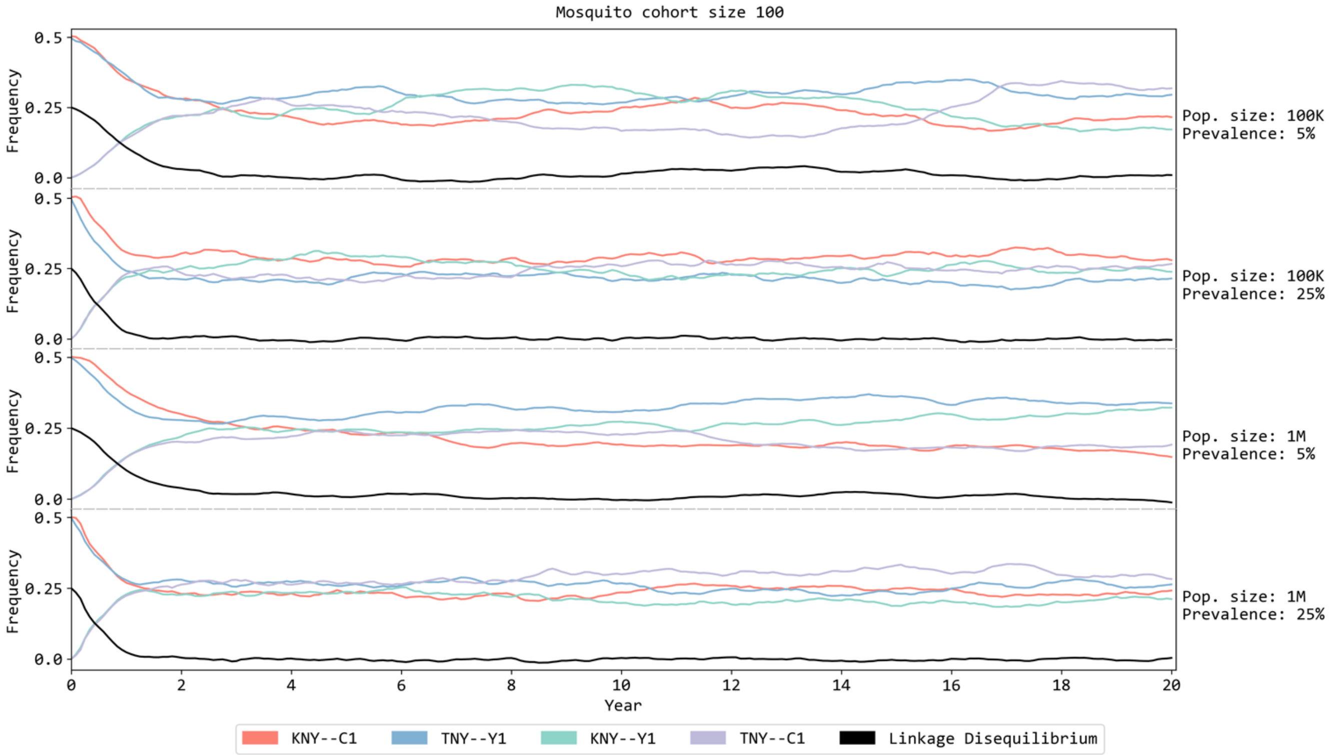

Figure D: The linkage disequilibrium (LD) output of the model with two population sizes in two prevalence settings. The *x*-axis indicates the number of years run while *y*-axis shows the frequency of the genotypes and LD. In this setting, two initial genotypes (KNY—C1 in pink color and TNY—Y1 in cyan color) are seeded into the population (i.e. the two distinct genotypes are K—C and T—Y). No treatment is applied in this setting and the mosquito interrupted feeding rate is set to 99% to accelerate recombination. The expected result is four genotypes distributed with approximately 0.25 genotype frequency each and with LD approaching or close to zero.

9 Treatment Seeking Model

In our model, the probability that an individual seeks treatment is called treatment coverage and this probability is different for individuals aged under 5 and over 5. One treatment coverage is set when running the model in the burn-in phase (while the model approaches equilibrium) and another treatment coverage is set when treatment strategies begin to be evaluated. Increases in treatment coverage can be modeled during the treatment strategy period. Individuals can choose to seek treatment in the public or private market, and data for this are typically obtained from Demographic Health Surveys (DHS). Depending on the health-care setting individuals can receive the recommended first-line therapy or one of a number of therapies/drug sold in private clinics or pharmacies (again, parameterized using data from DHS surveys).

10 Mass Drug Administration Model

In malaria interventions, mass drug administration (MDA) is sometimes deployed to give treatment to everyone – sick or healthy, infected or uninfected – in a particular geographical area. Since everyone is receiving treatment, malaria transmission among individuals is reduced and lowers the number of new infections in the population, sometimes to zero. This strategy is a good option to eliminate malaria in low-endemicity regions (with prevalence < 1.0%) where everyone has good access to treatment, vector control, and routine diagnosis and surveillance. In our model, the number of people that receive treatment during MDA is determined by individuals' variability in participating (i.e. showing up) in the MDA. Each individual is assigned a participation probability drawn from a β-distribution with standard deviation set to 0.3; see Nguyen et al [2]. In the analysis in the present manuscript, MDA scenarios were not evaluated.

11 Adaptive Multiple First-line Therapies

Deployment of multiple first-line therapies (MFT) is a strategy where different ACTs (or in general, different therapies) are distributed simultaneously in a population to provide a variable drug environment for the purpose of slowing the evolutionary/adaptive process of malaria parasites [26]. This strategy has the advantage of both delaying the emergence of resistant parasites and reducing the number of treatment failures, according to both mathematical and agent-based models [12,26].

In version 3.3, MFT is implemented with an equal distribution of all ACTs. In this version, we also introduce an "adaptive MFT" approach in which the distribution of a drug into a population is adjusted based on the number of treatment failures of that drug. Initially, for three therapies, the distribution of each drug is set at 33.3%. After a specified duration (2 years), the fractional distribution of each therapy (denoted here by μ_i) is adjusted for each drug based on its most recent 60-day treatment failure rate F_i:

$$\mu_i = \frac{F_i^{-1}}{F_1^{-1} + F_2^{-1} + F_3^{-1}}$$

where F_i is replaced with 5% if the failure rate is below 5% (all others are then rescaled or renormalized to maintain their relative proportions). The new distributions are put in place after a delay of one year. This adjustment will use drugs in inverse proportion to their failure rates. In other words, if therapy *a* has three times the failure rate of therapy *b*, therapy *a* will be used for one-third as many malaria cases as therapy *b*.

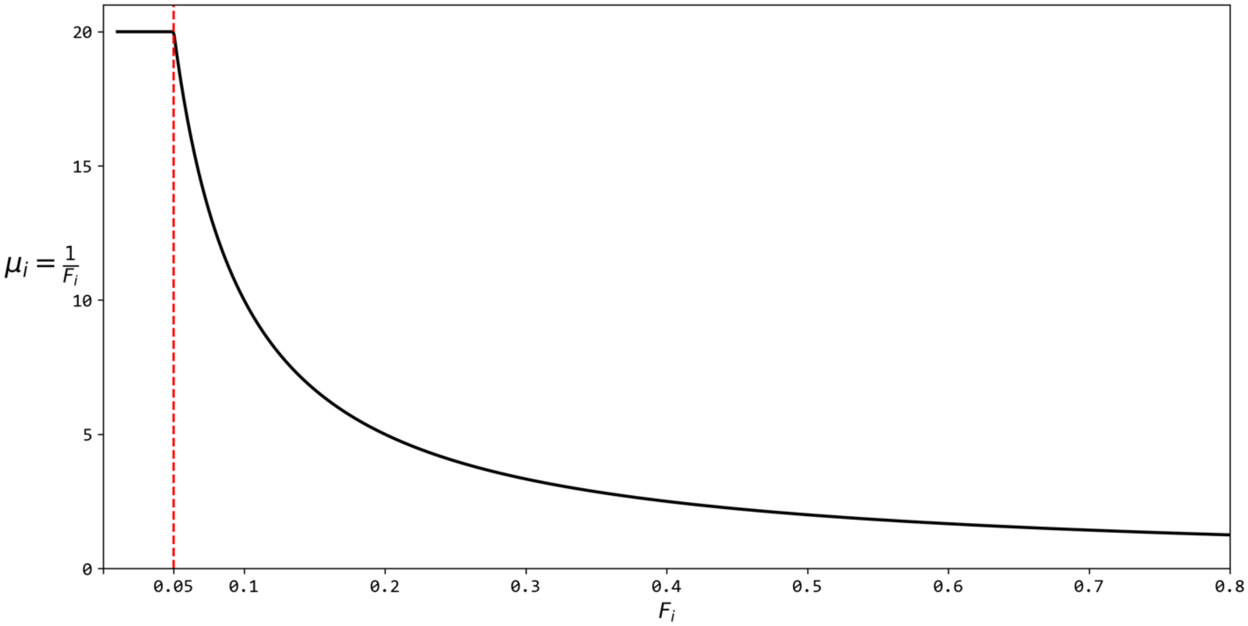

Figure E: The relation between treatment failure of a drug (Fi on x-axis) and its new distribution (μ_i, on y-axis) in adaptive MFT method.

12 Pharmacokinetics

In this version, a single compartment pharmacokinetic model is used, and drug half-life data are the same as in version 3.3 with following values used:

Drug	Half-life (t*)
Artemisinin derivatives	4-11 hours (DHA: 11-12 hours)
Lumefantrine	4.5 days
Amodiaquine	9 days
Piperaquine	28 days
Mefloquine	21 days
Chloroquine	10 days
Ganaplacide (KAF156)	1.6 days

Table D: Half-lives of monotherapies. Source in Nguyen et al [12].

13 Pharmacodynamics and Treatment Failure Model

In Nguyen et al [12], the pharmacokinetics model is a modified version of the standard Michaelis-Menton equation (or Hill equation)

$$P(C) = p_{max} \cdot \left(\frac{C^n}{C^n + EC_{50}^n}\right)$$

with *C* as the drug concentration in the patient's blood (central compartment), and p_{max} as the daily fraction of parasites that are removed (killed) by a high drug concentration drug. The parameter *EC50* is the concentration

level corresponding to 50% killing effect, and n determines how steep the concentration-effect curve is. The parasite density at day t is:

$$p(t) = (1 - P(C)) \cdot p(t-1)$$

Since $EC50$ determines the percent of parasites killed by a certain concentration of drug and since parasites can mutate to a higher-survivorship phenotype to that drug, we associate each genotype with an $EC50$ value for a particular drug. In the previous version of the model (v3.3), there were 64 genotypes in total and the $EC50$ values were computed (approximated) in advance as a lookup table of $EC50$ values associated to genotypes.

In the new version of the model (v5) with the loci described in section 2, the number of possible genotypes has increased to 2^{25} (about 32 million) and the $EC50$ values of new genotypes must now be calculated on the fly to save memory and computation time. This is done in two-step process:

Step 1. When a mutation occurs, a new genotype is formed that differs by one allele (normally) from a previously existing genotype. Considering all the mutant alleles (or derived alleles) and all drugs in the simulation, each mutant-drug combination is assigned an "EC50 shift factor" (parameter *e*) that shifts the EC50 value to the right from EC50 to $e \cdot$ EC50 generating a classic rightward-shifted dose response curve. The parameter *e* is denoted by multiplicative_effect_on_EC50 in the simulation's input file, and it is best written as $e_{\text{art,R561H}}$ to specify the increase in resistance associated with a particular allele (here 561H) when encountering a particular antimalarial compound (here artemisinin).

The mutant-drug combination is simply a genotype-environment combination. An increase in EC50 increases survivorship for a genotype in a particular environment, and the *e*-parameters therefore can be viewed as factors influencing each genotype's Darwinian fitness, although the exact relative fitnesses in these comparisons are not 1.0 and *e*. When multiple mutations occur, the new EC50 is calculated simply as $e_1 \cdot e_2 \cdot e_3 \cdot$ EC50 assuming a level of independence among these mutations' three fitness effects. In other words, as assumption of non-epistasis is made in the initial calibration of the fitness effects across genotypes. We simply have single-locus effects in given drug environments. These single-allele effects are shown in the table below.

Name	Locus	ART	LUM	AMQ	PPQ	MQ	CQ
Pfmdr1	*N86Y*		1.0 (1.25)	1.42			1.25
	Y184F		1.25	1.2			
	1246						
	copy number of this gene		*1.06*			*2.4*	
Pfcrt	*K76T*		1.0 (1.1)	1.0 (1.2)			1.6
	T93S				1.6648		
	H97Y				1.6648		
	F145I				2.2226		
	I218F				1.6648		
	M343L				1.0		
	G353V				1.0		
Pfk13	*F446I*	1.43					
	N458Y	1.69					
	C469Y	1.53					
	M476I	1.53					
	Y493H	1.69					
	R539T	1.53					
	I543T	1.53					
	P553L	1.53					
	R561H	1.69					
	P574L	1.60					
	C580Y	1.60					
	R622I	1.60					
	A675V	1.60					
Pfpm2,3	*copy number of this gene*				1.37		

Table E: Multiplicative effect that each mutant allele has on each drug's efficacy. Number shown in each cell is the *e*-value for that mutation's effect on the EC50 to a particular drug; when no value is shown this means there is no effect (*e* = 1.0). In some cases, a mutation will lower an EC50 value, e.g. when 76T mutates to K76 the EC50 for lumefantrine increases. In these cases, the mutant's EC50 is shown as with an *e*-value of 1.0, and the original *e*-value for the wild-type allele is shown in parentheses.

As an example, consider the following alleles in the *pfcrt* gene (chromosome 7) that influence piperaquine resistance:

amino-acid position in pfcrt:	93	97	145	218	343	353
Allele (wild type)	T	H	F	I	M	G
Allele (mutant)	S	Y	I	F	L	V
Piperaquine Base EC50	0.58					
EC50 factor (wild type)	1.0					
EC50 factor (mutant)	1.66	1.66	2.22	1.66	0.99	0.99

Table F: Multiplicative effects (bottom row) on EC50 of six different mutations in *pfcrt* associated with PPQ resistance.

The EC50 value for piperaquine's action on the wild-type genotype THFIMG is 0.58 × 1.0, the EC50 value for PPQ on the 1-point mutant THIIMG is 0.58 × 2.22, and the EC50 for PPQ on the 2-point mutant TYIIMG is equal to 0.58 × 1.66 × 2.22.

This summarizes the process of approximating EC50 values across all loci in the current simulation when multiple resistance mutations are present.

Step 2. For some genotypes, fitness values (in the form of treatment failure rates) will have been measured in clinical trials or therapeutic efficacy studies (TESs). These measured or approximated genotype-specific treatment failure rates can be seen in Supplement 2 of Nguyen et al [2]. When a genotype-specific approximation for an EC50 or a treatment failure rate is available, this value will override the calculated value in Step 1 above.

In steps 1 and 2 above, it is assumed that base EC50 values exist for the wild-type genotype. These base EC50 values are calculated using well-known treatment efficacies on wild-type parasites. Since the method of updating EC50 values has changed (from v3.3 to v5) due to the additional genotypes to the model, the efficacy of each drug needs to be calibrated again, especially for piperaquine and piperaquine-resistant genotypes in *P. falciparum* chromosome 7. Some of this calibration process is described below.

13.1 Example calibration of piperaquine-resistant alleles in pfcrt gene

According to a number of in vitro studies [7,9,10,27] and in vivo studies [28], six amino acids in *pfcrt* gene are confirmed to confer the resistance to piperaquine: T93S, H97Y, I145F, F218I, M343L and G353V. Four of these alleles had in vivo efficacies measured as part of the TRACII study in SE Asia (during 2015-2018 [28]). The EC50 values for the T93S, H97Y, F145I, and I218F alleles were estimated as follows.

Out of 140 patients that received DHA-PPQ in this study, 119 had their *pfcrt* alleles characterized and 31 had none of the four alleles listed above. The study population as a whole was characterized as 74% with double-copy plasmepsin and the 580Y allele, 17% with single-copy plasmepsin and the 580Y allele, and 9% with single-copy plasmepsin and wild-type C580. A simulated clinical trial population was created with these genotype proportions (74, 17, 9) and EC50 value for the effect of multi-copy plasmepsin on PPQ activity was inferred by matching the simulated efficacy to the measured efficacy on these 31 patients (87.0% efficacy). See top row of Figure F.

For each patient subgroup, stratified by genotype (see Figure 1B in van der Pluijm et al [28]), we re-ran a simulated clinical trial with the above population genotype distribution (74, 17, 9) to match the measured DHA-PPQ efficacies on patients carrying one of the 93S, 97Y, 145I, or 218F alleles. In the trial, patients carrying 93S, 97Y, and 218F had an estimated 28-day treatment efficacy of 50.7% and patients carrying the 145I allele had an estimated treatment efficacy of 39.8%.

The mutations 343L, 353V are currently modeled as having no effect on piperaquine resistance because of the lack of in vivo data.

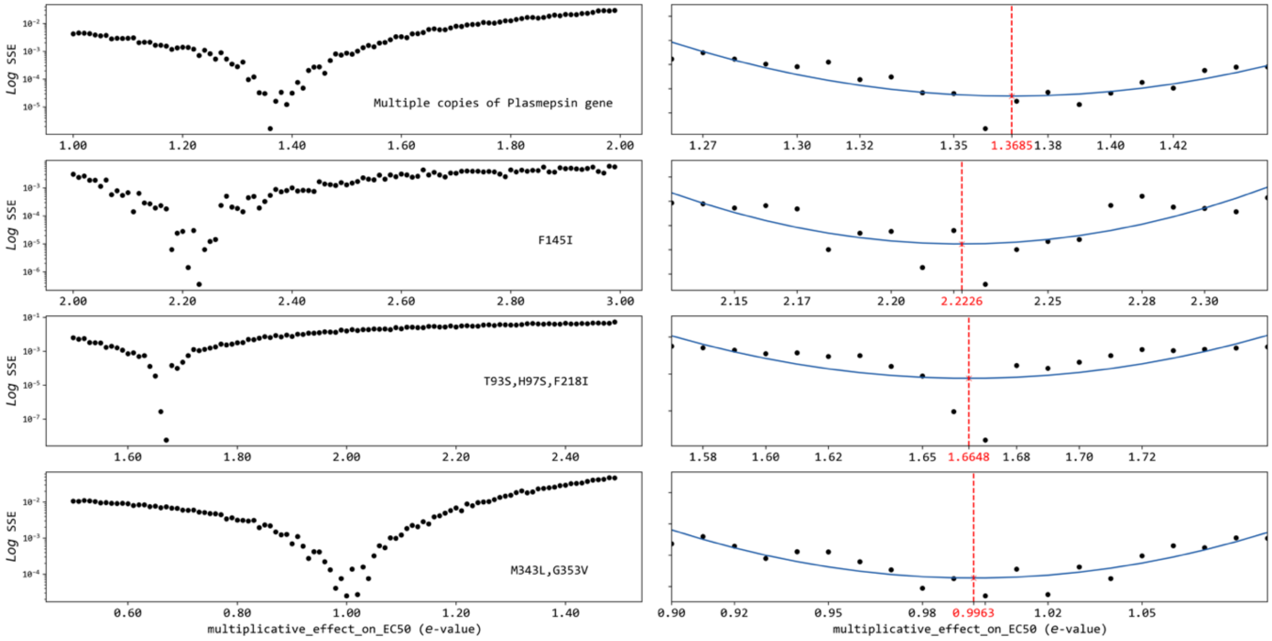

Figure F: Log likelihood fitting the *e*-value to get the DHA-PPQ efficacy of the model to match clinical data. Each panel row presents a likelihood-fit of the *e*-value using sum of squared error and the left panels presents the errors of fittings while the right panels show the optimum *e*-value. The *x*-axis shows the *e*-values of each allele in the *pfcrt* gene or copy-number variation of the plasmepsin gene. The *y*-axis presents the sum of squared error in log10-units. Each black dot shows the sum of squared error between DHA-PPQ efficacy in the simulated data with a specific *e*-value (summed across 10,000 patients) and the clinical data. The blue line indicates the quadratic fitting of 20 data points around the lowest *e*-value on the left panel. The value in red indicates the maximum likelihood *e*-value.

13.2. Calibration of EC50 for kelch13 alleles

To calibrate the EC50 of various *pfkelch13* alleles, we used parasite clearance half-life ($PCT_{1/2}$) estimates from a WWARN meta-analysis [29] to estimate the parasite density after one day with artemisinin monotherapy (assuming the partner drug had little effect during this time). From Figure 4 in this meta-analysis, the mean clearance half-lives of all *pfkelch13* alleles in Table E can be grouped into three groups shown in Table G below.

**Allele**	**Range of $PCT_{1/2}$**	**expected log10-parasite density after 24 hours**
F446I	5.389	2.961
R539T/I543T/P553L/C469Y/M476I	5.87 – 6.43	3.122
P574L/C580Y/A675V	6.61 – 6.71	3.218
N458Y/Y493H/R561H	7.21 – 7.52	3.323

Table G: List of *pfkelch13* alleles, their parasite clearance half-lives ($PCT_{1/2}$), and the calculated mean parasite density 24 hours after presentation based on this $PCT_{1/2}$. It is assumed that mean parasite density at presentation is $10^{4.301}$ or 20,000 parasites per microliter. The parasite clearance half-life is shown in hours.

In our model the mean log-parasite density across 10,000 symptomatic patients with 580Y parasites is $10^{4.301}$ or 20,000 parasites per microliter (at presentation) and after one day of using artemisinin monotherapy the parasite density should be reduced (according to the half-lives above) to a level between $10^{2.9}$ and $10^{3.4}$ (i.e. between 900 and 2200 parasites per microliter). This would be consistent with a parasite clearance half-lives in the range of 5.8 to 7.5 hours for mutant *pfkelch13* genotypes. Our model achieves an approximate version of this behavior (roughly a 12-hour half-life) as it is the 28-day efficacy of 580Y parasites, and not their half-life, that is calibrated to data in our model.

Reduction in PRR (parasite reduction ratios) in the model is done by increasing the EC50 parameter (for artemisinin) which reduces the model-calculated PRR values of artemisinin monotherapy on mutant *pfkelch13* genotypes. We increase artemisinin's EC50 value even though pharmacokinetics of artemisinin are not modeled explicitly; most of the pharmacokinetic and pharmacokinetic model parameters do not have perfect correspondence with published PK/PD data. Variation (among patients) in PRR values is used to calibrate a 28-day efficacy of 25.5% for three days of artemisinin monotherapy on an uncomplicated falciparum infection with a 580Y genotype. This ~25% efficacy was chosen based on genotype-stratified trial data from SE Asia last decade (see p11 in supplement 2 of Nguyen et al [2]). In a model-simulated clinical trial of patients infected with 580Y parasites and treated with three days of artemisinin monotherapy, 23% of patients have a daily PRR > 10 and 16% of patients have a daily PRR > 50, and it is this variation in the model that results in some patients being treated successfully while other patients fail treatment.

For alleles 446I, 539T, and 561H, the calibration is done differently. The mean daily PRR-values (calculated from $PCT_{1/2}$ values in Table G) for these three alleles are 21.9, 15.1, and 9.5, respectively. For these PRRs, the expected log-parasitaemia values after 24 hours are shown in Table G; these values are adjusted up by a factor of 1.149, which is the exact difference between the expected log-parasitaemia at 24H for a 580Y infection based on $PCT_{1/2}$ and the expected log-parasitaemia at 24H for a 580Y infection based a 28-day efficacy of 25%. This adjustment of 1.149 is included in the sum-of-squares calculation in Figure G. The modeled 28-day efficacies of artemisinin monotherapy on these 446I, 539T, and 561H are 39.7%, 30.5%, and 20.3%, respectively.

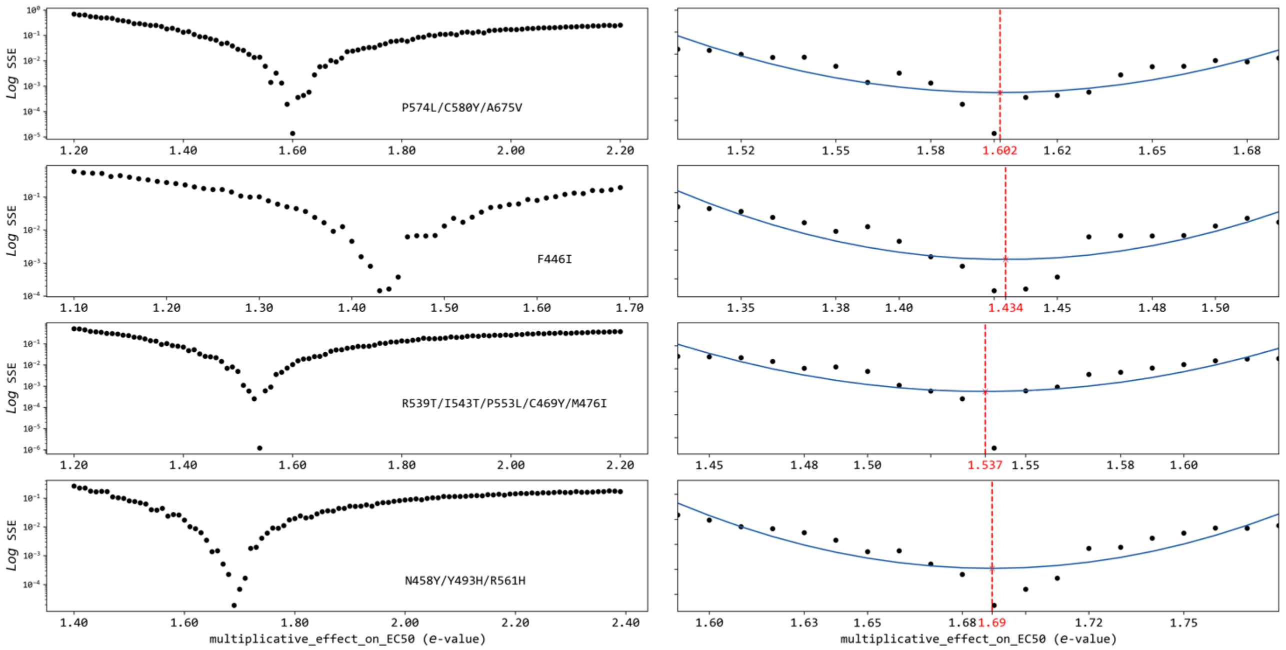

Figure G: Log-likelihood fitting of the *e*-value based on modeled parasite density on day 2, from which a parasite clearance half-life is calculated; likelihood comparison done to match $PC_{1/2}$ values in WWARN meta-analysis [29]. Each panel row presents a likelihood fit of an *e*-value using sum of squared errors; the left panels show all errors while the right panels show a zoomed-in version and the maximum-likelihood *e*-value. The *x*-axis shows the *e*-values of each allele in *pfkelch13* gene that confers resistance to artemisinin. The *y*-axis presents the sum of squared error in log10-units. Each black dot shows the sum of squared errors between parasitaemia on day 2 of the simulation (across 10,000 simulated patients) and the inferred parasite density from clearance half-life in the WWARN meta-analysis. The blue line shows a quadratic fit of 20 data points around the lowest *e*-value on the left panel. The value in red on the *x*-axis indicates the optimal *e*-value that matches parasite density on day 2.

14 Host Age Structure

People with different ages respond to malaria infections differently due to their prior malaria exposure history, current level of immunity, and acquisition rate of immunity. Age is tracked in the model for all individuals. Individuals are born in the model on specific days and are assigned birthdays. Individuals instantiated before burn-in are given random birthdays according to the expected population age structure.

For computational efficiency, the individuals are grouped into 21 age groups, able to be indexed in a particular array structure of the model for faster searching and reporting. In these 21 age groups, individuals younger than 15 are grouped into 15 groups based on one-year age bands. Individuals older than 15 and younger than 65 are grouped into 5 age groups: 15 to 24, 25 to 34, 35 to 44, 45 to 54 and 55 to 64. The last age group is individuals older than 65. The exact percentage of the population in each age group can be found in section 7 in supplement of Nguyen et al paper [12], taken from the Tanzania population report from the Tanzanian National Bureau of Statistics. These age groups are taken as representative, and the generic model setup is not meant to replicate epidemiological conditions of malaria in Tanzania (unless specifically calibrated).

15 All-cause mortality and Malaria Mortality by Age

The model's all-cause and malaria mortality has not changed since the 2015 Nguyen et al paper [12] (section 6 of supplement) and is based on several large but older studies on malaria morbidity, severity, and mortality [30–33]. Briefly, for an untreated symptomatic malaria case, the model's mortality rate is (1) 4.0% for patients 12 months of age or younger, (2) 2.0% for age group 1-4, or 12 months to 59 months of age, (3) 0.4% for age group 5-10, and (4) 0.1% for individuals 11 or older. These mortality rates account for the fact that a certain percentage of untreated malaria cases progress to severe malaria, but our simulation does not model the symptoms progression from uncomplicated to severe. For treatment failures, the age-specific mortality rates are the same as for the untreated cases shown above. For treatments that result in adequate clinical and parasitological response (ACPR) the malaria mortality rate is zero for all ages.

16 Probability that an Infectious Bite Causes an infection in a Human

When an infectious mosquito bites a human, there is a chance that the mosquito successfully infects that person with sporozoites and that the sporozoites develop in the liver and eventually progress to be released into the blood as merozoites. During simulation, only 10% of individuals that are bitten each day are selected to have a successfully established blood-stage infection. This probability is 10% in both versions 3.3 and 5 of the simulation. This probability affects the EIR values reported in the model, as EIR counts all bites by infectious mosquitoes even ones that do not cause established blood-stage infections.

17 Model of Immune Acquisition and Immune Waning

As in Nguyen et al [12] (supplement, section 10) the acquisition of immunity of an individual harboring blood-stage parasites in the bloodstream is defined by the following equation, where $M(t_1)$ represents the a person's current "immunity level" on a scale of zero to one:

$$1 - M(t_2) = (1 - M(t_1))e^{-a_2(a)\cdot(t_2 - t_1)}$$

Above, $a_2(a)$ is the age-dependent rate of immune acquisition (see below), and $t_2 - t_1$ is the time interval (in days) between assessments. In the absence of infection, an individual's immunity wanes over time, as governed by the equation:

$$M(t_2) = M(t_1)e^{-a_1(t_2 - t_1)}$$

where $a_1(t_2 - t_1)$ is the rate of immune decay during the time $t_2 - t_1$. We set the value $a_1 = 0.0025$ corresponding to 90% immune loss after 2.5 years [34]. To account for age-dependent differences in immunity acquisition, we applied two different equations for individuals below/above 10 years of age. For those 10 and under, the daily immune acquisition rate a_2 is:

$$a_2(a) = (a/10)^{\kappa} \times (1.01)^{a} \times 0.00125$$

where the parameter κ controls the curvature of age-specific immune acquisition dynamics. A lower κ leads to similar immune acquisition rates between children and adults (see Figure H). Conversely, higher values of κ result in children experiencing much slower rates of immune acquisition than adults. For individuals over the age of ten, the daily immune acquisition rate is set to

$$a_2(a) = (1.01)^{a-10} \times 0.0013809$$

indicating that "full immunity" or "high immunity" to malaria is acquired by adults or older children in about one or two years. The parameter κ is calibrated (see section below) to match field data on age-specific malaria symptoms presentation.

18 Immune-mediated Symptoms Model

The probability of progressing to symptoms, denoted as P_{clin}, is formulated as a function of the immunity level *M* according to the equation:

$$P_{clin} = \frac{0.99}{1 + (M/M_{mid})^z}$$

Here, the parameter z characterizes the relationship between immunity level and the likelihood of symptoms development. To explore its impact on clinical malaria patterns, z is varied between 2.0 and 8.0. We set M_{mid} to 0.4, consistent with the approach outlined in the Filipe et al paper [34]. Figure I shows probability of symptoms as a function of immune level *M* for a range of *z* values.

To calibrate κ and *z* we need data on age-specific incidence in different transmission settings. These data were assembled for our previous calibration (see supplement section 11 in Nguyen et al [12]) and they provide, for each transmission setting, a ratio of incidence in 2-year-olds to incidence in 10-year-olds. These ratios are the orange dots in Figure J. The model-to-data comparisons in this figure show that *z*-values in the range 4.6 to 6.4, and κ-values between 0.1 and 0.4 give the best fits of model to data. The fine-scale heat map in Figure K shows that a basic mean-squared error optimization gives z = 5.4 and κ = 0.3 as the best-fit values. These values are used in the simulation.

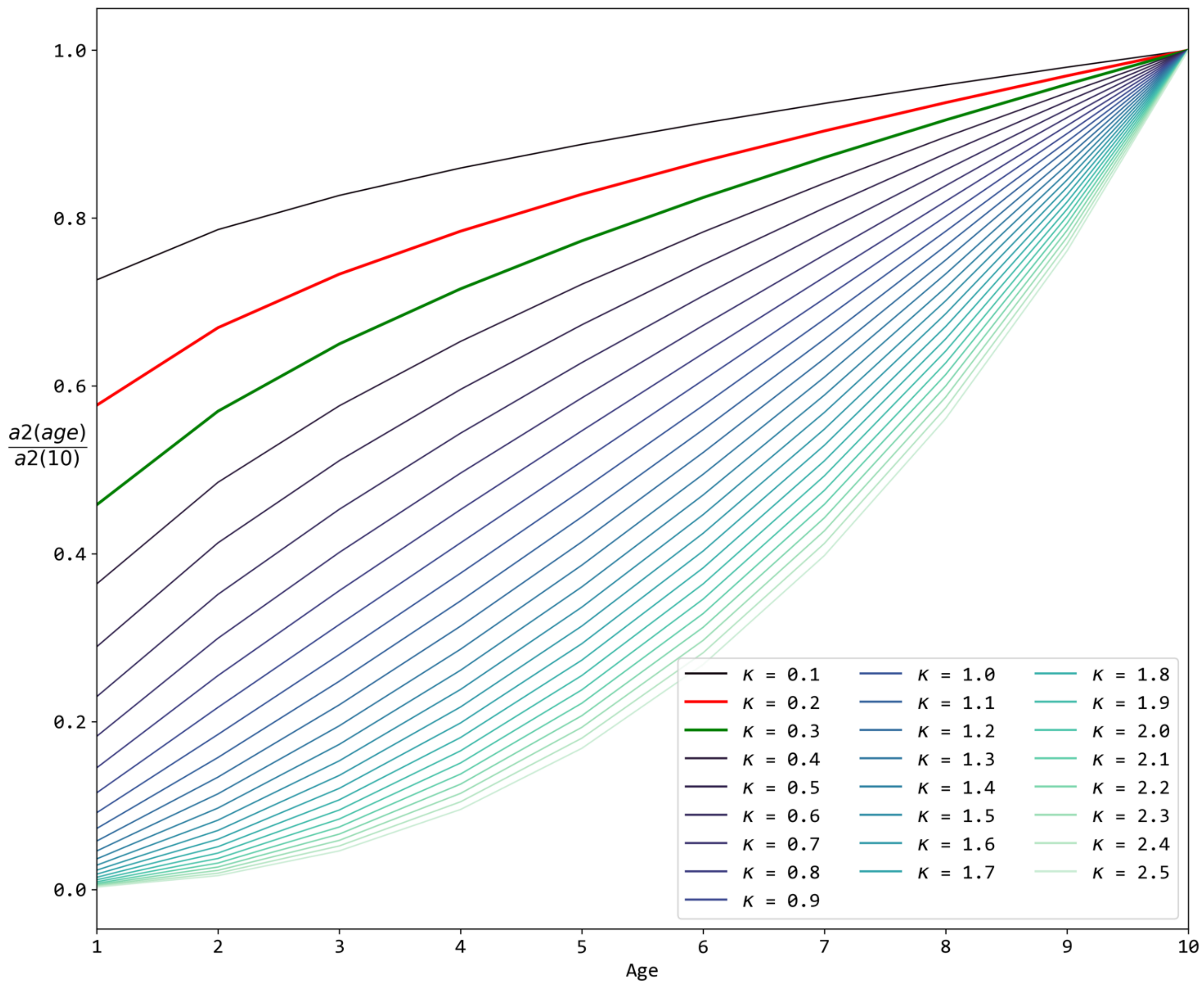

Figure H: The y-axis shows a child's relative immune acquisition rate when compared to a ten-year old, based on Nguyen et al [12]. Different lines are drawn for different values of the κ parameter ranging from 0.1 to 2.5. The red (κ=0.2, for the no treatment scenarios in Figure K) and green (κ=0.3, for the 50% treatment coverage scenarios in the remaining figures) lines denote the selected κ-values after calibration. Higher κ means that children in lower age groups will have slow immune acquisition rates until they reach the normal rate calibrated for ten-year-olds.

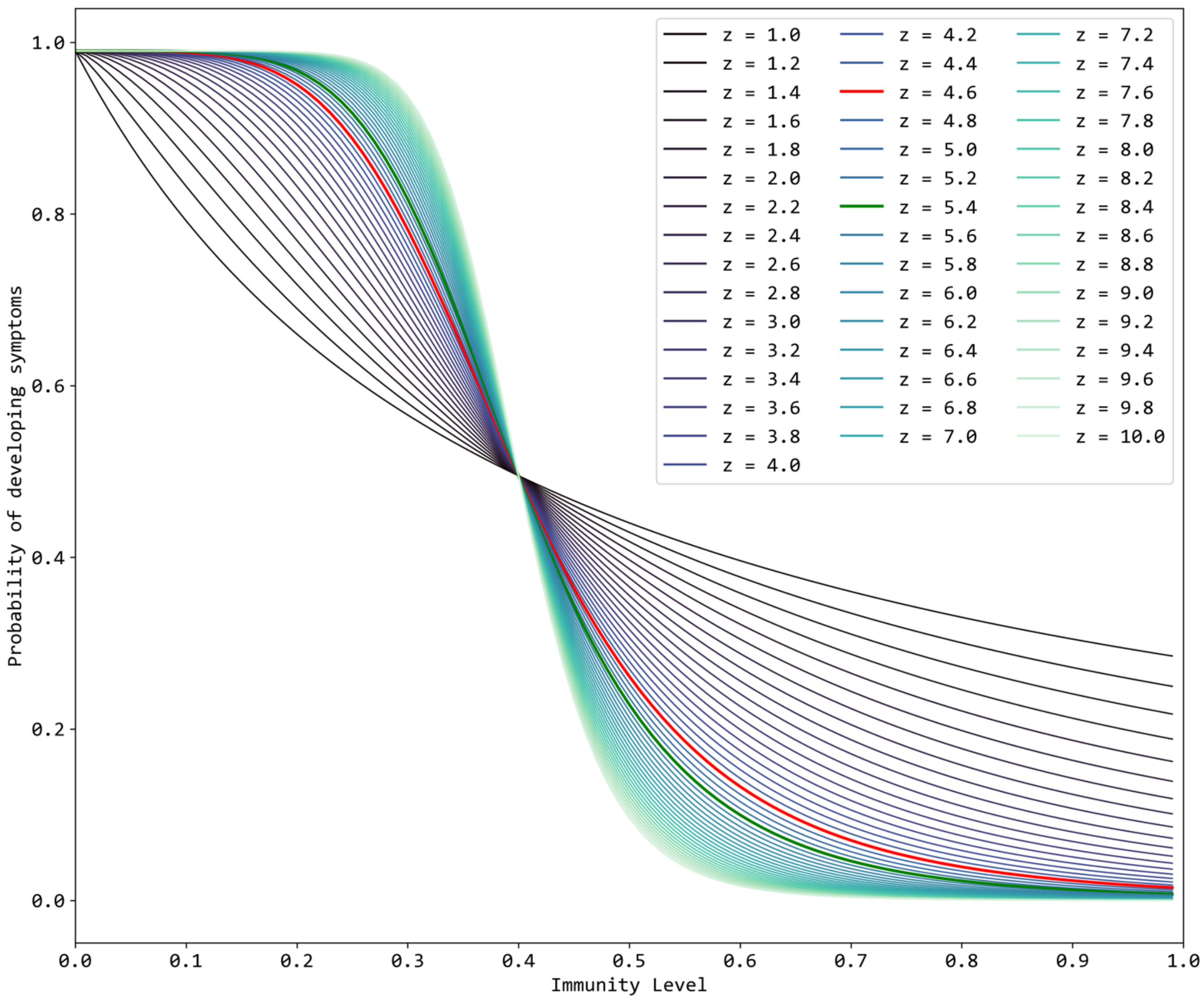

Figure I: The probability of progressing to clinical disease after an infectious bite (i.e. a symptomatic case) based on the host's immune level and the parameter *z*, according to Nguyen et al [12]. The red (*z* = 4.6, for the no treatment scenarios in Figure K) and green (*z* = 5.4, for the 50% treatment coverage scenarios in the remaining figures) lines denote the selected z after calibration.

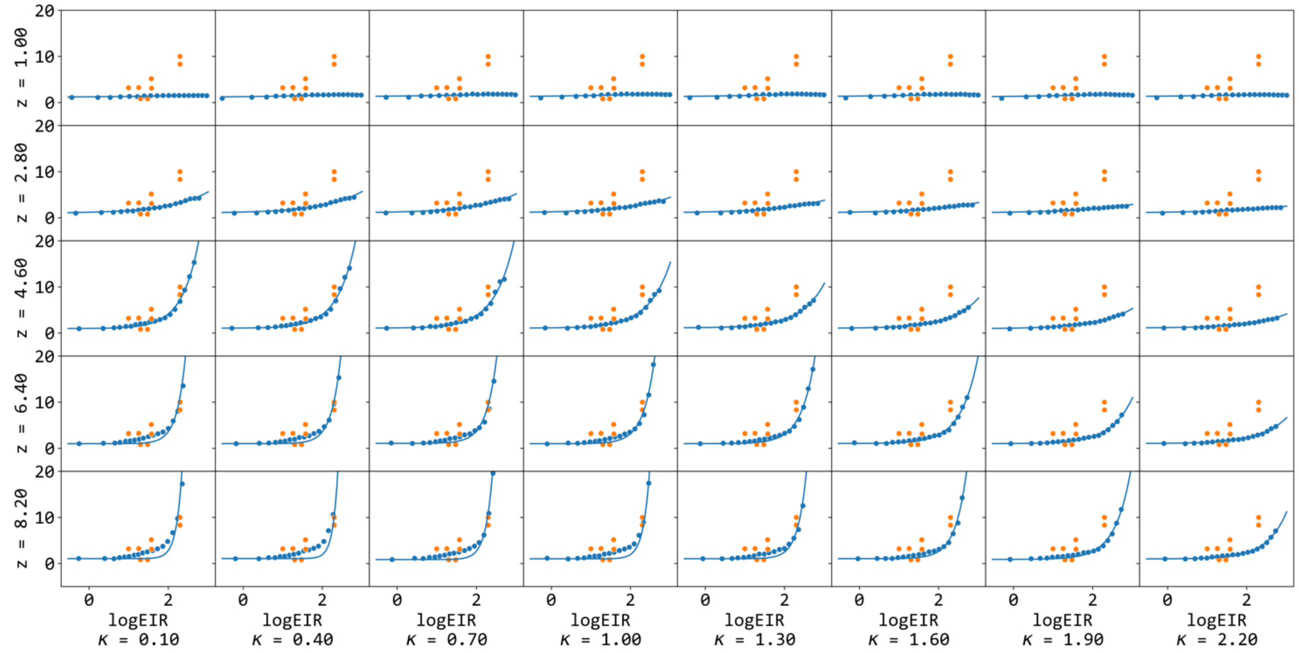

Figure J: Fitting of κ and z values. The y-axis shows the ratio of the number of annual clinical episodes in 2-year-olds to the number in 10-year-olds plotted against $\log_{10}$-EIR. Ten simulations are run for each combination of κ and z (dots in blue) and those combinations are shown next to the data from Table S7 in Nguyen et al [12] (dots in orange). Treatment coverage here is 50% with an 80% efficacy, 7-day half-life drug.

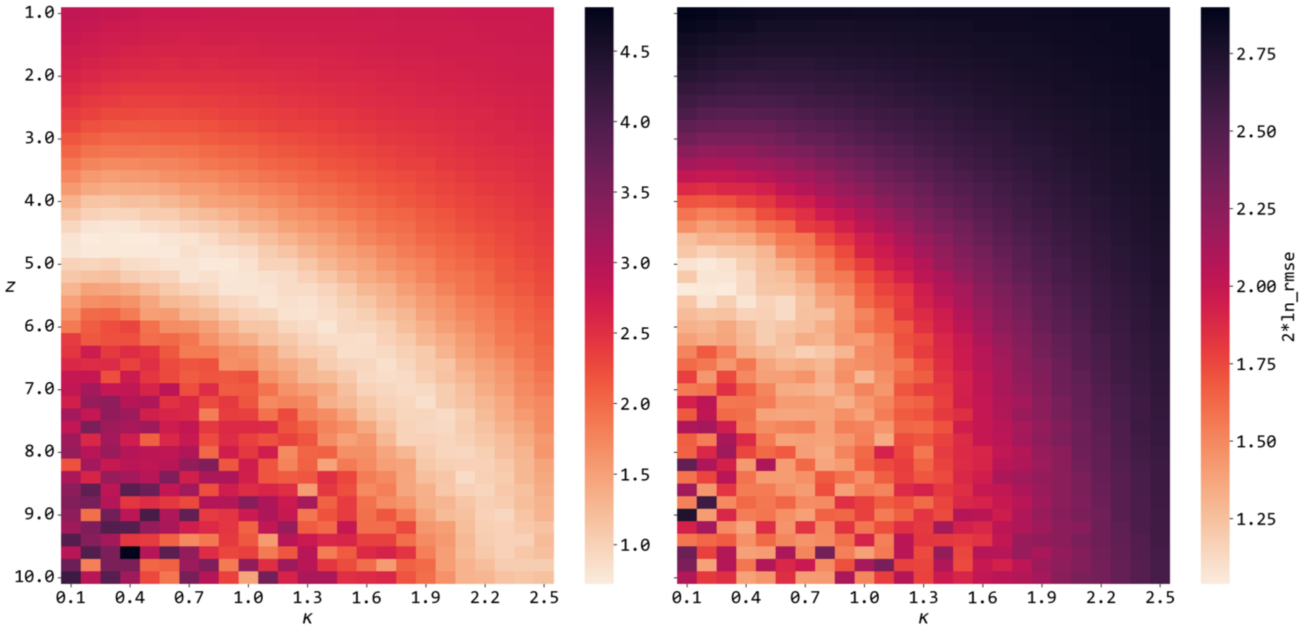

Figure K: Heatmap shows the log sum of squared errors between the blue and orange dots in Figure J. The left heatmap presents simulations with no treatment and the optimal fit z and κ (the brightest spot in the heatmap) were 4.6 and 0.2 respectively. When treatment coverage is 50% (80% efficacy drug with a 7-day half-life) the optimal values are $\kappa = 0.3$ and $z = 5.4$, corresponding to the brightest spot in the right heatmap.

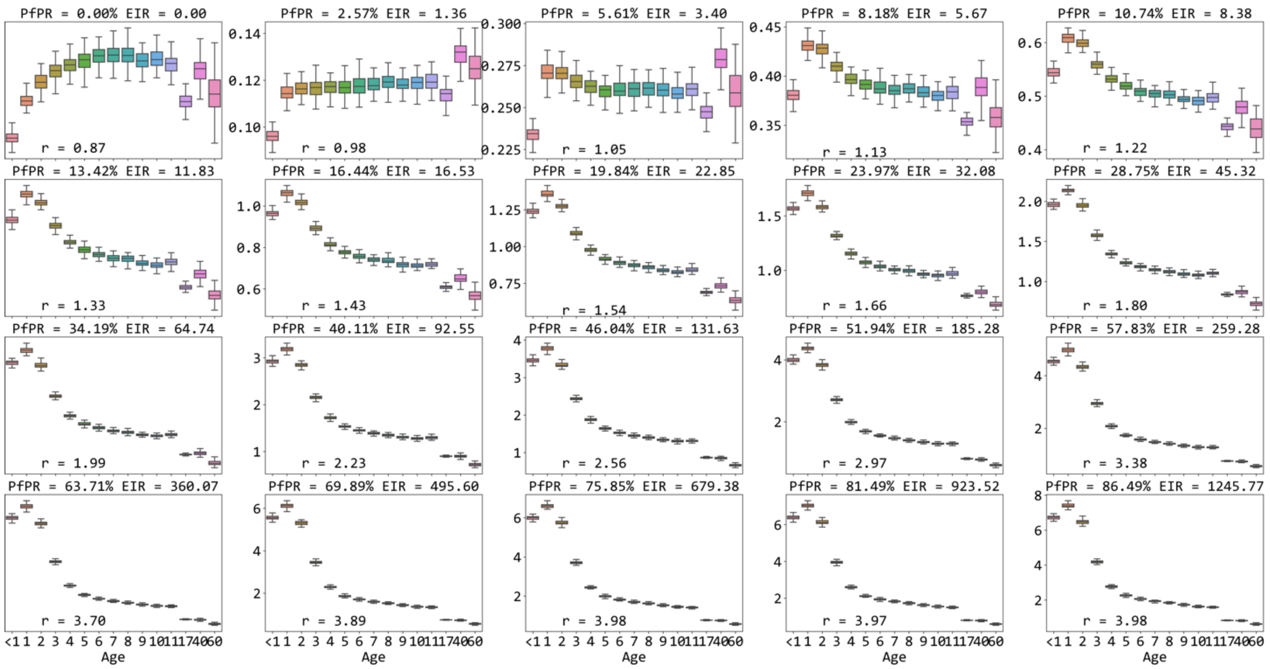

Figure L: The age-specific clinical episodes per year, under different transmission intensities (EIR increases from left to right and from top to bottom). The *r*-values in the bottom left corner show the ratio of clinical episodes in two-year olds to clinical episodes in ten-year olds in the simulation. Boxplots show medians and interquartile ranges from 100 simulations. All simulations run with $\kappa = 0.3$ and $z = 5.4$ with 50% treatment coverage with a 7-day half-life drug of approximately 80% efficacy.

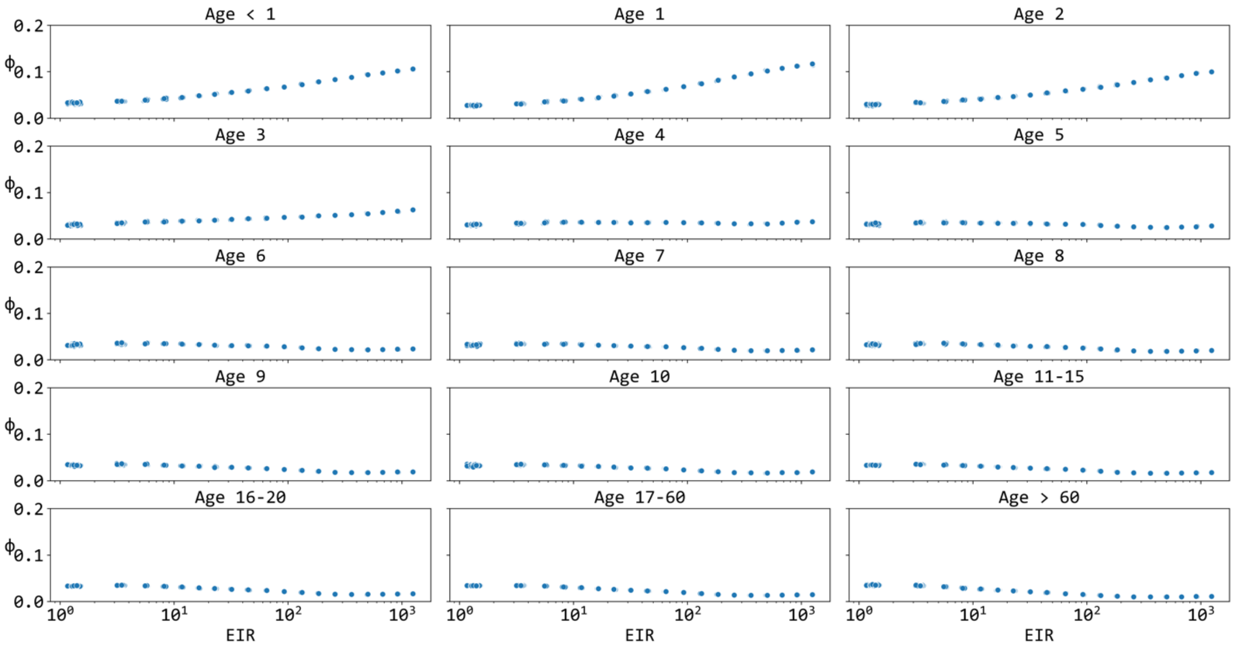

Figure M: 100 simulations were run to equilibrium at different EIR levels ranging from 1.0 to >1000. Treatment coverage is 50%, with a 7-day half-life drug of approximately 80% efficacy. Immune acquisition parameters $\kappa =$ 0.3 and $z = 5.4$. The y-axis value φ shows what fraction of infections are in individuals experiencing symptoms.

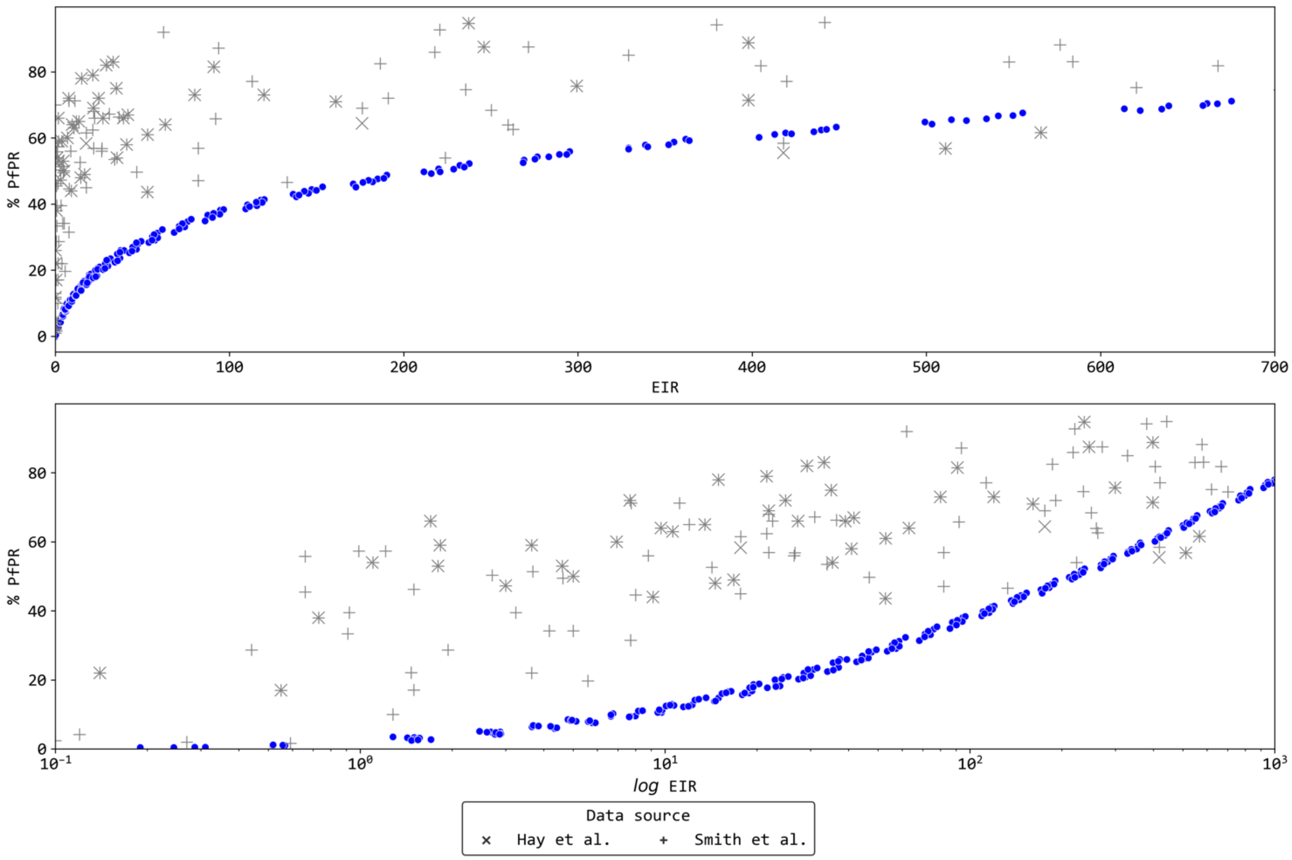

Figure N: Relationship between EIR and blood slide prevalence shown on linear scale (top) and log scale (bottom). The data points in blue dot are from the model, and parasite positive individuals are counted if their parasitaemia is higher than 10 parasites per microliter. The data points with "+" and "×" symbols are data from Smith et al [35] and Hay et al and Cameron et al [36,37]. Model output shown in blue circles. The model was run with Γ-distributed relative biting rate with a coefficient of variation equal to 2.0. Treatment coverage is 50% with a 7-day half-life drug of approximately 80% efficacy.

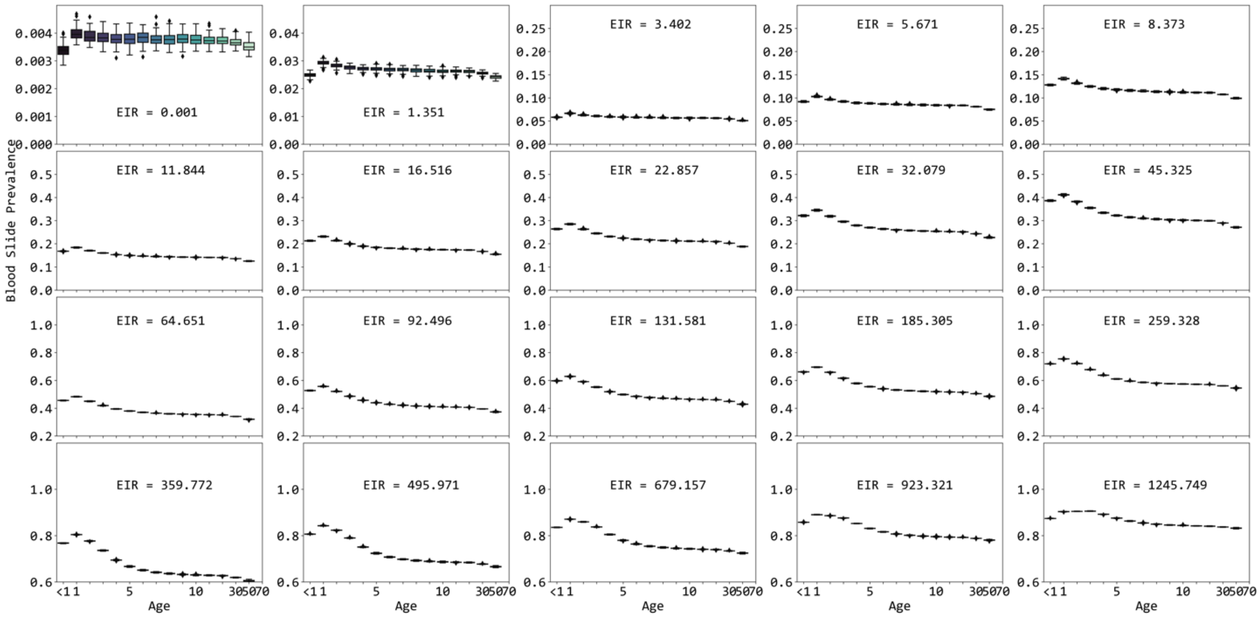

Figure O: Panels shows age-specific blood-slide prevalence for different transmission intensities; $\kappa = 0.3$, $z = 5.4$, treated coverage is 50% with a 7-day half-life drug of approximately 80% efficacy.

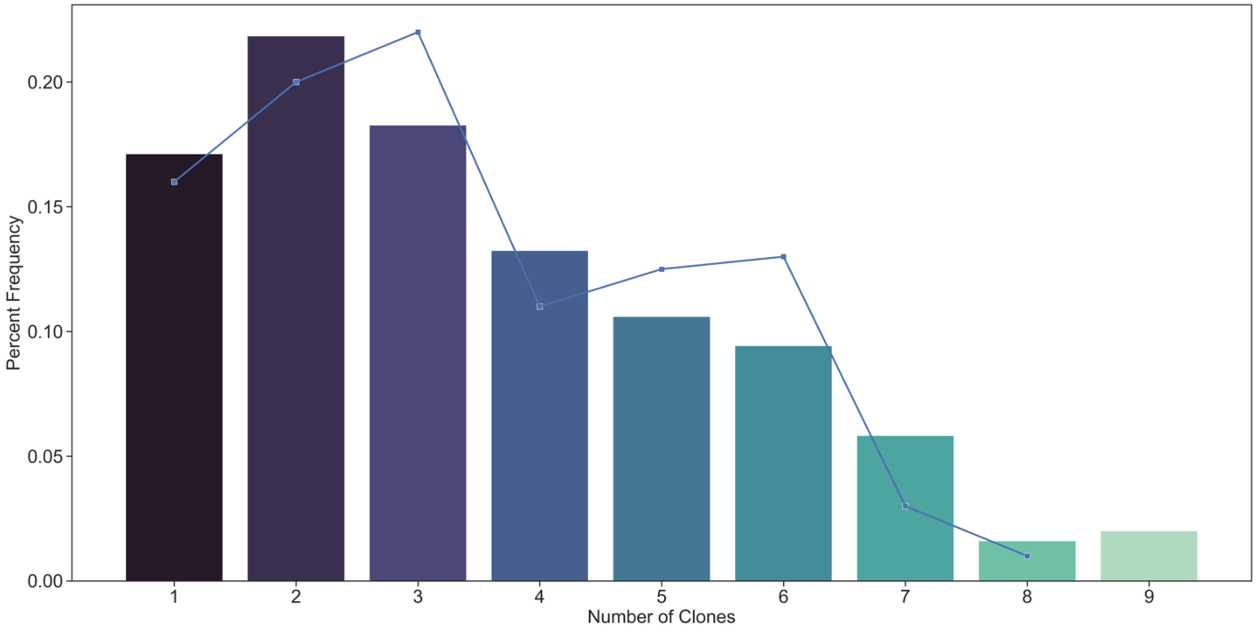

Figure P: Distribution of number of clones per infection. The bar graph shows the output from our simulation with different EIR ($\kappa = 0.3$, $z = 5.4$, 50% treatment coverage, with a 7-day half-life drug of approximately 80% efficacy).

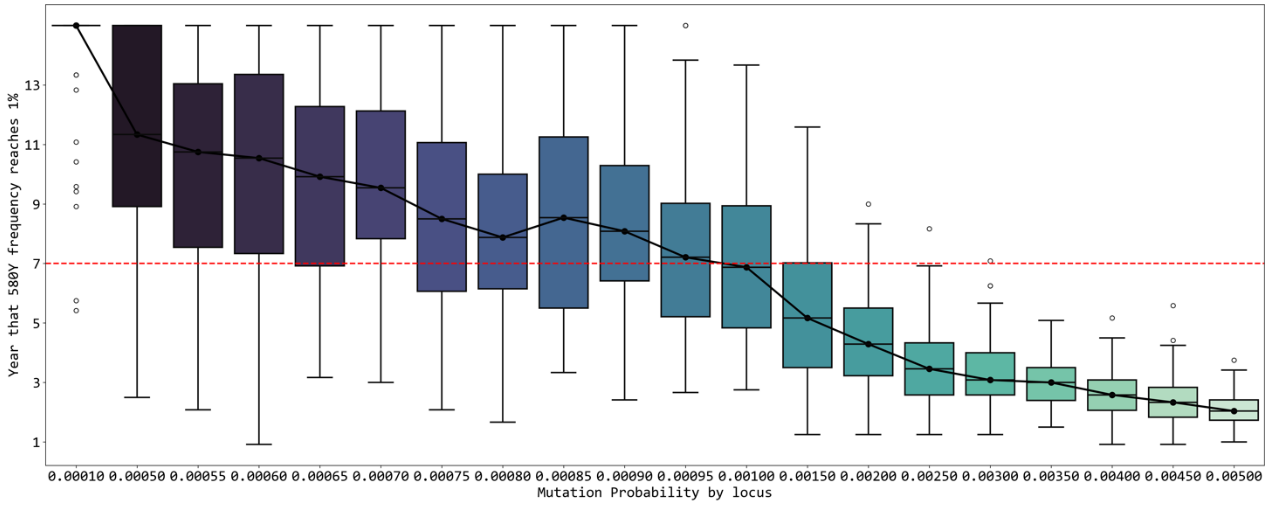

Figure Q: Calibration of mutation probability in the model. Target calibration is to have 580Y frequency reach 0.01 allele frequency after 7.0 years of treatment with DHA-PPQ with 40% treatment coverage, at 25% prevalence and c_R = 0.0005. The x-axis shows different values of the model variable mutation_probability_per_locus (mutation and within-host fixation probability if treatment is given that selects for that particular mutation) while the y-axis shows the year that 580Y frequency reaches 0.01. The calibration-selected value is mutation_probability_per_locus = 0.001.

Reference

1. Li EZ, Nguyen TD, Tran TN-A, Zupko RJ, Boni MF. Assessing emergence risk of double-resistant and triple-resistant genotypes of Plasmodium falciparum. Nat Commun. 2024;15: 1390. doi:10.1038/s41467-024-45547-x
2. Nguyen TD, Tran TN-A, Parker D, White N, Boni MF. Antimalarial mass drug administration in large populations and the evolution of drug resistance. PLOS Glob Public Health. 2023;3: e0002200. doi:10.1371/journal.pgph.0002200
3. Zupko RJ, Nguyen TD, Ngabonziza JCS, Kabera M, Li H, Tran TN-A, et al. Modeling policy interventions for slowing the spread of artemisinin-resistant pfkelch R561H mutations in Rwanda. Nat Med. 2023;29: 2775–2784. doi:10.1038/s41591-023-02551-w
4. Zupko RJ, Nguyen TD, Wesolowski A, Gerardin J, Boni MF. National-scale simulation of human movement in a spatially coupled individual-based model of malaria in Burkina Faso. Sci Rep. 2023;13: 321. doi:10.1038/s41598-022-26878-5
5. Zupko RJ, Servadio JL, Nguyen TD, Tran TN-A, Tran KT, Somé AF, et al. Role of Seasonal Importation and Random Genetic Drift on Selection for Drug-Resistant Genotypes of *Plasmodium falciparum* in High Transmission Settings. Genetics; 2023. doi:10.1101/2023.10.20.563204
6. Zupko RJ, Nguyen TD, Somé AF, Tran TN-A, Gerardin J, Dudas P, et al. Long-term effects of increased adoption of artemisinin combination therapies in Burkina Faso. PLOS Glob Public Health. 2022;2: e0000111. doi:10.1371/journal.pgph.0000111
7. Okombo J, Mok S, Qahash T, Yeo T, Bath J, Orchard LM, et al. Piperaquine-resistant PfCRT mutations differentially impact drug transport, hemoglobin catabolism and parasite physiology in Plasmodium falciparum asexual blood stages. PLOS Pathog. 2022;18: e1010926. doi:10.1371/journal.ppat.1010926
8. Ross A, Killeen G, Smith T. RELATIONSHIPS BETWEEN HOST INFECTIVITY TO MOSQUITOES AND ASEXUAL PARASITE DENSITY IN PLASMODIUM FALCIPARUM. Am J Trop Med Hyg. 2006;75: 32–37. doi:10.4269/ajtmh.2006.75.32
9. Ross LS, Dhingra SK, Mok S, Yeo T, Wicht KJ, Kümpornsin K, et al. Emerging Southeast Asian PfCRT mutations confer Plasmodium falciparum resistance to the first-line antimalarial piperaquine. Nat Commun. 2018;9: 3314. doi:10.1038/s41467-018-05652-0
10. Small-Saunders JL, Hagenah LM, Wicht KJ, Dhingra SK, Deni I, Kim J, et al. Evidence for the early emergence of piperaquine-resistant Plasmodium falciparum malaria and modeling strategies to mitigate resistance. PLOS Pathog. 2022;18: e1010278. doi:10.1371/journal.ppat.1010278
11. Watson OJ, Gao B, Nguyen TD, Tran TN-A, Penny MA, Smith DL, et al. Pre-existing partner-drug resistance to artemisinin combination therapies facilitates the emergence and spread of artemisinin resistance: a consensus modelling study. Lancet Microbe. 2022;3: e701–e710. doi:10.1016/S2666-5247(22)00155-0
12. Nguyen TD, Olliaro P, Dondorp AM, Baird JK, Lam HM, Farrar J, et al. Optimum population-level use of artemisinin combination therapies: a modelling study. Lancet Glob Health. 2015;3: e758–e766. doi:10.1016/S2214-109X(15)00162-X
13. Maire N, Smith T, Ross A, Owusu-Agyei S, Dietz K, Molineaux L. A MODEL FOR NATURAL IMMUNITY TO ASEXUAL BLOOD STAGES OF PLASMODIUM FALCIPARUM MALARIA IN ENDEMIC AREAS. Am J Trop Med Hyg. 2006;75: 19–31. doi:10.4269/ajtmh.2006.75.19
14. Eyles DE, Young MD. The duration of untreated or inadequately treated Plasmodium falciparum infections in the human host. J Natl Malar Soc US. 1951;10: 327–336.
15. Molineaux L, Gramiccia G. The Garki project: research on the epidemiology and control of malaria in the Sudan Savanna of West Africa. Geneva: World Health Organization; 1980.
16. Anderson TJC, Paul REL, Donnelly CA, DAY KP. Do malaria parasites mate non-randomly in the mosquito midgut? Genet Res. 2000;75: 285–296. doi:10.1017/S0016672300004481
17. Arez AP, Pinto J, Pålsson K, Snounou G, Jaenson TGT, Do Rosário VE. Transmission of mixed Plasmodium species and Plasmodium falciparum genotypes. Am J Trop Med Hyg. 2003;68: 161–168. doi:10.4269/ajtmh.2003.68.2.0680161
18. Das S, Muleba M, Stevenson JC, Pringle JC, Norris DE. Beyond the entomological inoculation rate: characterizing multiple blood feeding behavior and Plasmodium falciparum multiplicity of infection

in Anopheles mosquitoes in northern Zambia. Parasit Vectors. 2017;10: 45. doi:10.1186/s13071-017-1993-z

19. Norris LC, Fornadel CM, Hung W-C, Pineda FJ, Norris DE. Frequency of Multiple Blood Meals Taken in a Single Gonotrophic Cycle by Anopheles arabiensis Mosquitoes in Macha, Zambia. Am Soc Trop Med Hyg. 2010;83: 33–37. doi:10.4269/ajtmh.2010.09-0296
20. Gonçalves AALM, Dias AHC, Monteiro DDS, Varela IBF, da Veiga Leal S. Blood meal survey reveals insights into mosquito-borne diseases on the island of Santiago, Cape Verde. Front Trop Dis. 2023;4. doi:10.3389/fitd.2023.1070172
21. Keven JB, Katusele M, Vinit R, Rodríguez-Rodríguez D, Hetzel MW, Robinson LJ, et al. Nonrandom Selection and Multiple Blood Feeding of Human Hosts by Anopheles Vectors: Implications for Malaria Transmission in Papua New Guinea. Am J Trop Med Hyg. 2021;105: 1747–1758. doi:10.4269/ajtmh.21-0210
22. Scott TW, Githeko AK, Fleisher A, Harrington LC, Yan G. DNA profiling of human blood in anophelines from lowland and highland sites in western Kenya. Am J Trop Med Hyg. 2006;75: 231–7.
23. Shaw WR, Holmdahl IE, Itoe MA, Werling K, Marquette M, Paton DG, et al. Multiple blood feeding in mosquitoes shortens the Plasmodium falciparum incubation period and increases malaria transmission potential. PLoS Pathog. 2020;16: e1009131. doi:10.1371/journal.ppat.1009131
24. Soremekun S, Maxwell C, Zuwakuu M, Chen C, Michael E, Curtis C. Measuring the efficacy of insecticide treated bednets: the use of DNA fingerprinting to increase the accuracy of personal protection estimates in Tanzania. Trop Med Int Health TM IH. 2004;9: 664–72. doi:10.1111/j.1365-3156.2004.01250.x
25. Webber LA, Edman JD, Kale HW. Effect of Mosquito Density on the Interrelationship of Host Behavior and Mosquito Feeding Success *. Am J Trop Med Hyg. 1972;21: 487–491. doi:10.4269/ajtmh.1972.21.487
26. Boni MF, Smith DL, Laxminarayan R. Benefits of using multiple first-line therapies against malaria. Proc Natl Acad Sci. 2008;105: 14216–14221. doi:10.1073/pnas.0804628105
27. Agrawal S, Moser KA, Morton L, Cummings MP, Parihar A, Dwivedi A, et al. Association of a Novel Mutation in the Plasmodium falciparum Chloroquine Resistance Transporter With Decreased Piperaquine Sensitivity. J Infect Dis. 2017;216: 468–476. doi:10.1093/infdis/jix334
28. Van Der Pluijm RW, Imwong M, Chau NH, Hoa NT, Thuy-Nhien NT, Thanh NV, et al. Determinants of dihydroartemisinin-piperaquine treatment failure in Plasmodium falciparum malaria in Cambodia, Thailand, and Vietnam: a prospective clinical, pharmacological, and genetic study. Lancet Infect Dis. 2019;19: 952–961. doi:10.1016/S1473-3099(19)30391-3
29. WWARN K13 Genotype-Phenotype Study Group. Association of mutations in the Plasmodium falciparum Kelch13 gene (Pf3D7_1343700) with parasite clearance rates after artemisinin-based treatments—a WWARN individual patient data meta-analysis. BMC Med. 2019;17: 1. doi:10.1186/s12916-018-1207-3
30. Abdullah S, Adazu K, Masanja H, Diallo D, Hodgson A, Ilboudo-Sanogo E, et al. Patterns of age-specific mortality in children in endemic areas of sub-Saharan Africa. Am J Trop Med Hyg. 2007;77: 99–105.
31. Myint HY, Tipmanee P, Nosten F, Day NPJ, Pukrittayakamee S, Looareesuwan S, et al. A systematic overview of published antimalarial drug trials. Trans R Soc Trop Med Hyg. 2004;98: 73–81. doi:10.1016/S0035-9203(03)00014-2
32. Nguyen TD, Gao B, Amaratunga C, Dhorda M, Tran TN-A, White NJ, et al. Preventing antimalarial drug resistance with triple artemisinin-based combination therapies. Nat Commun. 2023;14: 4568. doi:10.1038/s41467-023-39914-3
33. Beier JC, Oster CN, Onyango FK, Bales JD, Sherwood JA, Perkins PV, et al. Plasmodium falciparum Incidence Relative to Entomologic Inoculation Rates at a Site Proposed for Testing Malaria Vaccines in Western Kenya. Am J Trop Med Hyg. 1994;50: 529–536. doi:10.4269/ajtmh.1994.50.529
34. Filipe JAN, Riley EM, Drakeley CJ, Sutherland CJ, Ghani AC. Determination of the Processes Driving the Acquisition of Immunity to Malaria Using a Mathematical Transmission Model. De Boer RJ, editor. PLoS Comput Biol. 2007;3: e255. doi:10.1371/journal.pcbi.0030255

35. Smith DL, Dushoff J, Snow RW, Hay SI. The entomological inoculation rate and Plasmodium falciparum infection in African children. Nature. 2005;438: 492–495. doi:10.1038/nature04024
36. Cameron E, Battle KE, Bhatt S, Weiss DJ, Bisanzio D, Mappin B, et al. Defining the relationship between infection prevalence and clinical incidence of Plasmodium falciparum malaria. Nat Commun. 2015;6: 8170. doi:10.1038/ncomms9170
37. Hay SI, Guerra CA, Tatem AJ, Atkinson PM, Snow RW. Urbanization, malaria transmission and disease burden in Africa. Nat Rev Microbiol. 2005;3: 81–90. doi:10.1038/nrmicro1069
