# Supplementary material for: Effects of recombination on multi-drug resistance evolution in Plasmodium falciparum malaria
Source: PLoS Comput Biol. 2025 Aug 25;21(8):e1013401. doi: 10.1371/journal.pcbi.1013401 (PMC12377563; doi:10.1371/journal.pcbi.1013401)
Supplement: S2 Text — (PDF) [file pcbi.1013401.s002.pdf]

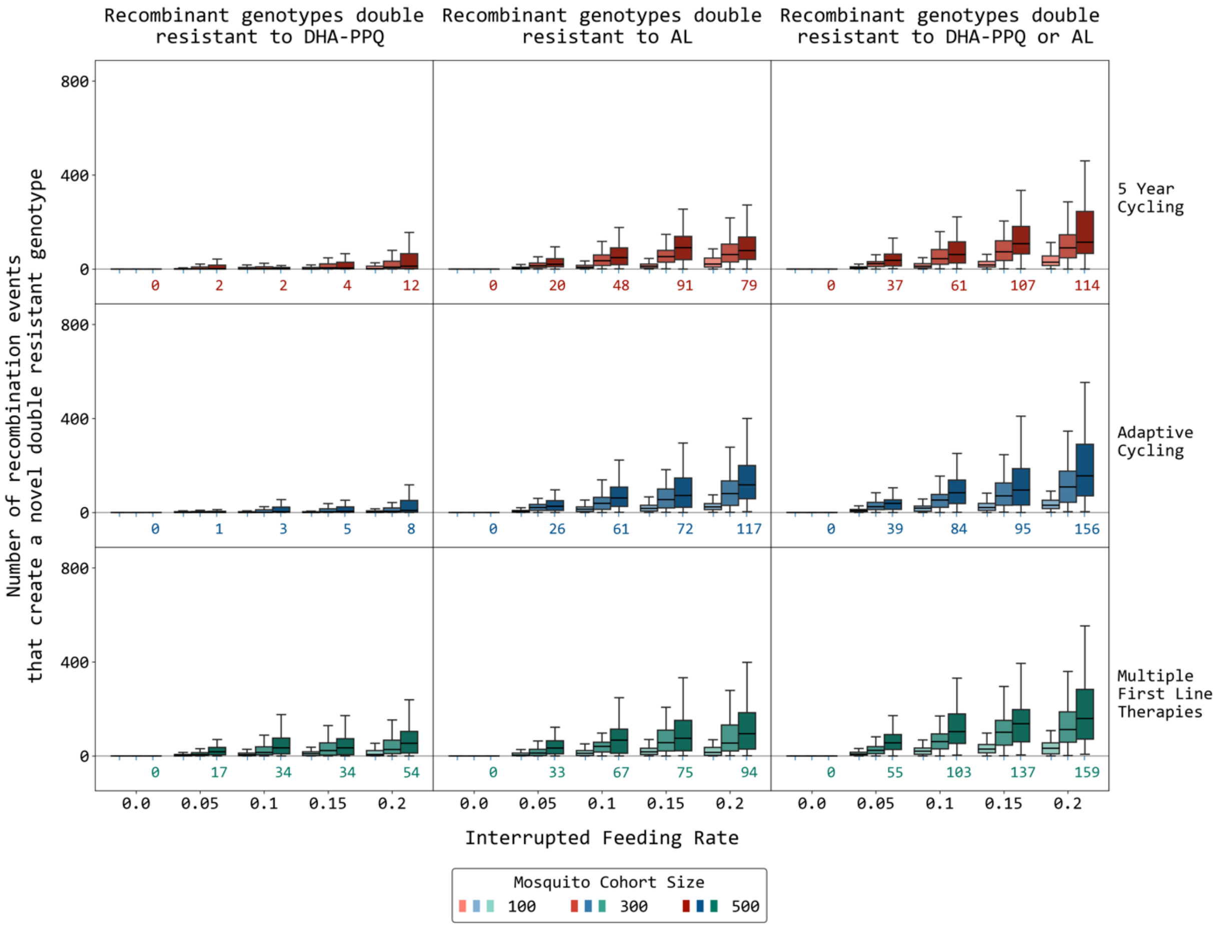

Figure A: Number of recombination events that produce novel recombinant double-resistant genotypes. This figure has the same settings and format as in Figure 1 in the main text but recombination from multi-clonal within-host parasites infection is not activated, meaning all counted recombination events are from mosquito interrupted feeds.

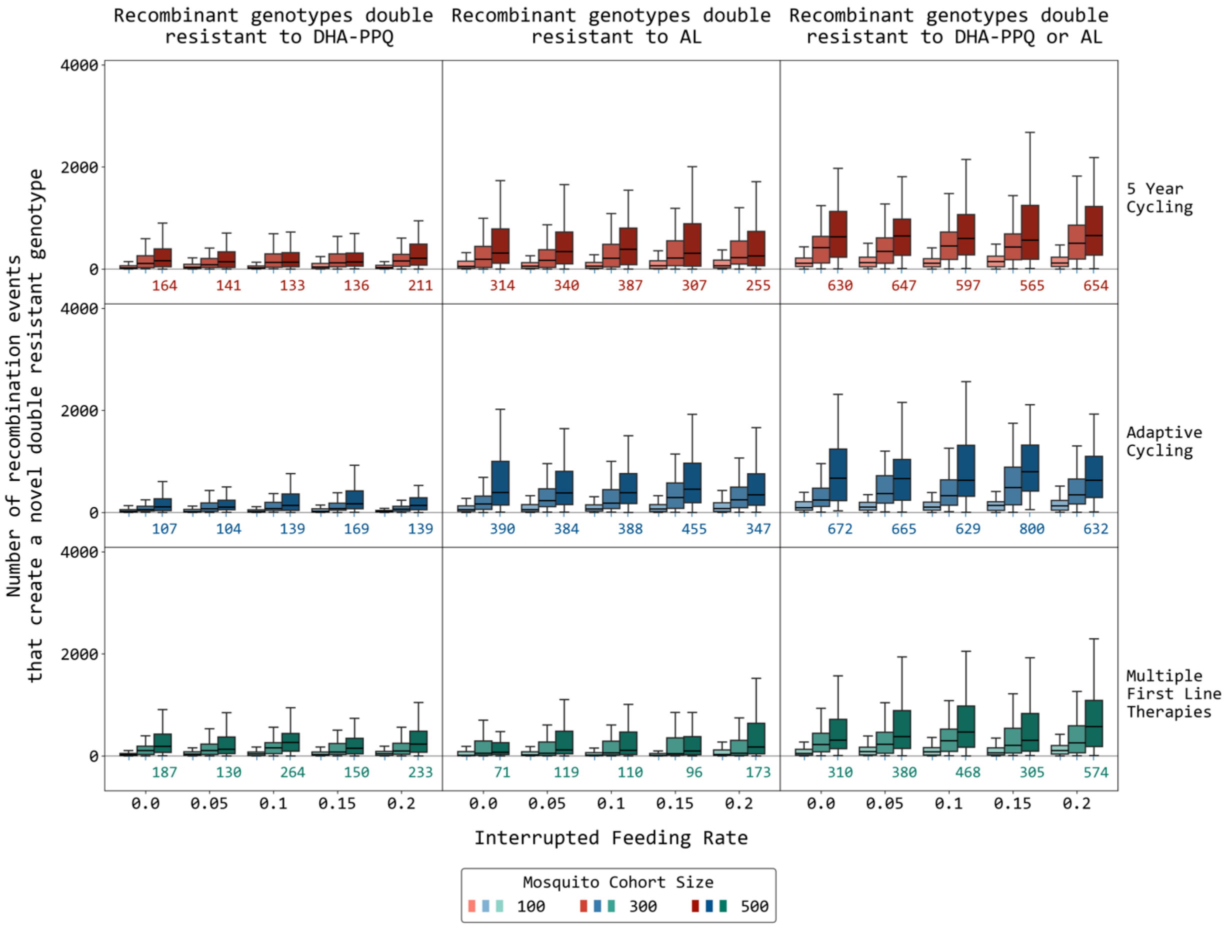

Figure B: Number of recombination events that produce novel recombinant double-resistant genotypes. This figure has the same settings and format as in Figure 1 in the main text but with $c_R = 0.005$.

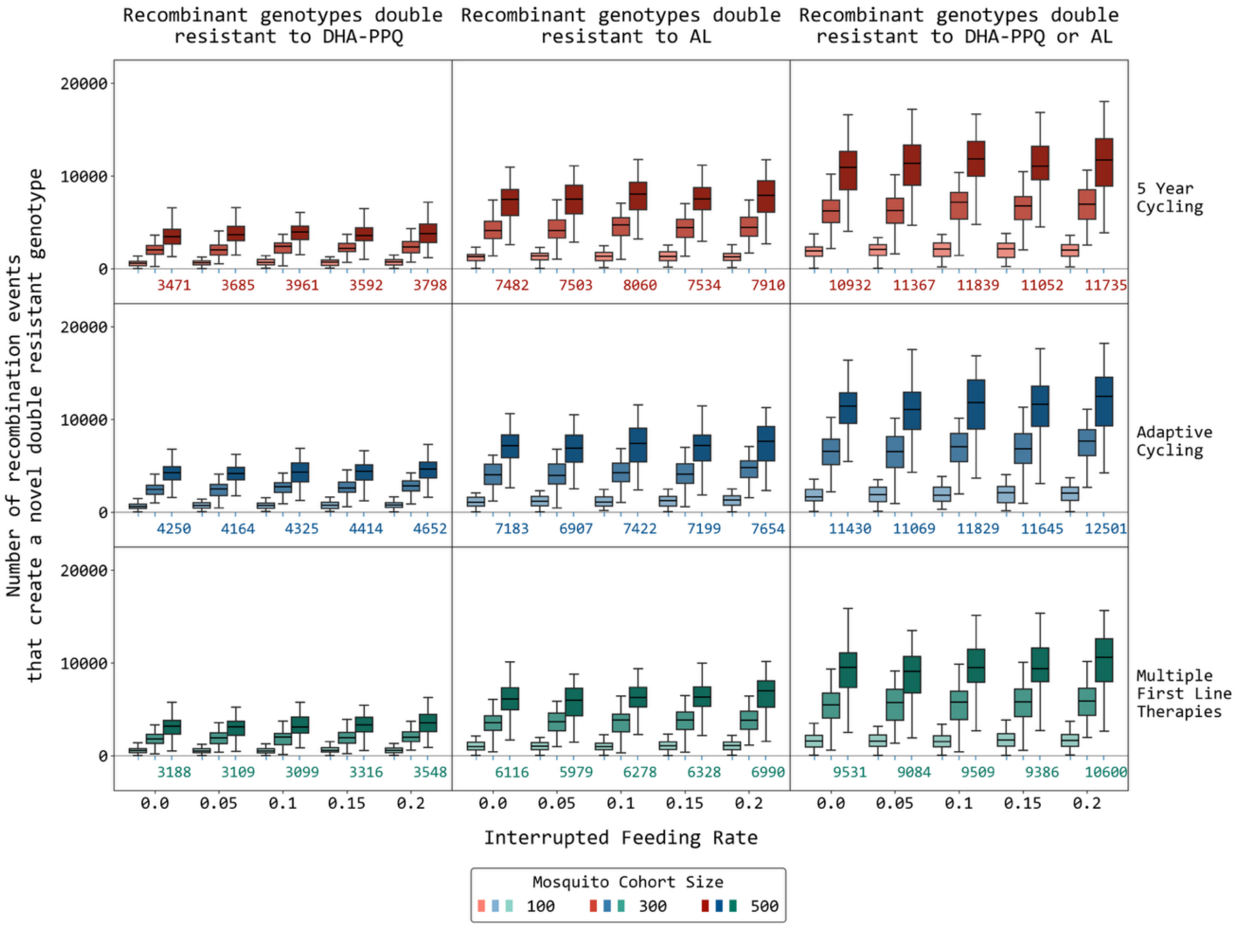

Figure C: Number of recombination events that produce novel recombinant double-resistant genotypes. This figure has the same settings and format as in Figure 1 in the main text but prevalence is 25% and $c_R = 0.0005$.

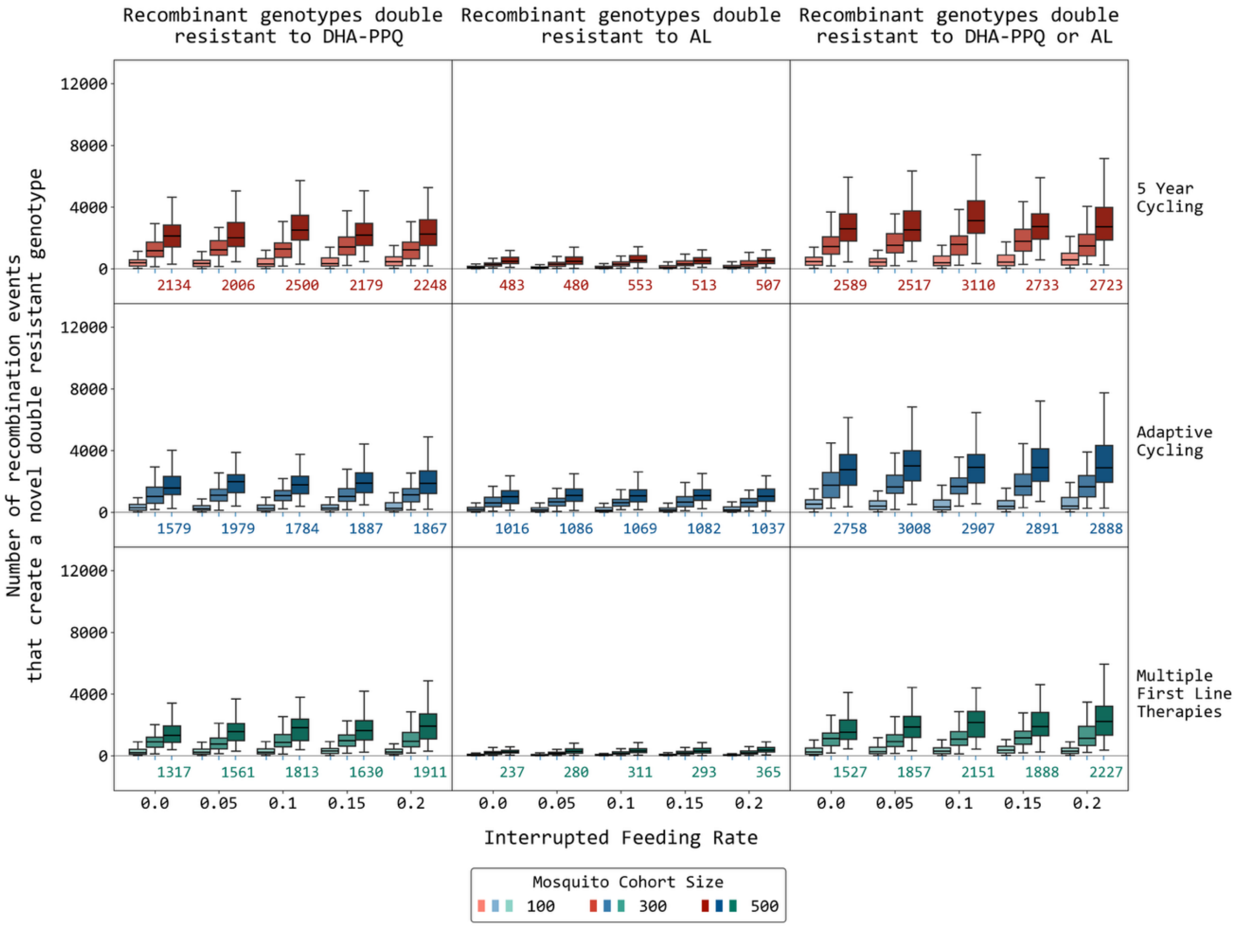

Figure D: Number of recombination events that produce novel recombinant double-resistant genotypes. This figure has the same settings and format as in Figure 1 in the main text but prevalence is 25% and $c_R = 0.005$.

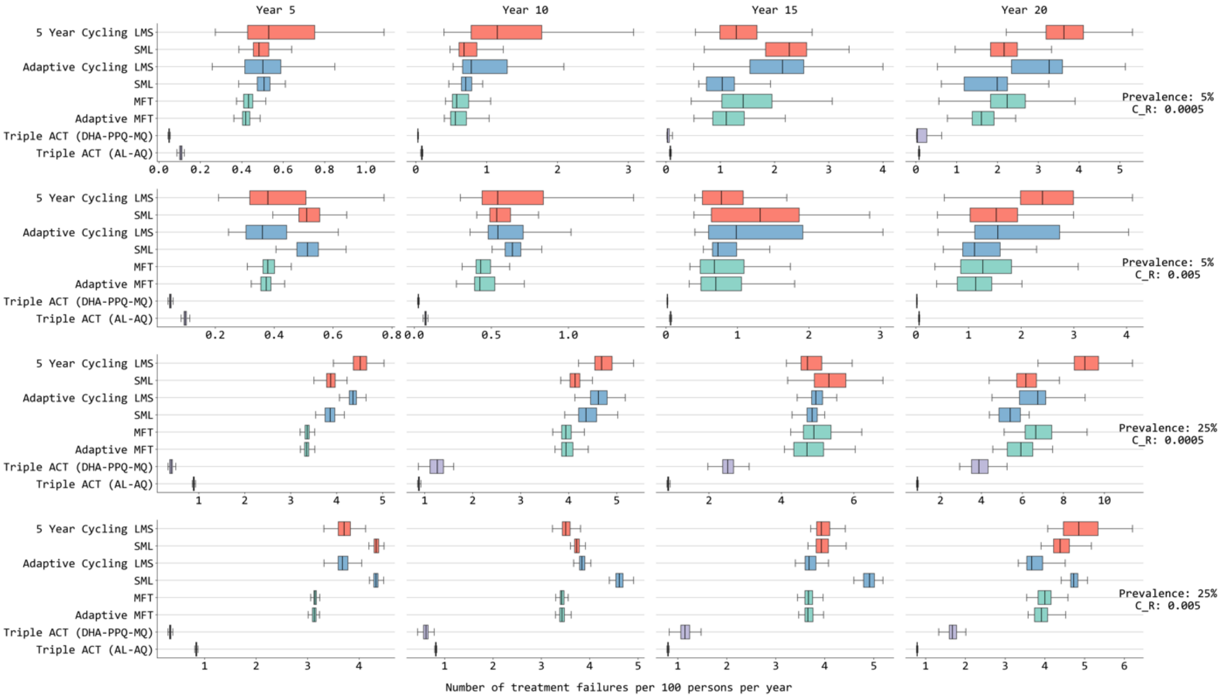

Figure E: Number of treatment failures per 100 persons per year, counted through to year 5, to year 10, to year 15, and to year 20. This figure has the same settings and format as in Figure 3 in the main text but shows the NTF results using different endpoints.

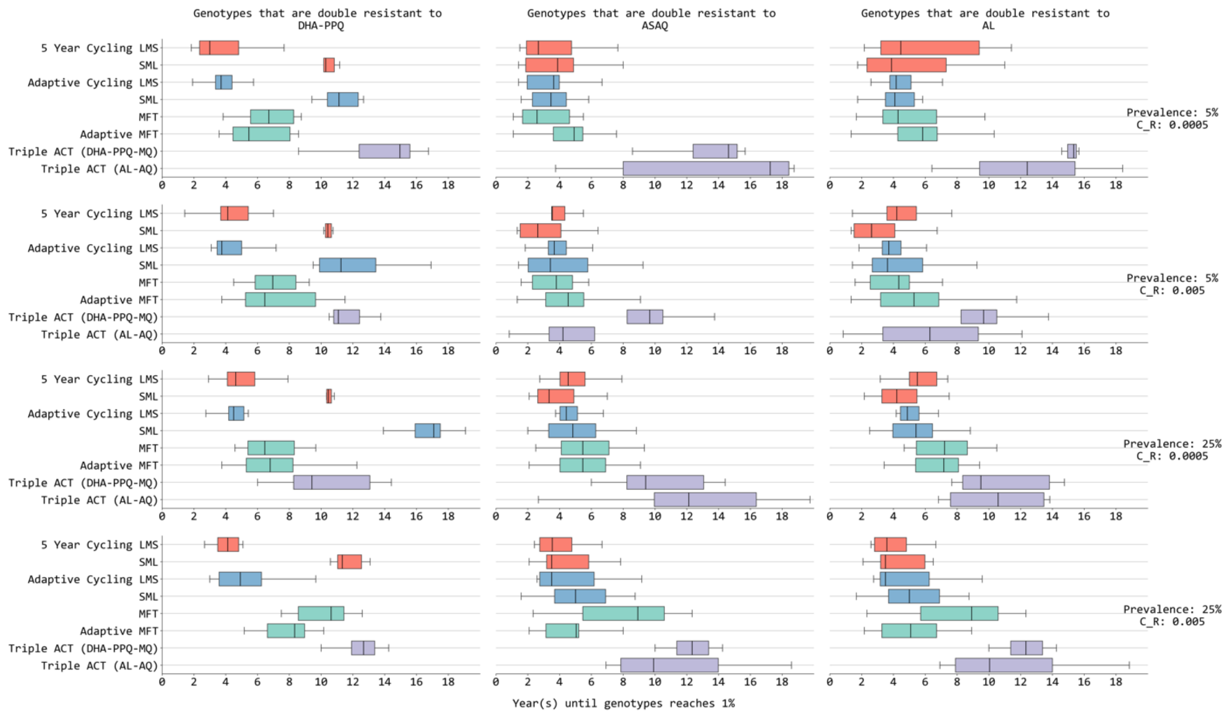

Figure F: Years (*x*-axis) until double-resistant genotype frequency reaches 0.01. Four rows show different malaria prevalence and cost-of-resistance scenarios. Different colors are for different treatment policies: 5-year cycling (light red), adaptive cycling (light blue), multiple first-line therapies (sea green) and triple ACT (violet). LMS indicates that the order of drugs used in cycling strategies is based on the half-life of the partner drug (**L**ong: DHA-PPQ, **M**edium: ASAQ, **S**hort: AL). When no boxplot is shown, this indicates that the genotype frequency is below 0.01 for the entire 20-year period of simulation.

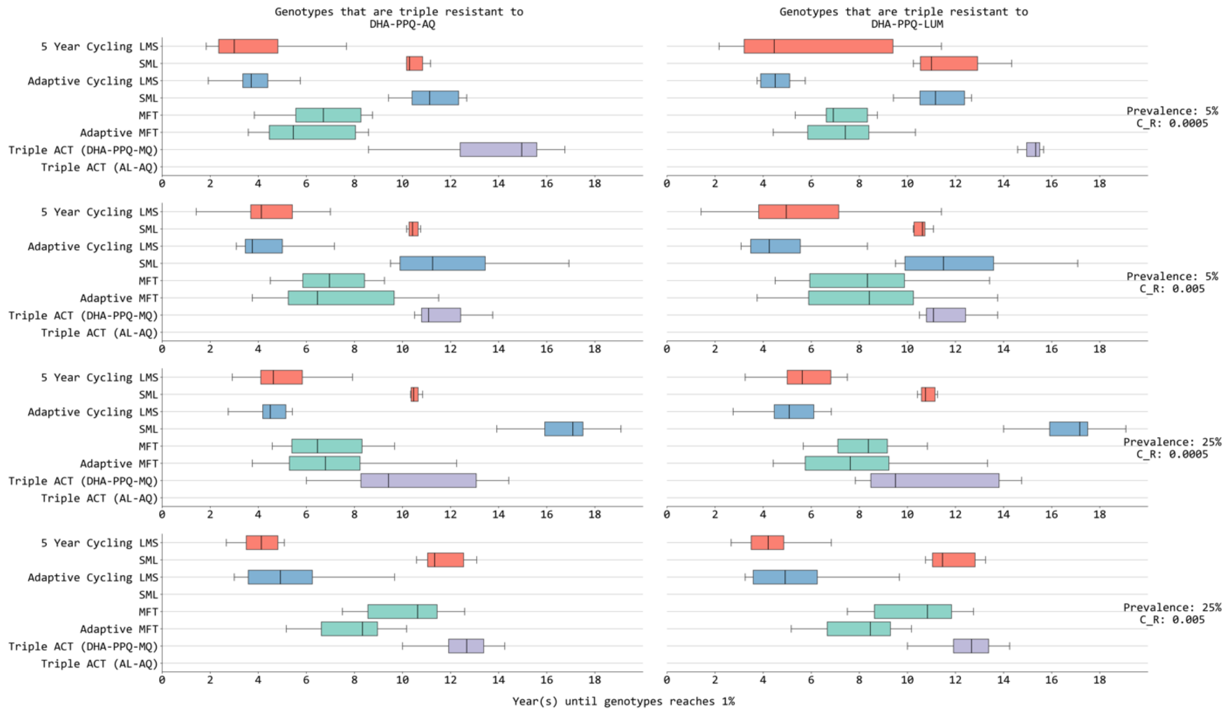

Figure G: Years until triple-resistant genotype frequency reaches 0.01. A single resistance mutation is sufficient to label the parasites as resistant to a particular drug. The four rows show different malaria prevalence and cost of resistance levels. Columns show the two triple therapies (DHA-PPQ-AQ and DHA-PPQ-LUM) to which triple-resistance could emerge. In each panel, the x-axis shows the number of years that it takes for a genotype frequency to reach 0.01 under different treatment strategies: 5-year cycling (light red), adaptive cycling (light blue), multiple first-line therapies (sea green) and triple ACT (violet). LMS indicates the order of therapies used in a cycling strategy based on the half-life of partner drugs (Long: DHA-PPQ, Medium: ASAQ, Short: AL). When no boxplot is shown, this indicates that the genotype frequency is below 0.01 for the entire 20-year period of simulation.

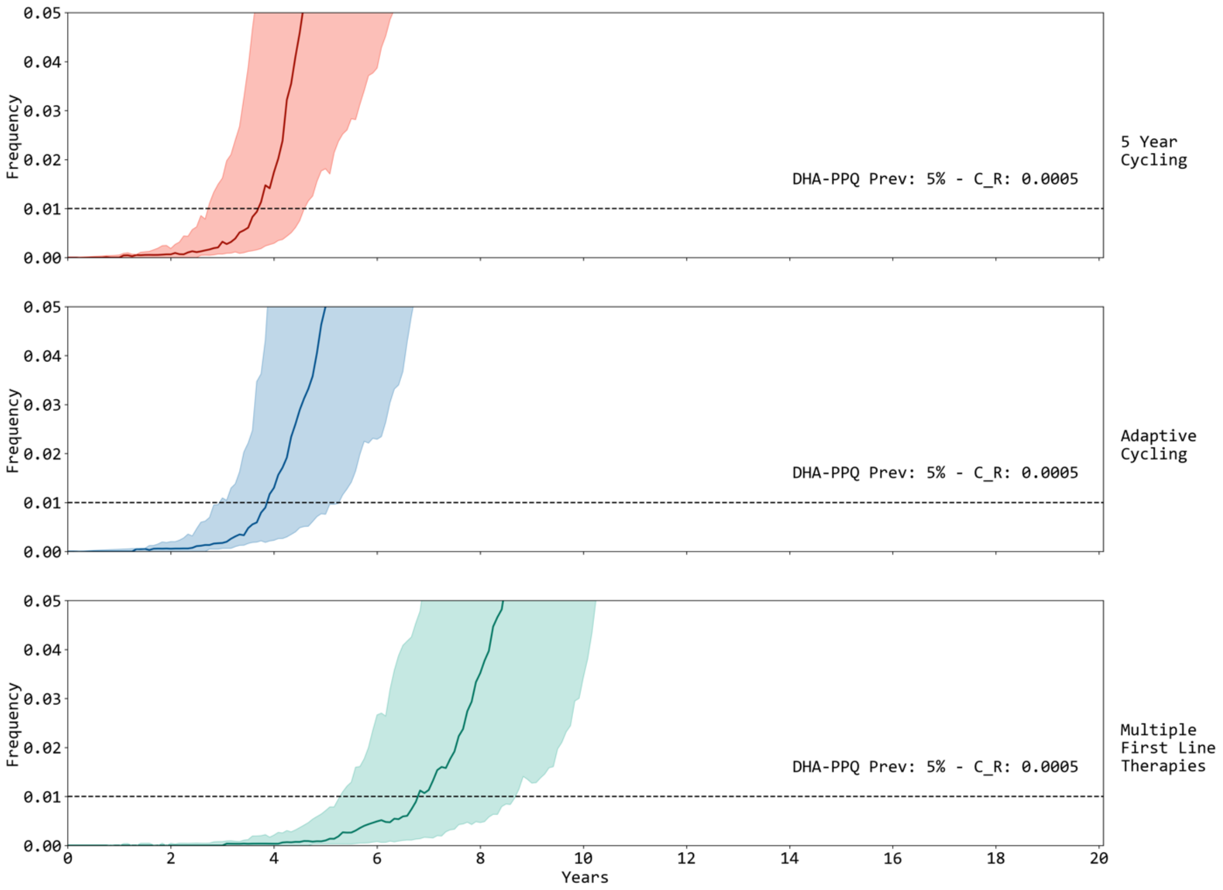

Figure H: Genotype frequency of double-resistants to DHA-PPQ under three different strategies. Year zero indicates the year that the strategy is deployed, and the frequency range is limited to 0.00 to 0.05 to focus on the early period of emergence. Solid line and shaded regions correspond to median and IQR, respectively. Cycling is LMS.

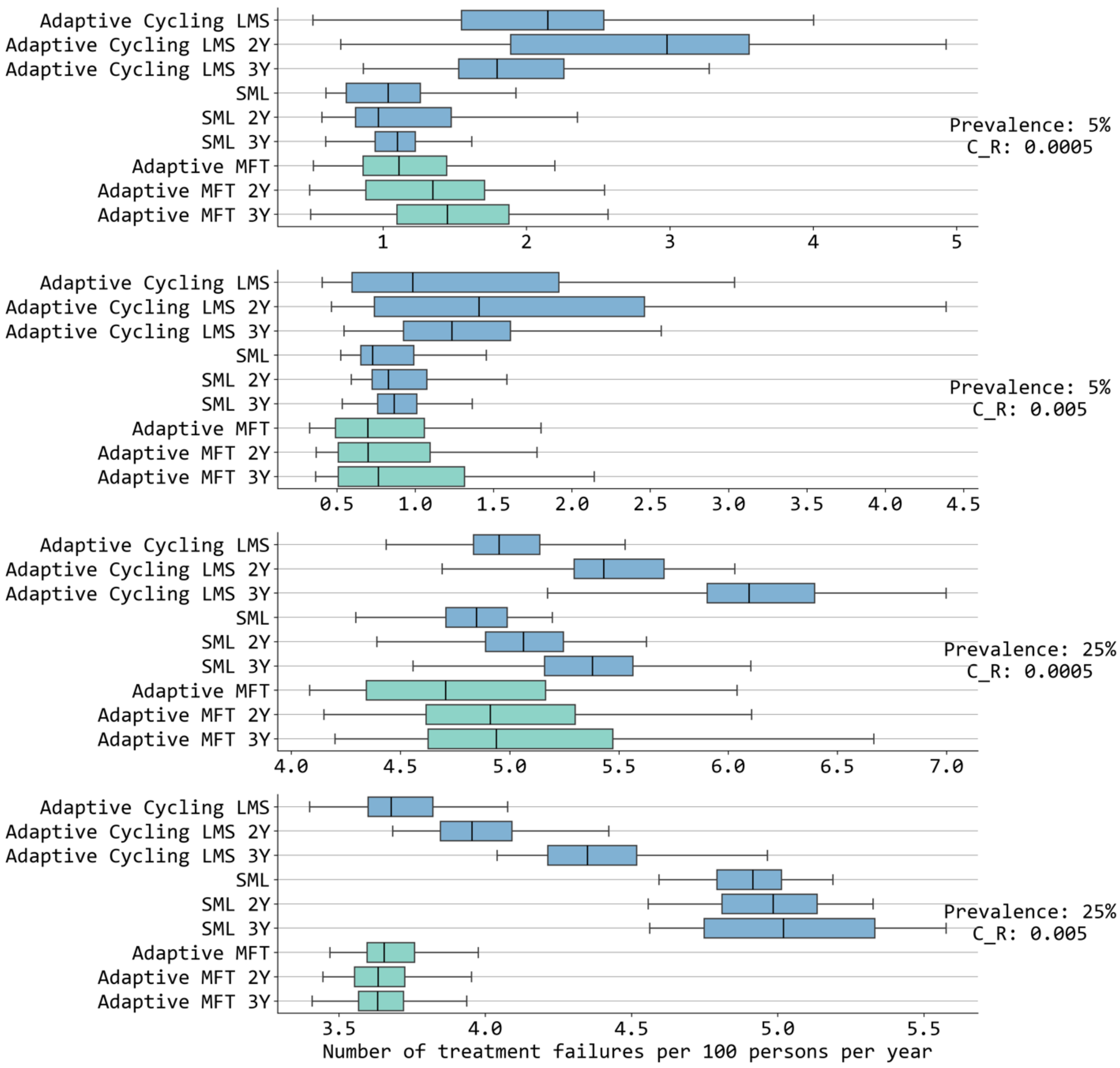

Figure I: Number of treatment failures under adaptive cycling and adaptive MFT policies with different delay times to a policy switch. In our model, adaptive policies include a one-year delay between the trigger (e.g. resistance levels are too high) and the implementation of a new approach. In this panel, "2Y" and "3Y" show the results of two-year and three-year delays. The *x*-axis shows the number of treatment failures per 100 people per year. LMS indicates the order of drugs used in cycling strategies based on efficient period of partner drugs (**L**ong: DHA-PPQ, **M**edium: ASAQ, **S**hort: AL).

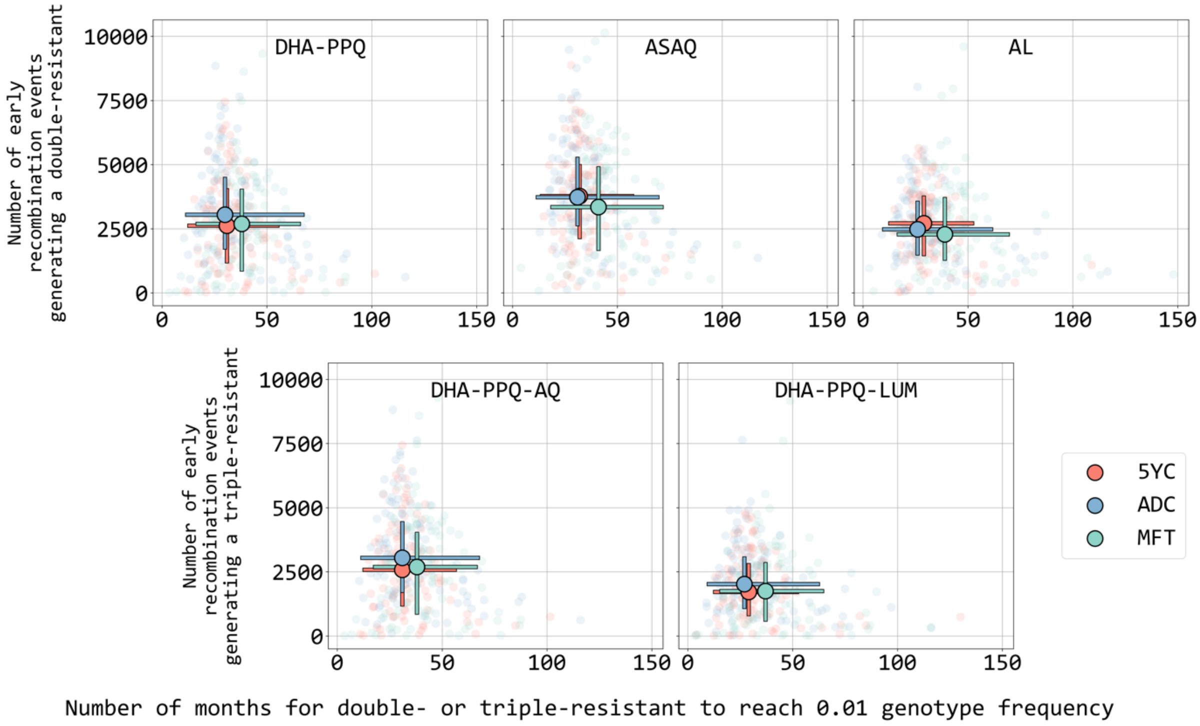

Figure J: Number of *early recombination events* (y-axis) creating a double-resistant or triple-resistant genotype (see text inside each panel). An *early recombination event* is one that occurs prior to the time that the frequency of this MDR genotype reaches 0.01. The format of this figure is the same as Figure 6 in main text but $c_R = 0.005$. Circles show the median values for three strategies: five-year LMS cycling in red (5YC), adaptive LMS cycling in blue (ADC), and multiple first-line therapies in green (MFT). Bars show interquartile ranges, and the full set of outcomes is shown as a light scatterplot in the background. The x-axes count number of months *after first appearance*.

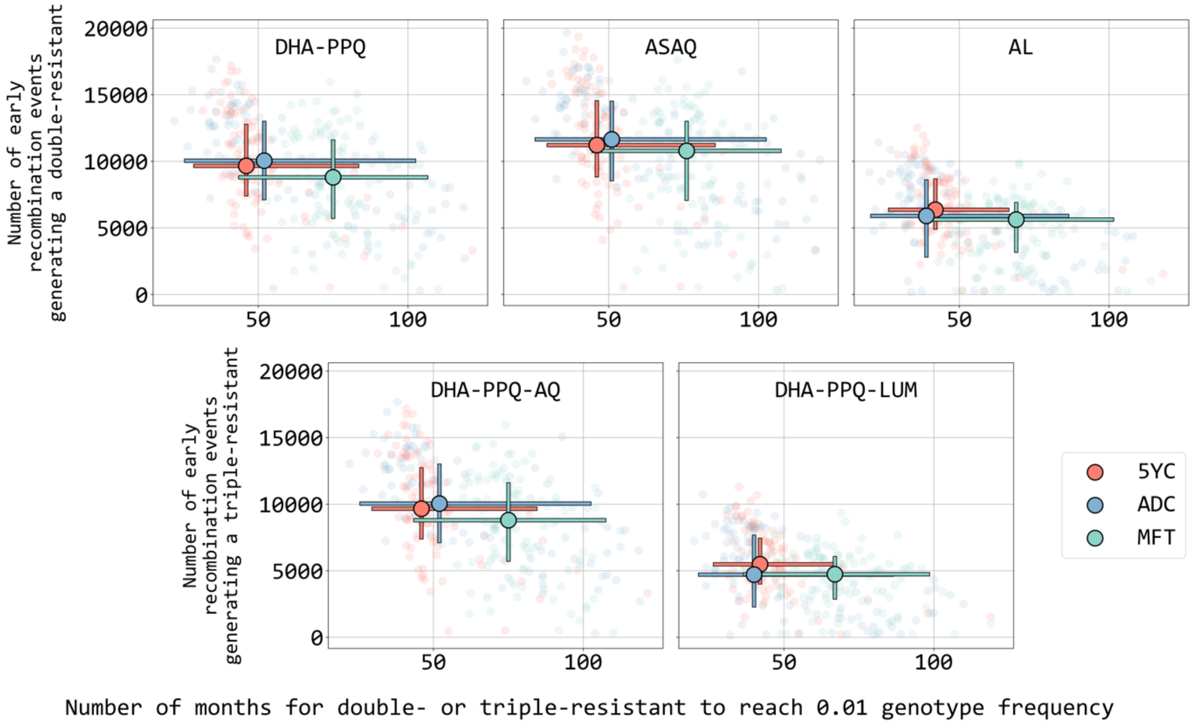

Figure K: Number of *early recombination events* (y-axis) creating a double-resistant or triple-resistant genotype (see text inside each panel). An *early recombination event* is one that occurs prior to the time that the frequency of this MDR genotype reaches 0.01. The format of this figure is the same as Figure 6 in main text but prevalence is 25% and $c_R = 0.0005$. Circles show the median values for three strategies: five-year LMS cycling in red (5YC), adaptive LMS cycling in blue (ADC), and multiple first-line therapies in green (MFT). Bars show interquartile ranges, and the full set of outcomes is shown as a light scatterplot in the background. The x-axes count number of months *after first appearance*.

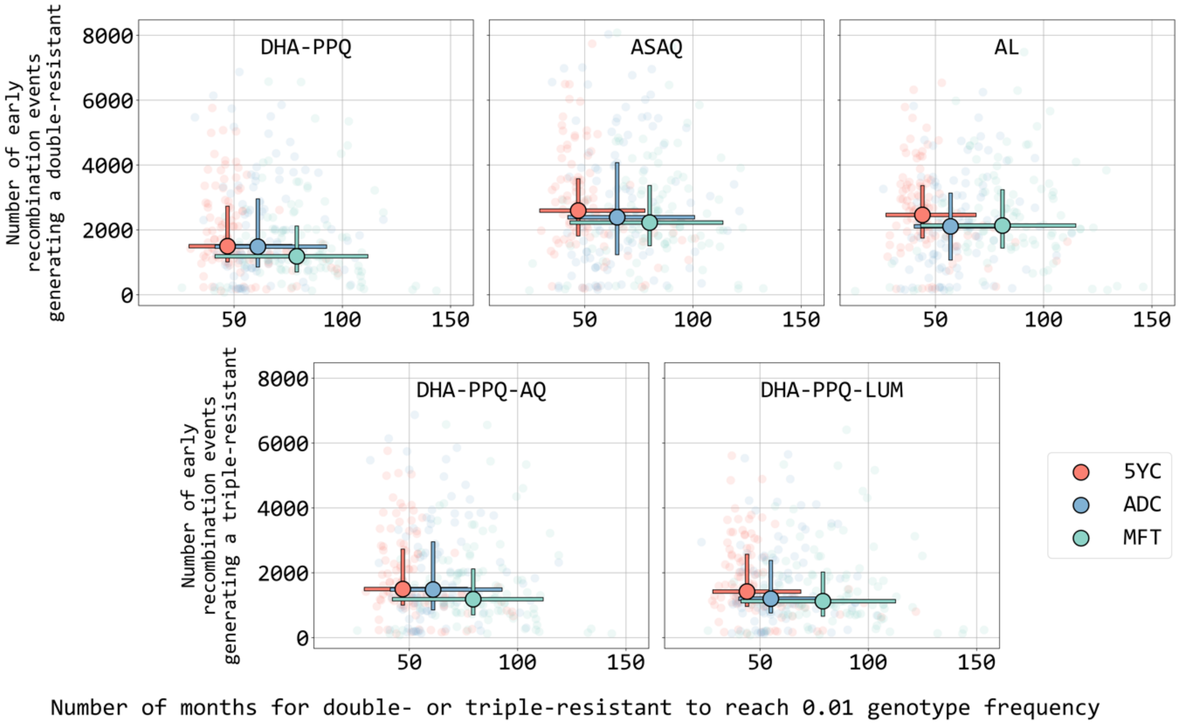

Figure L: Number of *early recombination events* (y-axis) creating a double-resistant or triple-resistant genotype (see text inside each panel). An *early recombination event* is one that occurs prior to the time that the frequency of this MDR genotype reaches 0.01. The format of this figure is the same as Figure 6 in main text but prevalence is 25% and $c_R = 0.005$. Circles show the median values for three strategies: five-year LMS cycling in red (5YC), adaptive LMS cycling in blue (ADC), and multiple first-line therapies in green (MFT). Bars show interquartile ranges, and the full set of outcomes is shown as a light scatterplot in the background. The x-axes count number of months *after first appearance*.

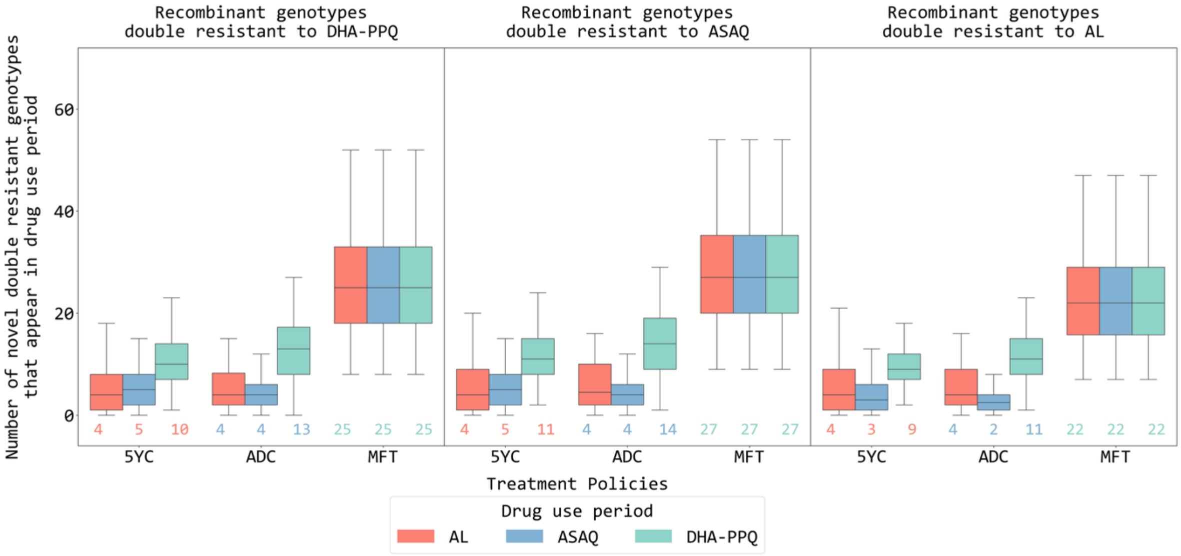

Figure M: The y-axis shows the number of unique double-resistant genotypes produced by recombination under each strategy. The boxplot colors show under which 'drug use period' this new genetic diversity was generated. For MFT the entire 15-year period is considered a 'drug use period' for each of the three ACTs. The visual comparisons to make are red box plots to red box plots, blue box plots to blue box plots, etc. Cycling strategies are LMS. In this figure the prevalence is 5% with daily cost of resistance is 0.0005 and mosquito cohort size is 100 with 20% interrupted feeding rate. Under MFT, recombination generates a greater diversity of genotypes (when compared to cycling strategies) but (1) there are fewer of these recombination events under MFT, and (2) these different recombinant genotypes have a slower evolutionary path to 0.01 genotype frequency (see Figures J-L).

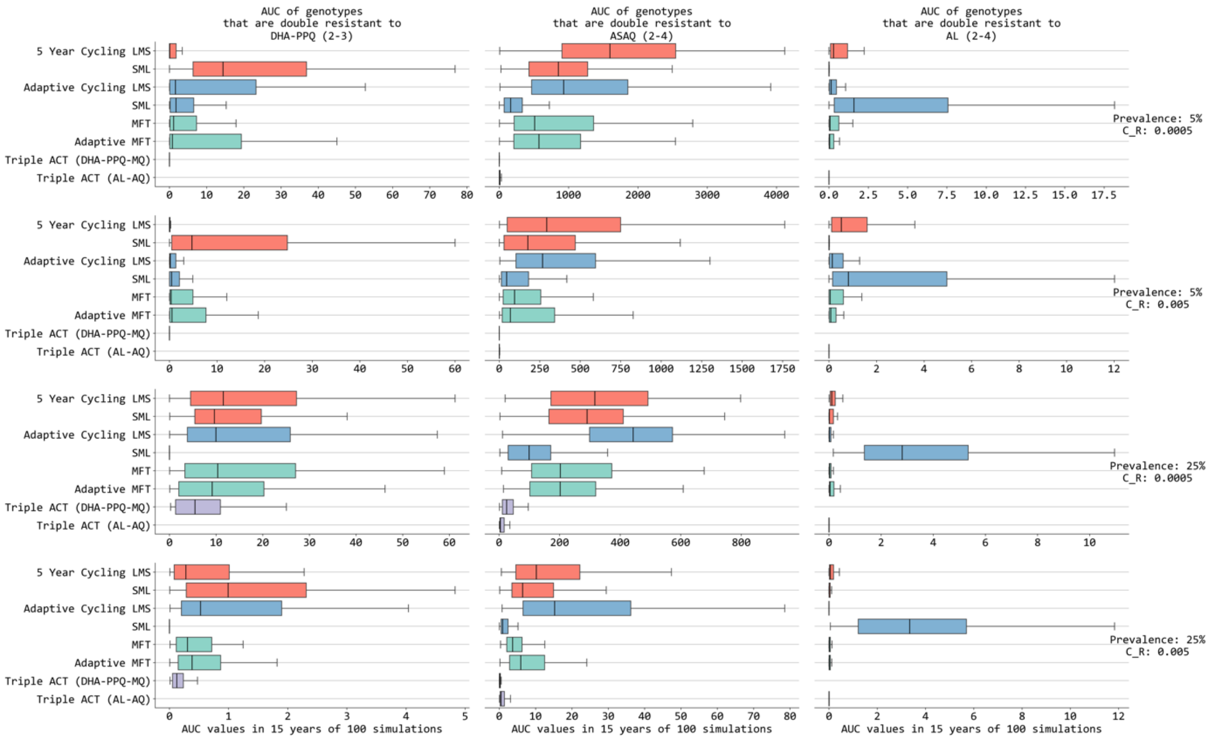

Figure N: AUC (total area under the frequency curve of a maximally-resistant genotype) of genotypes that are double resistant to DHA-PPQ, ASAQ, and AL (in the three columns). This is the same AUC measure of multi-drug resistance risk as presented in Figures 4 and 5 or Li et al (*Nat Commun*, 15:1390, 2024). The codes "2-3" and "2-4" at top indicate that the genotypes are double-resistant (first digit) to a specific drug with 3 or 4 mutations (second digit). The four rows show different malaria scenarios with different prevalence and cost of resistance. In each panel, the *x*-axis shows the AUC values of double-resistant genotypes under different treatment strategies. Different colors are for different treatment policies: 5-year cycling (light red), adaptive cycling (light blue), multiple first-line therapies (sea green) and triple ACT (violet). LMS indicate the order of drugs used in cycling strategies based on half-life of partner drugs (Long: DHA-PPQ, Medium: ASAQ, Short: AL). When no boxplot is shown this means that no double-resistant genotypes were generated.

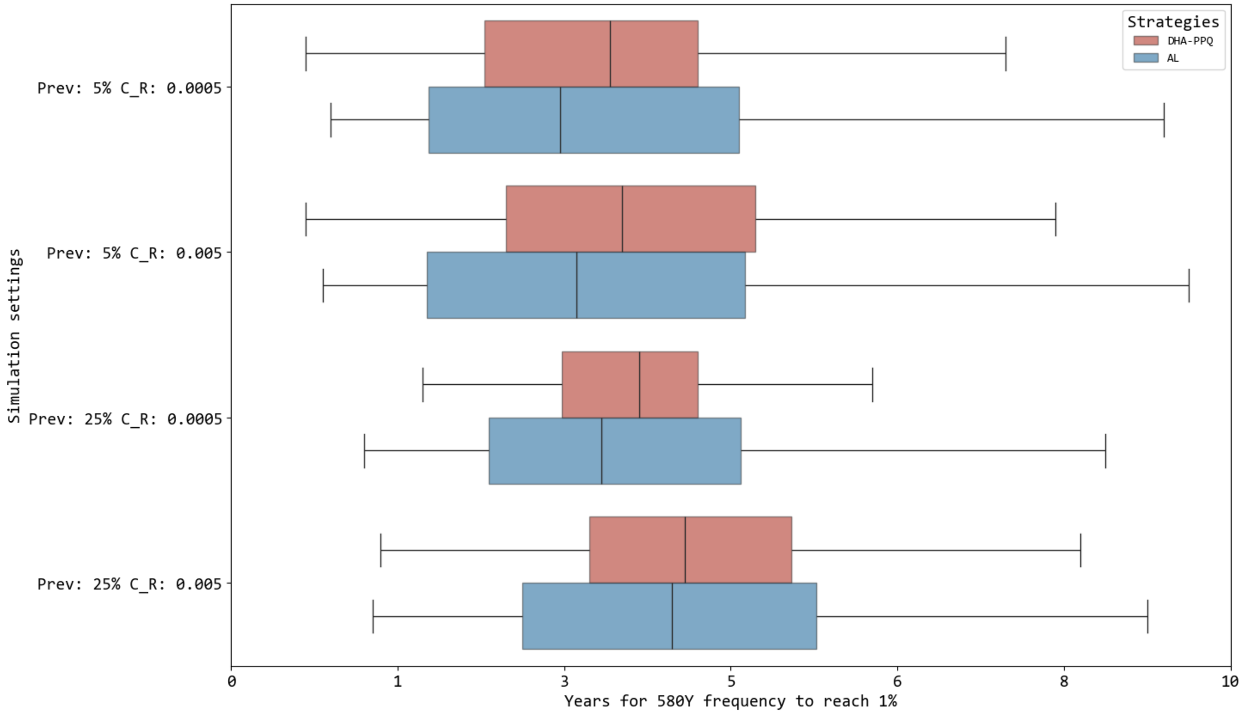

Figure O: Time to 0.01 allele frequency of 580Y mutation in four epidemiological settings using single first-line therapy (red for DHA-PPQ and blue for AL). The *y*-axis shows the prevalence and c_R settings while the *x*-axis shows the number of years to reach 0.01 frequency.

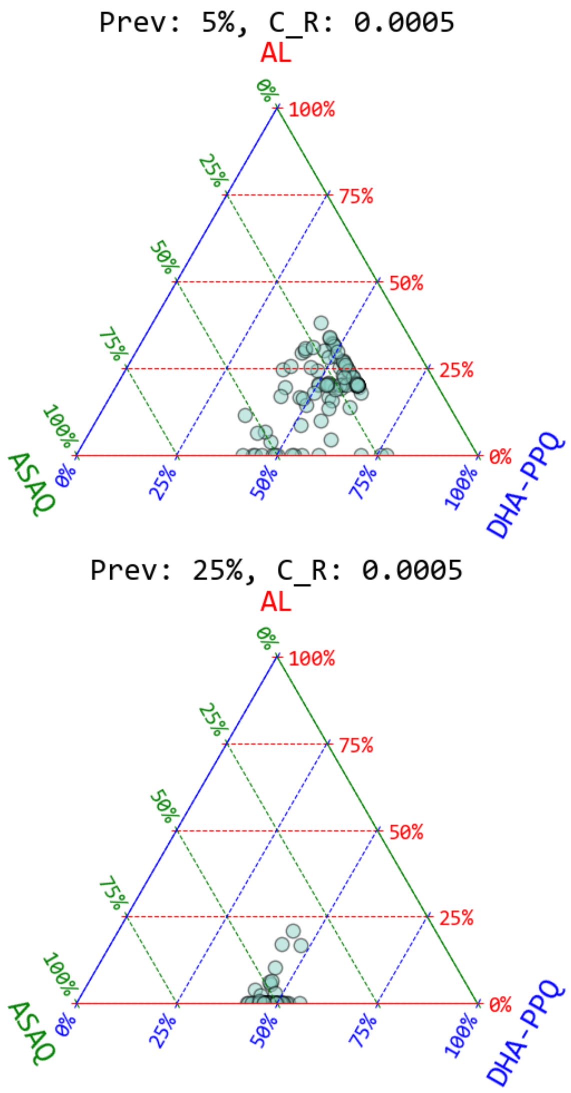

Figure P: Barycentric plot of drug-use percentage in LMS adaptive cycling strategies over 15 years. Top, left, and right vertices correspond to the therapies used. Each dot corresponds to one simulation. Note that AL appears to be used for less time than the other therapies, as it is third in sequence in an LMS approach. At 25% prevalence and $c_R = 0.005$, DHA-PPQ is used for the majority of the 15-year period, after which there is a switch to ASAQ which is used through year 15 with no final switch to AL during this period.

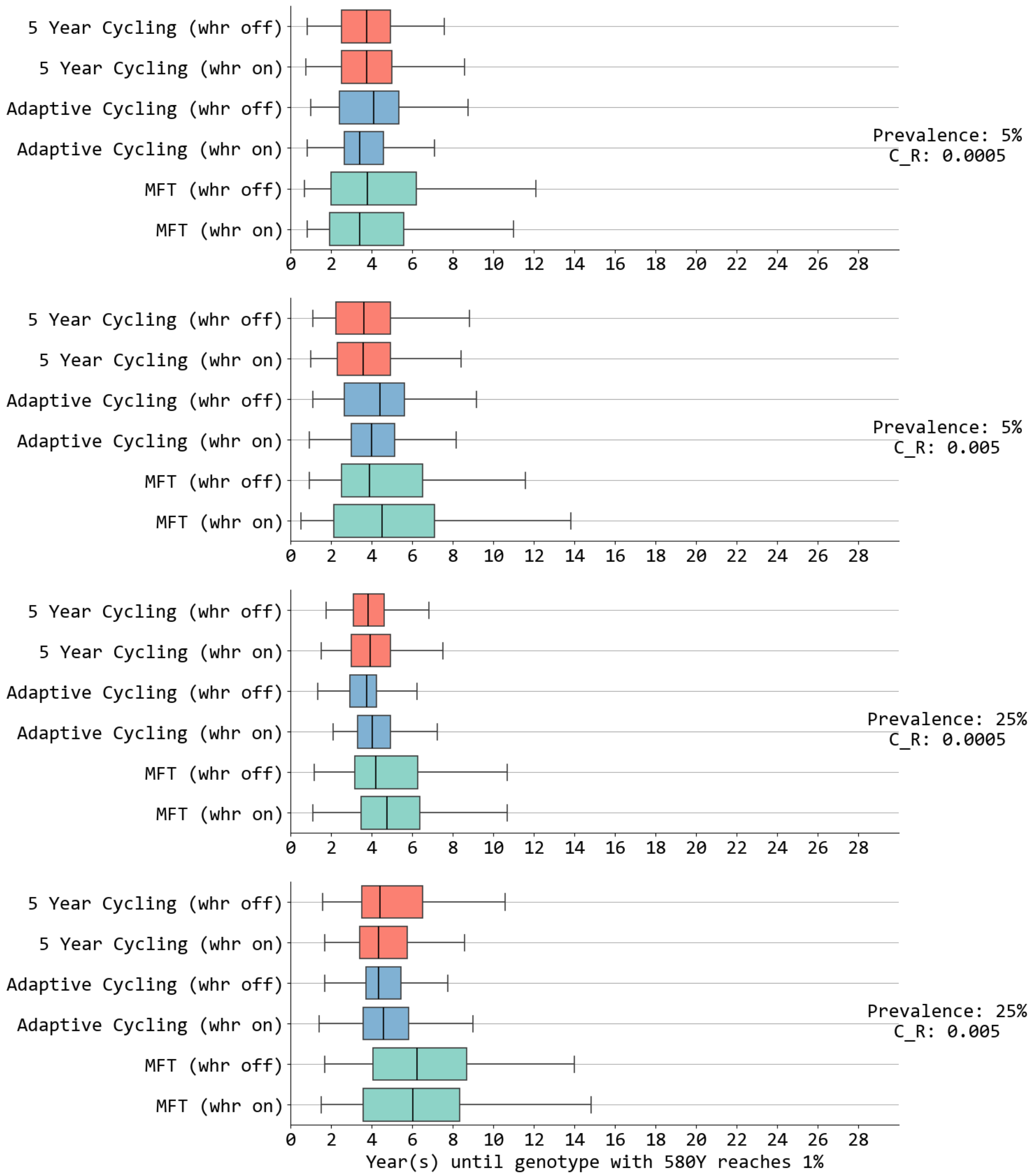

Figure Q: Number of years (x-axis) until a single-mutation pfkelch13 variant reaches 0.01 allele frequency. Results are shown for four different prevalence and cost-of-resistance settings. Different colors are for different treatment strategies: 5-year cycling (red), adaptive cycling (blue) and multiple first-line therapies (green). LMS indicates the order of drugs used in cycling strategies based on half-life of partner drugs (**L**ong: DHA-PPQ, **M**edium: ASAQ, **S**hort: AL). Setting with recombination from multi-clonal within-host parasites infection is indicated with “whr on” or “whr off” labels. The mosquito cohort size is 100 and the interrupted feeding rate is 0%.

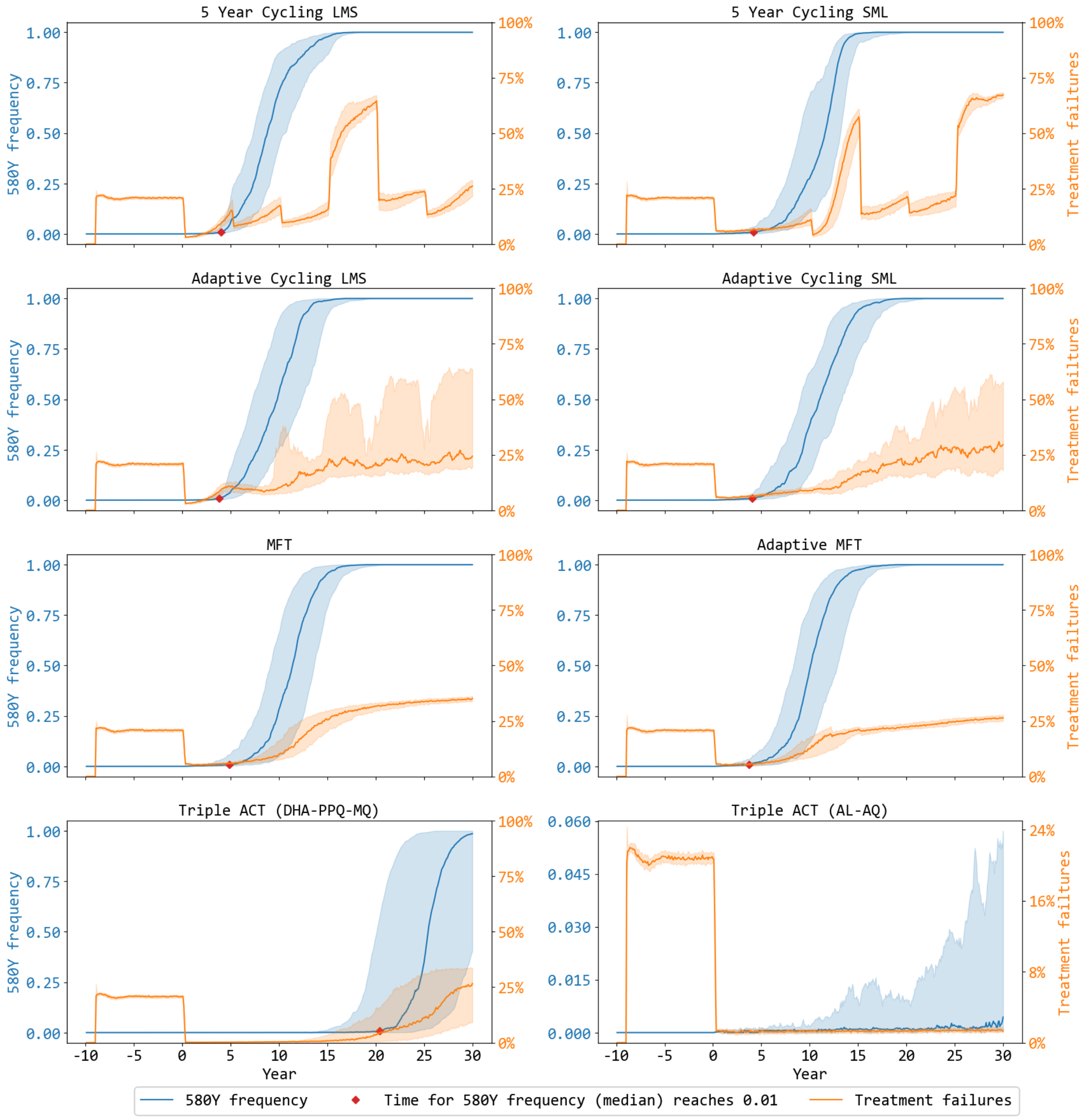

Figure R: Frequency of single-mutation pfkelch13 variant and treatment failure rate of the population. Each panel corresponds to a specific treatment strategy. Blue line indicates the median of 580Y frequency while orange line shows the median of treatment failure rate in a 60-day window over time. Shading area shows IQR of 580Y frequency and treatment failure rate, respectively. Red diamond indicate time where median of 580Y frequency reach 1%. LMS indicates the order of drugs used in cycling strategies based on half-life of partner drugs (**L**ong: DHA-PPQ, **M**edium: ASAQ, **S**hort: AL). In each panel, the left y-axis is the frequency of 580Y alleles while the right y-axis indicates the treatment failure rate. The setting of this plot is 5% prevalence with C_R=0.0005, the mosquito cohort size is 100 and the interrupted feeding rate is 20%.

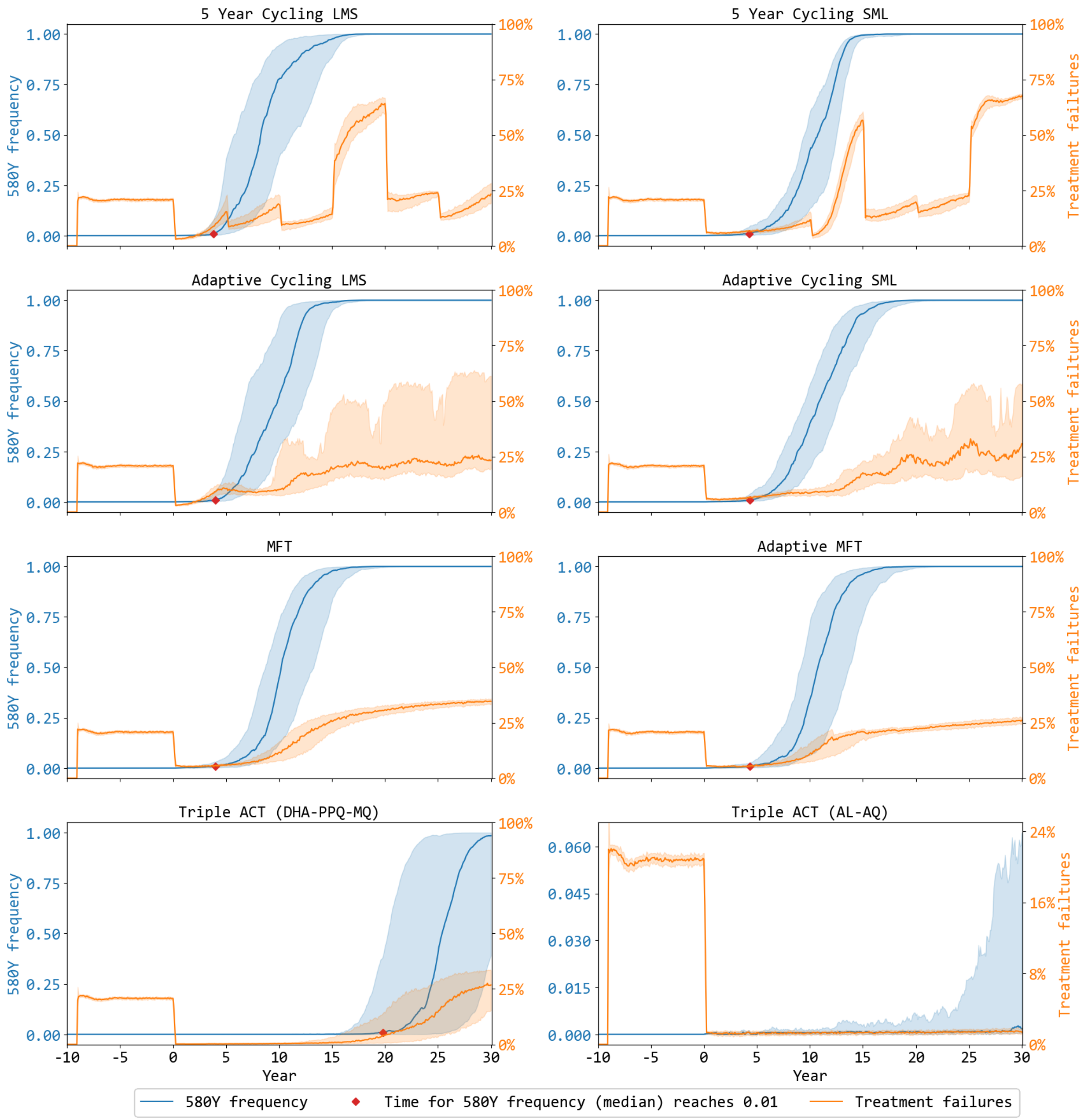

Figure S: Frequency of single-mutation pfkelch13 variant and treatment failure rate of the population. Each panel corresponds to a specific treatment strategy. This plot has the same settings and illustration as Figure R above and the only difference is the interrupted feeding rate is set to 0%.

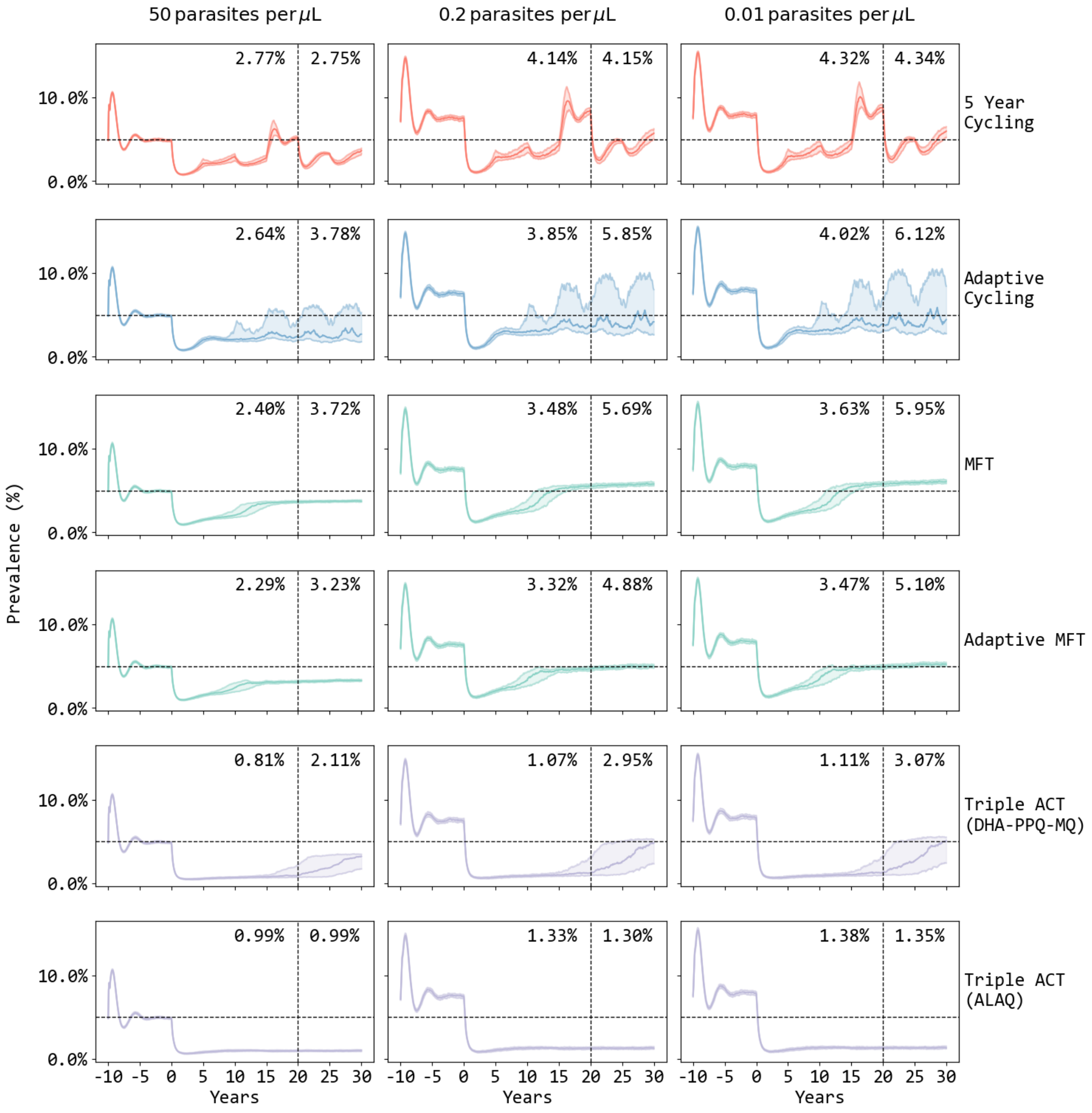

Figure T: Prevalence of different treatment strategies under different detectability thresholds for parasites per microliter. The left column shows prevalence based on 50 parasites per microliter and the middle and right columns are for 0.20 and 0.01 parasites per microliter, respectively. Horizontal dashed line indicates the prevalence at equilibrium and vertical dash line indicates year 30 of simulation. Different colors are for different treatment strategies: 5-year cycling (red), adaptive cycling (blue), multiple first-line therapies (green) and triple ACT (violet). The larger the gap between thresholds, the larger the gap between reported prevalence. There is only a minor different between prevalence based on 0.2 and 0.01 parasite thresholds. This indicates that majority of detected cases has parasite density exceed 0.2 parasites per microliter. The mosquito cohort size is 100, interrupted feeding rate is 20% and C_R=0.0005. The prevalence at equilibrium in this setting is 5%. The number to the left of each vertical dashed line is the median of mean prevalence from year 0 to 20, while the number to the right of the dashed line indicates the median of mean prevalence from year 20 to 30.

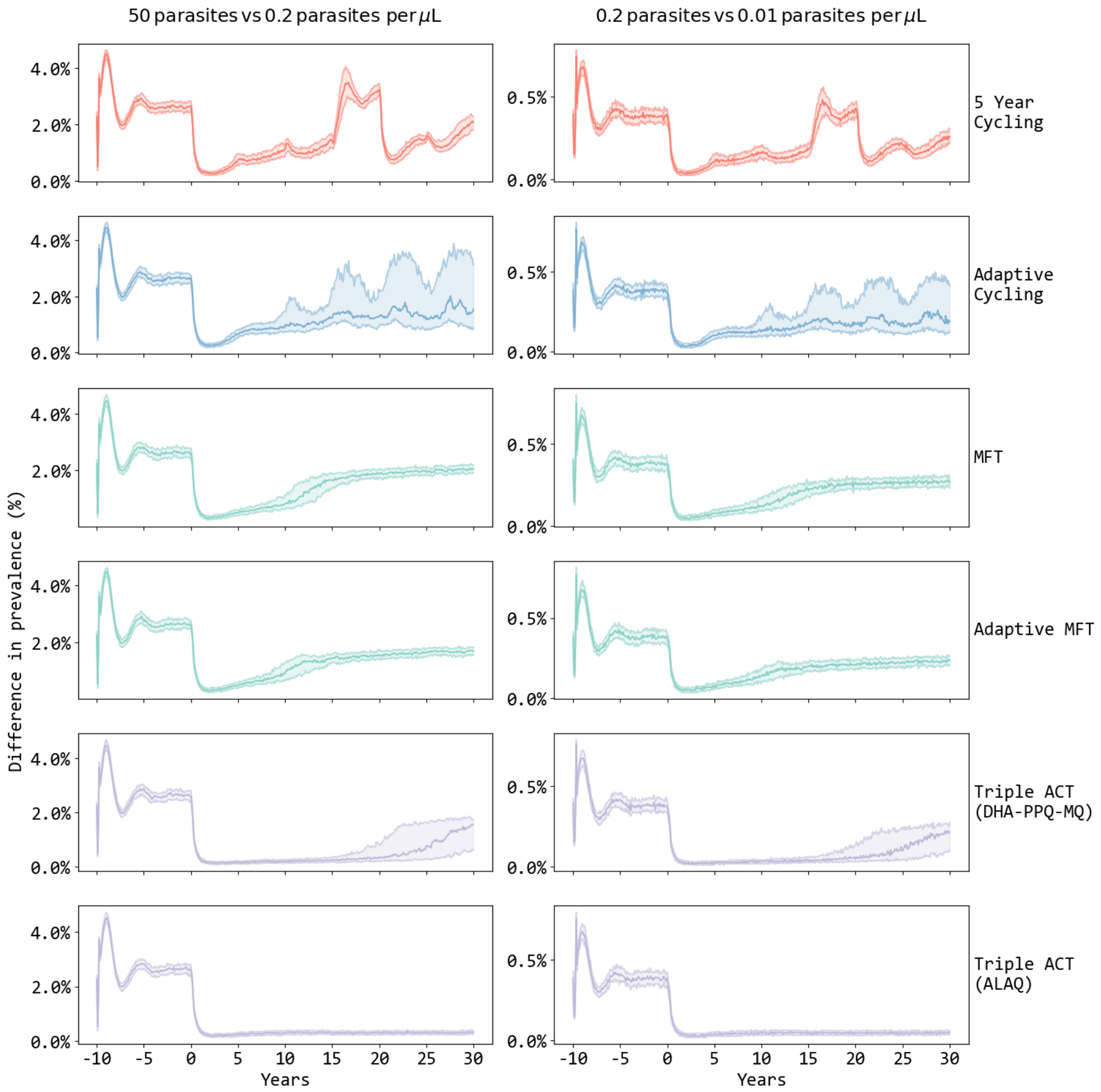

Figure U: Absolute difference in measured prevalence between 50 and 0.2 parasite-per-ul thresholds (left panels), and 0.2 and 0.1 parasite-per-ul thresholds. The difference on the *y*-axis is the absolute value between two prevalences measured with different sensitivities. Different colors are for different treatment strategies: 5-year cycling (red), adaptive cycling (blue), multiple first-line therapies (green) and triple ACT (violet). The mosquito cohort size is 100, interrupted feeding rate is 20% and C_R=0.0005. The difference drops close to zero after year zero, as everyone received high efficacy ACTs after burn-in, and nearly all parasites for most symptomatic cases.
